# Transcriptional activity during seed imbibition is conserved, robust and required for germination

**DOI:** 10.1038/s41467-026-76795-8

**Published:** 2026-08-19

**Authors:** Michal Krzyszton, Veena Halale Manjunath, Lien Brzezniak, Sargam Bharti, Martyna Katarzyna Adach, Silviya S. Lal, Patrycja Witek, Dariusz J. Smolinski, Szymon Swiezewski

**Affiliations:** 1 Laboratory of Seeds Molecular Biology, Institute of Biochemistry and Biophysics of the Polish Academy of Sciences, Warsaw, Poland; 2 Doctoral School of Molecular Biology and Biological Chemistry, Institute of Biochemistry and Biophysics of the Polish Academy of Sciences, Warsaw, Poland; 3 Department of Cellular and Molecular Biology, Nicolaus Copernicus University, Torun, Poland; 4 Institute of Advanced Studies, Nicolaus Copernicus University, Torun, Poland

**Keywords:** Plant development, Transcription, Plant reproduction

## Abstract

In most plants, germination begins with the reactivation of metabolism during water uptake by the desiccated seed. Notably, Pol II transcription is hypothesised not to be required for early germination, as seeds appear to initially rely on stored mRNAs produced during their maturation. Here, we show that efficient blocking of Pol II transcription completely stops germination in Arabidopsis seeds. Pol II activity is initiated early and is widespread, following expression and Pol II binding patterns established during seed drying. Our findings suggest that the earliest transcriptional phase relies on pre-existing protein factors. The transcriptional programme initiated during water uptake is robust across diverse imbibition conditions and in seeds matured at different temperatures, and is conserved in rapeseed (*Brassica napus*). Only after the successful completion of this initial phase and the passing of the translation-dependent checkpoint do seeds activate genes that lead to germination. Our results redefine the role of transcription in seed germination and highlight a conserved pattern of early transcriptional activation.

## Abstract (web summary)

Seeds are known to accumulate mRNA, and it has been suggested that they do not need Pol II transcription early in germination. Here the authors show that new mRNA synthesis starts shortly after water absorption and is crucial for seed germination.

## Introduction

In their dry, mature state, orthodox seeds become metabolically inactive due to severe water depletion^1^. This regulated shutdown, occurring during the final stages of seed maturation, involves turning off transcription^2,3^, a key step in gene expression. Although likely reduced due to increased chromatin condensation^4^, RNA polymerase II’s (Pol II) transcriptional activity may still occur at the late seed maturation stage^2^. This could play a role in preparation for germination, as mutants in various factors involved in Pol II elongation exhibit defects in seed physiology and morphology^5–9^. However, the status of Pol II complexes in dry seeds and the timing of transcription restart during seed imbibition (water uptake) remain unclear. The strong compaction of chromatin in mature seeds^4^ may suggest the removal of Pol II from the DNA, followed by its re-recruitment later during imbibition. This is supported by findings indicating that seed imbibition with α-amanitin, a Pol II inhibitor, does not block radicle protrusion in Arabidopsis and rice. In contrast, stopping translation completely halts any morphological changes related to germination^10–12^. These observations suggest that during germination, seeds can rely on stored mRNAs synthesised throughout their maturation^13–15^. In conclusion, it is assumed that Pol II activity during the early stages of germination may be dispensable^10,12^, reinforcing the role of stored mRNAs in supporting the initial steps of seedling establishment.

Reported limited Pol II activity during early germination is inconsistent with the fact that many mRNAs show upregulation between 1 and 3 h of imbibition in Arabidopsis and rice^15,16^. In agreement, some Pol II complexes remain attached to chromatin in dry seeds, potentially enabling rapid transcription reactivation during imbibition^2,17,18^. A study on *Brassica napus* (rapeseed) demonstrated Pol II presence on DNA and its transcriptional competence using chromatin from desiccated seeds^2^. Pol II activity on the tested genes significantly decreased during desiccation but resumed quickly upon imbibition^2^. Recent studies confirmed the occupancy of Pol II on selected genes in mature Arabidopsis seeds using ChIP-qPCR^17,18^ and sequencing of capped short RNAs (csRNA)^17^. csRNAs are likely generated by promoter-proximal paused Pol II, offering an indirect method to identify genes primed for transcription^19^. Analysis of csRNAs in dry Arabidopsis seeds uncovered thousands of such genes, which largely coincide with those producing stored mRNAs during seed maturation^17^. These findings imply that, similar to translation, early Pol II transcription during imbibition may recapitulate the gene expression patterns from seed maturation^2,10,17^. Only later are mRNAs characteristic of the seed maturation stage downregulated, and the transcriptional programme for seed germination is initiated^20^. Consequently, it is possible that early during imbibition, gene expression is directly influenced by regulators inherited from the maturation stage. It is widely recognised that environmental conditions during seed maturation significantly affect seed properties (reviewed in ref. ^21^). Among them, low temperatures during seed maturation exert the most substantial influence on seed dormancy^22–24^. Whether the transcription of genes induced by the maturation environment would persist during imbibition remains an open question.

The significance of early transcription during imbibition remains largely unexplored, although it has been highlighted as an important question in several studies^17,25,26^. In this work, by using seed coat nicking in Arabidopsis, we enhanced the uptake of α-amanitin and modified nucleotides into the seed and demonstrated that Pol II is transcribing during early germination. The labelling of newly synthesised mRNAs revealed that transcription during early water uptake is widespread and largely recapitulates seed maturation gene expression. We show that during imbibition, two phases of gene expression take place. The early phase is robust and affected only to a limited extent by the imbibition temperature, exogenous ABA, or antabactin, an inhibitor of ABA signalling^27^. Pol II transcription profile during imbibition is also maintained in seeds matured at varying temperatures, although these conditions leave a lasting imprint on the stored mRNA pools. Importantly, the early transcription patterns are conserved in *Brassica napus* seeds. Together, these findings indicate that two transcriptional shifts are executed early during imbibition. The first is related to seed desiccation response and is not blocked by translation inhibition. The second drives the transition to germination and is partially dependent on active translation, making it more responsive to external cues.

## Results

### Inhibition of Pol II during early imbibition blocks germination

**Fig. 1:**
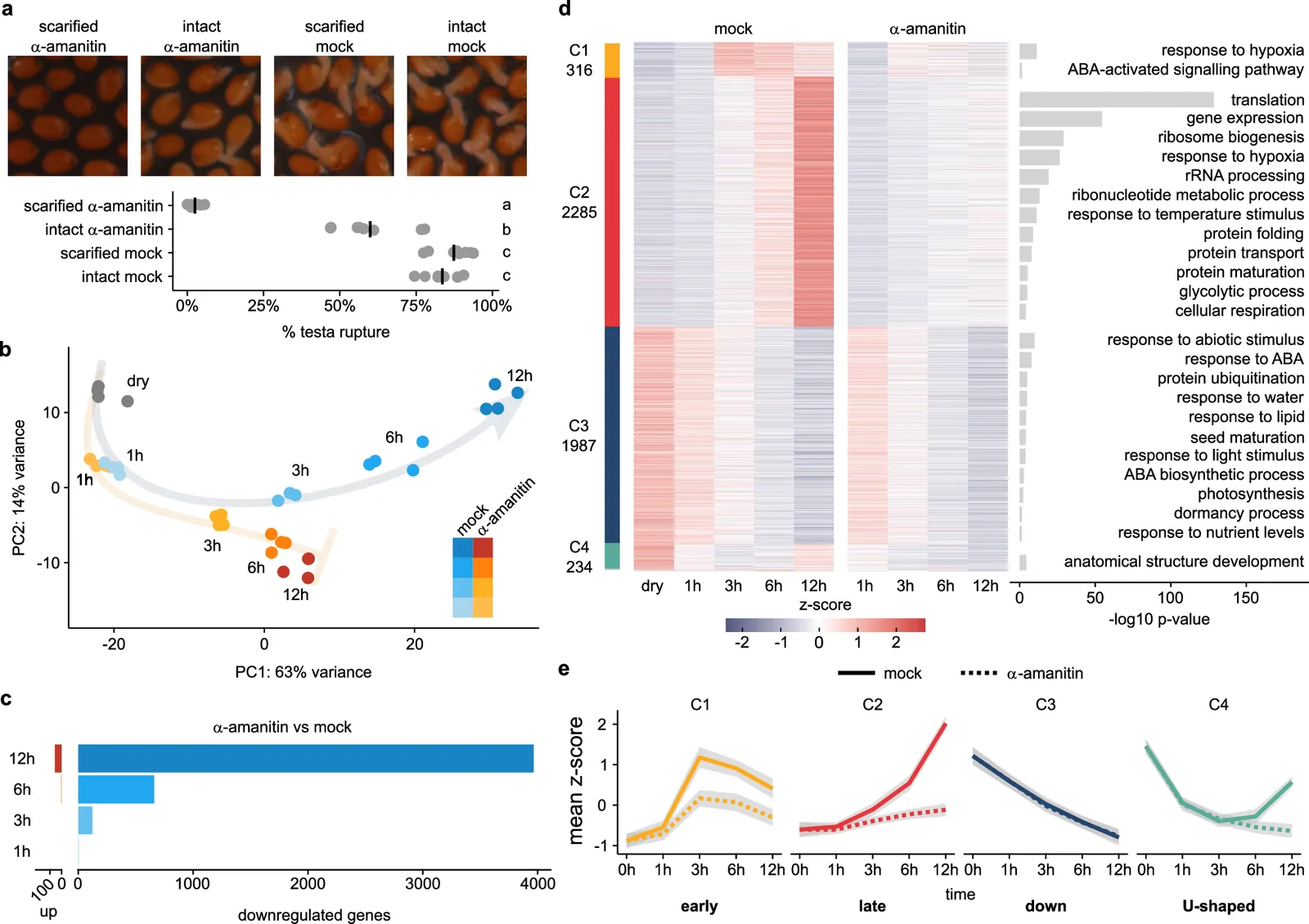
α-amanitin inhibits Pol II transcription during early seed imbibition. **a** Germination phenotypes of seeds imbibed in α-amanitin and mock solutions, with their quantification (the fraction of seeds with testa rupture at 2 days after imbibition; *n* = 8 independent biological replicates; ≥50 seeds each—source data are provided as a Source Data file, which contains the exact number of seeds in each tested pool and *p*-values. Solid lines represent means; letters denote statistical differences (<0.05) using one-way ANOVA with Tukey’s post hoc test. **b** PCA plot summarising 3′RNA-seq results (*n* = 3–4; selected stable genes normalisation) from dry and imbibed seeds, with arrows manually added to show the changed transcriptomic trajectories. **c** Number of DEGs (DESeq2; selected stable genes normalisation;|log_2_FC| > 1, padj < 0.05) when comparing mock and α-amanitin-treated samples. **d** Grouping of DEGs from mock imbibition (WGCNA with z-score normalisation). The right heatmap displays the expression levels of the same genes during α-amanitin treatment. GO-term enrichment results are shown for representative biological process terms. **e** Average expression changes (line) for genes in clusters: C1–early upregulated, C2–late upregulated, C3–downregulated, unaffected by α-amanitin treatment. Shaded areas denote 95% confidence intervals.

Previous studies have demonstrated that α-amanitin fully inhibits Arabidopsis germination only after the root protrusion^10^. It is, however, unclear if this is not a result of the limited permeability of the seed coat during the initial stages of water absorption^12,28^, even when using seed coat-defective mutants^10^. To address this possibility, we tested whether seed scarification (nicking)^29^ could enhance the uptake of large molecules upon imbibition. Scarified seeds remain viable and can germinate (Supplementary Fig. 1a). Moreover, unlike intact seeds, scarified seeds were stained with tetrazolium red^23^ after overnight incubation (Supplementary Fig. 1b), confirming the uptake of large molecules. Next, we showed that, in scarified seeds but not in intact seeds, α-amanitin hampers the upregulation of specific mRNAs induced by imbibition (6 h) (Supplementary Fig. 1c). In fact, we observed a mild downregulation of some imbibition-induced mRNAs as early as 1 h of imbibition in scarified seeds in the presence of α-amanitin (Supplementary Fig. 1d). Importantly, in contrast to most intact seeds, scarified seeds did not exhibit testa rupture when incubated with α-amanitin (Fig. 1a). These results confirm that the seed coat prevents the uptake of α-amanitin, and the complete block of germination indicates an early requirement for Pol II.

Cordycepin is an adenine analogue and a broad-spectrum inhibitor that blocks Pol I/II/III, influences posttranscriptional RNA metabolism^30^, and also impacts signalling pathways^31^. It completely inhibited germination in both scarified and intact seeds (Supplementary Fig. 1e), consistent with previous reports^12^. It also reduced mRNA upregulation in scarified seeds, similar to α-amanitin (Supplementary Fig. 1f). We additionally tested germination under flavopiridol treatment, an inhibitor of CDKC;2 kinase^32^, which phosphorylates the Pol II C-terminal domain (CTD) and regulates Pol II activity^33^. Similar effects were observed in both scarified and intact seeds, with germination fully halted after testa rupture (Supplementary Fig. 1e). Although Pol II transcription declines upon flavopiridol treatment^33^, its inhibitory effect is probably weaker than that of α-amanitin, which blocks Pol II elongation and targets it for degradation^34^. Due to its high specificity and irreversible activity, we chose to use α-amanitin in a genome-wide experiment.

We investigated how the blocking of Pol II activity impacts transcriptomic changes during the initial stages of imbibition using the 3′RNA-seq protocol^35^. We sequenced the 3′ ends of mature mRNAs from scarified seeds in the dry state and after imbibition (1, 3, 6 and 12 h) in the presence or absence of α-amanitin. Transcription blocks usually lead to unidirectional changes in mRNA levels and thus create a challenge to global normalisation^12,36^. Therefore, as described by others^36,37^, normalisation was performed using a selected set of stable mRNAs that met two criteria. (1) Their level should appear to increase during imbibition in inhibitor-treated samples compared to mock, as their relative abundance in the library rises due to the degradation of unstable mRNAs^36^. (2) They should not be affected by imbibition in mock-treated seeds (see Supplementary Bioinformatic Methods for a detailed description and Supplementary Data 1 for the selected gene list). The analysis performed after such renormalisation revealed the Principal Component Analysis (PCA) map with progressive transcriptomic changes in mock-treated seeds from the dry state to 12 h of imbibition. Notably, the effect of α-amanitin treatment became evident as early as 3 h after imbibition and became more pronounced over time (Fig. 1b). The transcriptomes of seeds treated with α-amanitin appeared similar at 12 h compared to 6 h, suggesting transcriptional arrest (Fig. 1b). Differential gene expression analysis revealed numerous genes (DEGs) with either upregulated or downregulated expression during mock imbibition, while α-amanitin treatment primarily caused downregulation of expression (Fig. 1c, Supplementary Fig. 1g and Supplementary Data 1).

We used WGCNA to identify gene expression modules (clusters) among all genes with altered expression in mock-treated seeds (Supplementary Data 1), which revealed four gene groups (Fig. 1d). Cluster C1 displayed early activation (at 3 h) and was enriched in abiotic stress-responsive genes, especially those induced by hypoxia (Fig. 1d and Supplementary Data 1). We hypothesise that this may be related to the repair of damage caused by desiccation and dry seed storage^38^. The induction of the hypoxia response may also reflect the high energy demand associated with transitioning from a dry state^39^. Cluster C2 showed a gradual increase in expression (late activation) and consisted of genes linked to translation, nucleotide biosynthesis and respiration (Supplementary Data 1). Cluster C3 included genes whose expression decreased during imbibition and was enriched in functions related to ABA, seed maturation and photosynthesis (Supplementary Data 1). Finally, cluster C4 contained a subset of genes whose expression initially declined but was reinduced at 12 h. When examining C1–C4 genes in α-amanitin-treated seeds, we observed a marked reduction in gene expression upregulation, while downregulation of mRNA levels remained unaffected (Fig. 1d, ed). We confirmed these findings by comparing the fold changes of genes affected at the 12-h time point with or without α-amanitin (Supplementary Fig. 1h, i).

In conclusion, we show that efficient inhibition of Pol II transcription during early imbibition leads to a complete block of seed germination. Our analysis reveals two waves of mRNA upregulation during imbibition: one peaking at 3 h (C1–early) and the second increasing until 12 h (C2–late). Because both are blocked by α-amanitin treatment, this shows that they are driven primarily by new mRNA production.

### 4-SU mRNA labelling in seeds

Our α-amanitin treatments suggest Pol II transcription starts early during imbibition. This is supported by the rapid induction of genes belonging to C1—the early cluster (Fig. 1e). However, determining the exact timing of gene transcription activation is challenging due to the abundance of stored mRNAs. To address this, we opted for labelling newly synthesised RNAs with 4-thiouridine (4-SU). The labelling of mRNAs with 4-SU during imbibition is enhanced when the seed coat is nicked. This was validated using (1) biotinylation of 4-SU, followed by streptavidin enrichment^40^ (Supplementary Fig. 2a), and (2) conversion of 4-SU to cytidine^41^, followed by Sanger sequencing (Supplementary Fig. 2b).

**Fig. 2:**
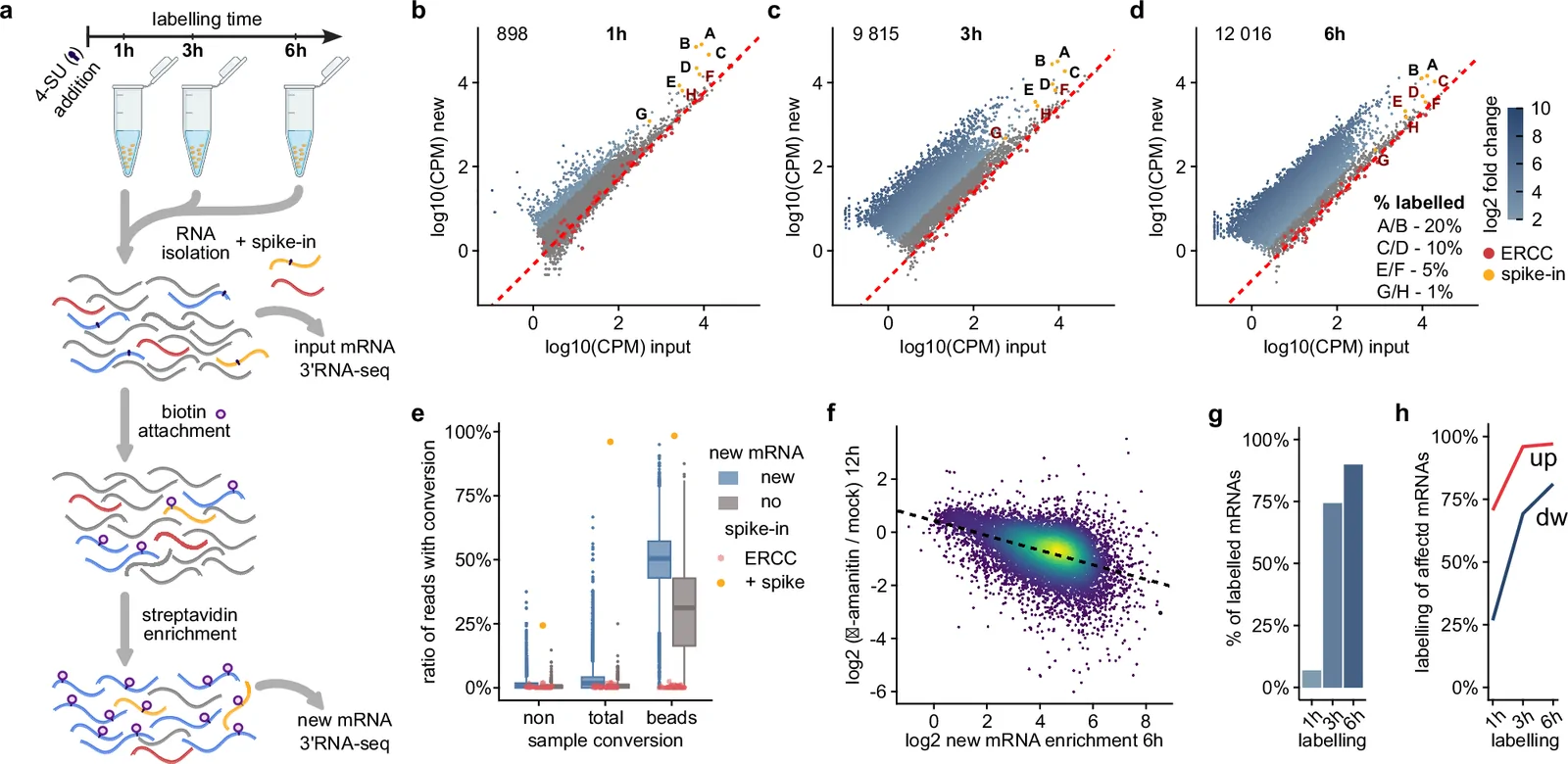
Widespread production of new mRNAs during seed imbibition. **a** Diagram demonstrating an mRNA labelling experiment. Created in BioRender. Halale manjunath, V. (2026) https://BioRender.com/vee1ryv. **b–d** Plots comparing input and enriched mRNAs after 4-SU labelling, with identified new mRNAs highlighted in blue and their number indicated on plots (DESeq2; *n* = 4; ERCC-based normalisation; padj < 0.05; log_2_FC > 2; red line—the trend for ERCC). Yellow dots indicate labelled spike-ins, with the black colour of the spike-in name denoting that it was enriched over log_2_FC > 2. New mRNAs were identified for **b** 1 h, **c** 3 h and **d** 6 h of imbibition. **e** Ratio of reads with at least one T to C conversion in control (non-chemically converted), total and streptavidin-enriched mRNAs (DESeq2; *n* = 4—biological replicates used for library preparation; ERCC-based normalisation; padj < 0.05; log_2_FC > 2). Boxplots show the ratio for new mRNAs that were called new or not in this experiment. Spike-ins are presented as separate dots: ERCC—red; positive (100% labelled with 4-SU)—yellow. Boxplot central line represents median, whiskers represent 1.5 times the interquartile range; outliers are indicated with dots. **f** Comparison of new mRNA production and the effect of α-amanitin treatment (point density is illustrated with the Viridis scale; the line represents a trend). **g** The proportion of genes with mRNA in the input (>1 CPM) identified as producing new mRNAs. **h** Percentage of DEGs (time point vs dry) showing mRNA production.

To gain a global view of transcriptional activity during imbibition, we enriched 4-SU-labelled new mRNAs after 1, 3 and 6 h using biotin attachment and streptavidin pulldown^40,42^, followed by 3′RNA sequencing of enriched and input (unenriched) samples (Fig. 2a). Because nonspecific binding is a common confound in pulldown analysis^43^, we added unlabelled ERCC spike-ins to each sample before RNA biotinylation. As these spike-ins carry no 4-SU label, their signal in pulldown samples reflects the level of nonspecific binding, giving us a baseline to distinguish true enrichment. As expected, ERCC spike-ins, though depleted in biotin-enriched samples compared to inputs, were still detected (Fig. 2b–d). We used this baseline to call new mRNAs with DESeq2, using the ERCC spike-in levels as the normalisation factor between enriched and input samples at each time point. This approach requires that a transcript’s enrichment exceed the nonspecific-binding background defined by the spike-ins, rather than simply being abundant in the enriched sample, and provides a statistical framework for analysis. Transcripts were called as new mRNAs if enrichment was both statistically significant (padj < 0.05) and at least fourfold relative to input. Because enrichment is measured relative to input rather than in absolute terms, this analysis identifies which transcripts are newly synthesised, but not what fraction of the total mRNA pool they represent.

To assess the sensitivity of our enrichment approach, we included eight positive-control spike-ins (T7-transcribed bacterial/human sequences) at four 4-SU labelled/unlabelled transcript ratios (20, 10, 5, 1%, each in duplicate), with the lowest detectable ratio at each timepoint indicating the protocol’s sensitivity limit. At 1 h, only the two lowest-ratio spike-ins (5 and 1%) fell below the fourfold enrichment threshold; by 3 h, this had extended to all 5 and 1% spike-ins, and by 6 h, only the 20% spike-ins remained enriched (Fig. 2b–d). This progressive loss of sensitivity suggests that increasing amounts of labelled endogenous mRNA compete with spike-ins during pulldown, likely leading us to underestimate new mRNA production at later time points.

To further validate the 4-SU labelling method, we quantified 4-SU-to-cytidine conversions^41^ in total and enriched samples after 3 h imbibition (Supplementary Fig. 2c–e), using unconverted total mRNA as a negative control for RT, PCR and sequencing errors. The conversion signal is inherently sparse: published estimates suggest that only a few nucleotides are incorporated per read of the new transcript, even for highly efficient labelling in cell cultures^44^. Taking this and high levels of stored mRNAs into account, it is clear why the ratios of converted nucleotides (Supplementary Fig. 2d) and reads containing them (Fig. 2e) are low in total mRNA samples. Despite this sparsity, illustrated by the difference in the ratios of conversions (number of U changed to C) (Supplementary Fig. 2d) and ratios of reads with conversion events (Fig. 2e), total mRNA samples showed a higher proportion of converted reads than the negative control (2.6 vs 1.17% on average; Fig. 2e). Even without conversion, labelling may increase RT error rate^44^, explaining the relatively high level of conversions in the negative control. Importantly, the total mRNA conversion ratios corresponded to the enrichment levels (Supplementary Fig. 2e). Conversion levels in enriched samples were much higher than in total (42% on average), orthogonally confirming the validity of our ERCC-based new mRNA identification pipeline (Fig. 2e and Supplementary Fig. 2d). This level of conversion in enriched mRNAs is most likely underestimation, as: (1) 3′RNA-seq samples only the transcript’s 3′ end, while labelling that enabled enrichment may occur in other parts of mRNA. (2) Conversion efficiency in our experiment may be relatively low, as exemplified by our fully labelled spike-in (25–44%; Supplementary Fig. 2d). For these reasons, and because of its low per-transcript sensitivity, we use enrichment rather than conversion as our primary criterion for calling new mRNAs.

### Early Pol II transcription is widespread and largely recapitulates seed maturation

After 1 h, 898 mRNAs showed enrichment compared to input samples (Fig. 2b). These included hypoxia-responsive mRNAs and known regulators of seed germination (Supplementary Data 2). After 3 h of labelling, the enrichment levels were higher and the number of enriched mRNAs increased to 9815 (Fig. 2c and Supplementary Data 2). After 6 h of incubation, this number increased to 12,016 genes (Fig. 2d and Supplementary Data 3). In addition, independent experiments yielded results that closely matched the new mRNA identified, confirming the reproducibility of our results (Supplementary Fig. 2f, g). Importantly, the enrichment of newly produced mRNAs was inversely correlated with the effects of α-amanitin on mRNA levels at later time points (Fig. 2f and Supplementary Fig. 2h). As expected, mRNAs with the strongest enrichment were the most downregulated by Pol II inhibition.

When new mRNA levels were compared at the early time points of germination, we observed almost exclusive upregulation between the 1- and 6-h time points (Supplementary Fig. 2i). This may suggest that, in addition to the gradual, ordered activation of gene expression upon imbibition, 4-SU-labelled mRNAs are potentially stabilised by the incorporated modified nucleotide. Although this could theoretically lead to an overestimation of transcription magnitude, it could also increase the likelihood of detecting the extent of new mRNA production. To control for 4-SU-induced mRNA stabilisation, we pulse-labelled newly synthesised RNA for 30 min after 2.5 or 5.5 h of imbibition. Due to limited uptake of chemicals by seeds at later stages of imbibition, this experiment was performed on squeezed-out seed embryos and coats. Analysis of these briefly labelled samples produced results highly consistent with continuous labelling (Supplementary Fig. 2j, k). Next, we performed 5-EU labelling (assumed to be less toxic than 4-SU^45^) followed by biotin attachment, enrichment, reverse transcription on beads and qPCR. Among the tested genes, most transcripts with FC > 4 (call threshold) on 4-SU 3′RNAseq showed results consistent with 5-EU RT-qPCR analysis (Supplementary Fig. 2l). Based on these results, we conclude that 4-SU treatment in imbibed seeds does not strongly affect the detection of new mRNAs.

The enriched samples showed reduced levels of highly abundant stored mRNAs, indicating that the 4-SU-mediated new mRNA isolation protocol employed does not simply reproduce the dry seed mRNA pool (Supplementary Fig. 2m). Most genes with mRNAs present in the input samples showed the production of new mRNAs at 3 and 6 h (Fig. 2g). This suggests that new transcription during early imbibition largely recapitulates the production of maturation-stored mRNAs. In line with this, even genes with decreased mRNA levels during imbibition showed new mRNA synthesis (Fig. 2h). However, some genes expressed during seed maturation are not transcribed during imbibition, as their mRNA enrichment profiles resemble those of spike-ins (Supplementary Fig. 2n). We identified 232 mRNAs with no enrichment at either the 3 or 6 h time point (log₂FC < 1 in spike-in-normalised data; Supplementary Data 2). These mRNAs are abundant in input samples (Supplementary Fig. 2o) but are downregulated during imbibition (Supplementary Fig. 2p), suggesting they are specific to seed maturation. Notably, the downregulation of many mRNAs in this group has been reported to commence during seed maturation, including late embryogenesis abundant (*LEA*) mRNAs and seed storage-related mRNAs^24^. This indicates that the limited transcription of these genes during imbibition reflects the continuation of the shutdown initiated during maturation. The existence of such seed maturation-associated non-transcribed genes supports the specificity of our new mRNA identification protocol. Furthermore, these non-transcribed genes proved useful in later experiments, serving as alternatives to spike-ins for detecting newly synthesised mRNAs. In fact, our analysis, as shown in the Supplementary Bioinformatic Methods, suggests that the identified internal controls may sometimes ensure higher reproducibility of the results^46^. However, we decided to use external controls, whenever possible, for clarity of the methodological approach.

In conclusion, the labelling of newly synthesised mRNAs demonstrated that the restart of Pol II transcription during imbibition occurs early and is widespread. Most genes expressed during seed maturation continue to be transcribed during early imbibition, reflecting the persistence of transcriptional patterns rather than the rapid initiation of a novel transcriptional programme.

### Differential effects of translation block on new mRNA levels

**Fig. 3:**
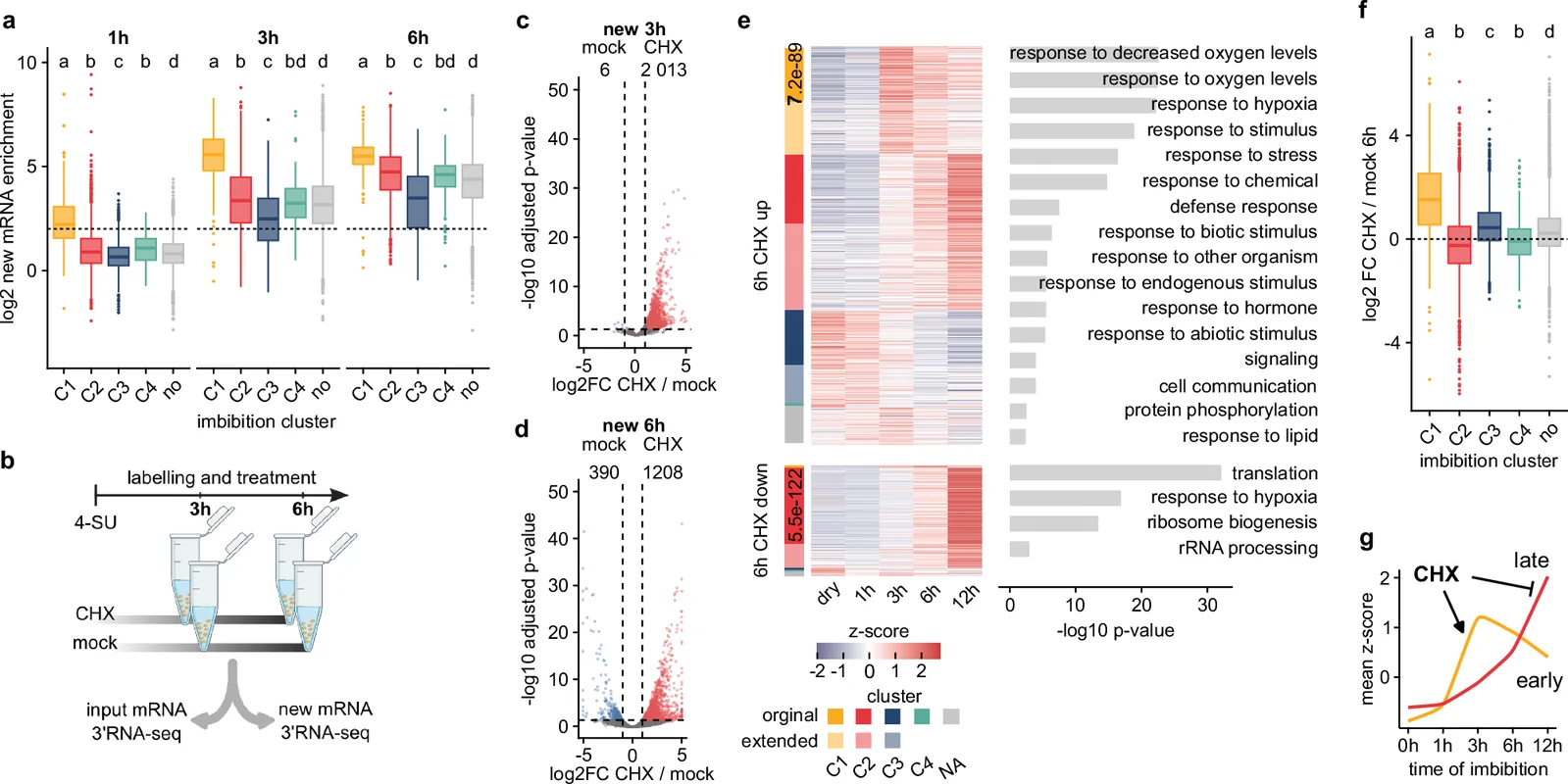
New mRNA production during seed imbibition has two phases based on CHX treatment sensitivity. **a** mRNA production for genes included in expression clusters affected during imbibition (Fig. 1e; *n* = 4 biological replicates at each time point; dashed line denotes threshold of new mRNA calling). **b** Diagram demonstrating a cycloheximide (CHX) experiment. Created in BioRender. Halale manjunath, V. (2026) https://BioRender.com/12q4y7v. **c** Volcano plots show CHX effects on new mRNA levels (the number of affected genes is indicated in the plots; DESeq2; *n* = 3; ERCC-based normalisation; non-transcribed genes normalisation; padj < 0.05; −log_10_(padj) capped at 50; |log_2_FC| > 1, capped at 5) during imbibition for 3 h and **d** 6 h. **e** Clustering of DEGs from (**d**) in the time-course imbibition data (Fig. 1; WGCNA with *z*-score normalised data). Identified clusters were overlapped with imbibition-affected genes (Fig. 1e), and similarly changed mRNAs were assigned to an extended cluster (containing genes with padj < 0.05 or |log_2_FC| < 1). The one-sided Fisher exact test was performed to determine the significance of overlap with the original clusters, with adjusted *p*-values shown on the cluster indicators; grey colour indicates a group of genes with no clear pattern of expression change. GO-term enrichment results are shown for representative biological process terms. **f** Changes in new mRNA levels between 6 h CHX- and mock-treated samples for genes included in expression clusters affected during imbibition (Fig. 1e; *n* = 3 biological replicates). **a** and **e** central line represents median; whiskers indicate a 1.5 interquartile range; outliers are marked with dots; letters denote statistical differences (<0.05) using one-way ANOVA with Tukey’s post hoc test. Source Data file contains exact *p*-values. **g** Model illustrating the influence of CHX on mRNA levels of genes belonging to distinct waves of mRNA upregulation.

Our analysis of total mRNA levels during imbibition suggested two waves of gene activation (Fig. 1e). Despite the widespread resumption of transcription during imbibition (Fig. 2c, d), the timing of new mRNA production revealed the same order. The genes in the early upregulated C1 cluster (Fig. 1e) exhibit the highest mRNA production across all time points, while for the second upregulation wave (C2), mRNA synthesis increases gradually (Fig. 3a). Given that we observe the early wave already at 1 h of imbibition, we hypothesised that it may be driven by factors inherited from the seed maturation stage. This hypothesis is supported by previous studies showing that translation inhibition with cycloheximide (CHX) did not affect the induction of some mRNAs in the first hours of imbibition^11^. Importantly, CHX inhibits translation of the DEX-induced reporter^47^, indicating that it can block translation early in seed imbibition (Supplementary Fig. 3a). To validate the contribution of inherited and de novo-generated proteins for new mRNA production, we examined total and new mRNA levels in scarified seeds imbibed in the presence of cycloheximide (Fig. 3b). Because cycloheximide treatment can globally impact mRNA stability^48^, we used ERCC spike-in normalisation to identify differentially expressed genes using DESeq2. At 3 h, CHX treatment had almost no impact on total mRNAs (Supplementary Fig. 3b and Supplementary Data 3), with little effect on total mRNA at 6 h (Supplementary Fig. 3c and Supplementary Data 3). Notably, CHX treatment did not impede global mRNA production compared with mock samples (Supplementary Fig. 3d and Supplementary Data 3), indicating that early transcription during imbibition is independent of de novo protein synthesis. These results show that during the initial hours of imbibition, Pol II transcription may primarily rely on transcriptional machinery and factors inherited from the seed maturation stage.

Surprisingly, CHX treatment increased new mRNA levels for 2013 genes at 3 h and 1208 genes at 6 h timepoint (Fig. 3c, d and Supplementary Data 3). This effect is consistent with earlier reports indicating that blocking translation may increase some mRNA levels during early imbibition^11^. In addition, 390 genes exhibited decreased new mRNA levels upon CHX treatment at 6 h (Fig. 3d). Importantly, these new mRNAs show high levels of expression at the 3 h time point but are further upregulated between 3 and 6 h of imbibition in mock-treated seeds, which is blocked upon CHX treatment (Supplementary Fig. 3e). This suggests that the block of translation elongation suppresses the gene expression shift that normally happens during early seed imbibition.

Our observation that inhibiting translation elongation can increase levels of certain newly transcribed mRNAs is surprising. To verify this, we examined the effects of puromycin (PUR), another translation elongation inhibitor^49^ and performed a 6 h CHX treatment in parallel as a control. The results from the new CHX treatment, both regarding total and newly synthesised mRNA levels, closely mirrored those of the initial experiment (Supplementary Fig. 3f–i), confirming the reproducibility of our findings. Importantly, puromycin treatment, like CHX, had minimal impact on total mRNA levels but increased levels of newly synthesised mRNA for 537 genes (Supplementary Fig. 3f–h). These genes exhibit significant overlap and similar GO terms with mRNAs upregulated by CHX treatment (Supplementary Fig. 3i and Supplementary Data 3). Finally, we performed RT-qPCR verification of 4-SU-labelled new mRNA enrichment in mock- and CHX-treated seeds, which, in most cases, confirmed the 3′RNA-seq results (Supplementary Fig. 3j). All these results indicate that blocking translation elongation raises the levels of newly synthesised mRNA for some genes, likely due to either enhanced Pol II transcription or stabilisation of these new mRNAs during treatment. The observed differences between cycloheximide, which shows up and downregulation of genes at 6 h, and puromycin, which shows only upregulation of genes at 6 h, could be attributed to their different modes of action^50^.

Translation inhibition was reported to stabilise mRNAs^48,50^. Accurate measurement of mRNA half-lives in seeds is challenging because the mRNA pool and transcriptional profiles change dramatically during seed imbibition. Therefore, we conducted a comparative analysis of total mRNA levels during seed imbibition at 1, 3 and 6 h with α-amanitin and with α-amanitin + CHX, normalising the data using external ERCC spike-ins (Supplementary Fig. 3k). α-amanitin mostly prevented mRNA upregulation during imbibition in both treatments (Supplementary Fig. 3l), consistent with earlier observations (Fig. 1). However, gene expression downregulation during imbibition was much less pronounced in CHX-treated/α-amanitin-treated seeds compared to α-amanitin treatment (Supplementary Fig. 3l, m). Notably, the newly synthesised mRNAs upregulated in CHX-treated seeds (Fig. 3d) tend to be among the stabilised total mRNAs in seeds exposed to CHX-treated/α-amanitin treatment (Supplementary Fig. 3n). This compares different fractions of mRNAs (total and newly synthesised), but may still suggest that new mRNA stabilisation upon translation blockage contributes to their upregulation. However, it is also possible that transcriptional blockade by α-amanitin is incomplete at the beginning of seed imbibition (Fig. 1e; see Supplemental Bioinformatic Methods), and the apparent stabilisation is at least partially due to enhanced transcription induced by CHX treatment. Given the specificity of our new mRNA isolation, as demonstrated using labelled and unlabelled spike-ins, it is unlikely that non-specific binding of stabilised total mRNAs is the cause of the observed increase in new mRNA signals (see also Supplemental Bioinformatic Methods).

To further characterise the genes whose new mRNA levels are affected by CHX treatment, we re-assigned them to clusters associated with imbibition using data from Fig. 1b. This revealed that genes with reduced transcription in response to CHX are predominantly induced later during imbibition (Fig. 3e), as we already observed (Supplementary Fig. 3e). Conversely, genes with increased new mRNA levels upon CHX treatment were overrepresented by those upregulated early during imbibition (Fig. 3e). Moreover, new mRNA fold changes between CHX- and mock-treated samples at 6 h confirmed the enhanced induction of the early-induced cluster and the suppression of the late-induced clusters (Fig. 3f). This observation reaffirms our initial suggestion that there are two waves of transcription upregulation during early imbibition and translation inhibition suppresses the transition to the second phase (Fig. 3g).

In summary, our observations indicate that early Pol II transcription reactivation during imbibition is not blocked by the two translation inhibitors used, suggesting that its first phase depends on protein factors inherited from seed maturation. This reliance may imply limited modulation by external conditions during the early transcription phase. However, the transition to the second phase of transcriptional activity does require active translation, suggesting that productive translation constitutes a checkpoint during seed germination transcriptional reactivation.

### The pattern of early transcription during imbibition is robust

**Fig. 4:**
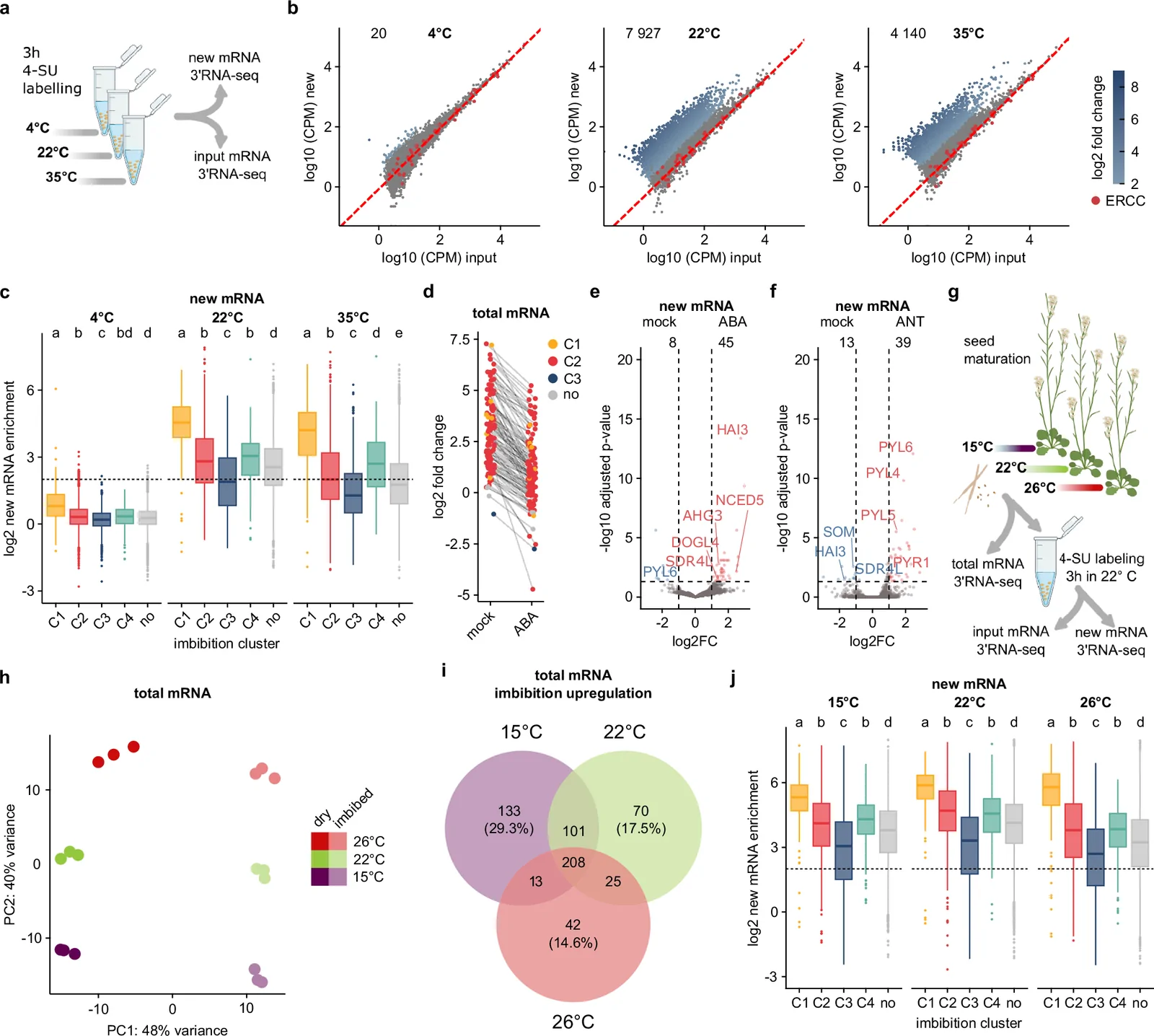
The early new mRNA production phase during seed imbibition is robust. **a** Diagram demonstrating an mRNA labelling experiment upon different temperatures of imbibition. Created in BioRender. Halale manjunath, V. (2026) https://BioRender.com/x8b25dr. **b** Plots comparing input and enriched mRNAs after 3 h 4-SU labelling at 4, 22 and 26 °C, with new mRNAs highlighted in blue and their number indicated in the plots (DESeq2; *n* = 4; ERCC-based normalisation; padj < 0.05; log_2_FC > 2; red line—the trend of unlabelled mRNA enrichment). **c** mRNA production across imbibition temperatures for genes included in expression clusters affected during the imbibition time course in Fig. 1e; *n* = 4 biological replicates for each temperature. **d** The plot compares gene expression fold changes of 6 h mock- or ABA-treated seeds to dry seeds for DEGs downregulated by ABA treatment (ABA vs mock; Supplementary Fig. 4g; *n* = 4 biological replicates). Colours indicate clusters affected during imbibition (Fig. 1e). Volcano plots show **e** ABA and **f** ANT treatment effects on new mRNA production after 6 h imbibition (the number of affected genes is indicated in the plots; DESeq2; *n* = 4; non-transcribed genes normalisation; padj < 0.05; |log_2_FC| > 1, capped at 3). Selected ABA-related genes are indicated. **g** Diagram demonstrating an mRNA labelling experiment for seeds matured at different temperatures. Created in BioRender. Halale manjunath, V. (2026) https://BioRender.com/80t68cf. **h** PCA plot summarising 3′RNA-seq results from dry and 3 h imbibed seeds matured at different temperatures (*n* = 3). **i** A Venn diagram showing genes with upregulation of expression during 3 h of imbibition for seeds matured at different temperatures (DESeq2; *n* = 3; padj < 0.05;|log_2_FC > 1 |). **j** mRNA production in seeds from different maturation temperatures for genes included in expression clusters affected during imbibition (Fig. 1e); *n* = 3 biological replicates for each temperature. C and J) Central line represents median; whiskers indicate a 1.5 interquartile range; outliers are marked with dots; dashed line denotes threshold of new mRNA calling; letters represent statistical differences (<0.05) using one-way ANOVA with Tukey’s post hoc test. Source Data file contains exact *p*-values.

Our results suggest that the initial wave of Pol II transcription activation is driven by regulators inherited from the seed maturation stage. We hypothesised that this early transcription phase may primarily respond to desiccation and rehydration-related stresses^51^, suggesting that imbibition conditions, while strongly affecting seed germination outcome, may have limited effects on early Pol II transcription activation patterns. To validate this hypothesis, we imbibed seeds at three temperatures (4, 22, 35 °C) for 3 h (Fig. 4a), as this time point marks the onset of widespread transcription (Fig. 2). These temperatures were selected as brief low-temperature imbibition breaks seed dormancy, while prolonged exposure to either high or low temperatures inhibits germination^52,53^.

The analysis of new mRNA production revealed weakly detectable transcriptional activity at 4 °C and a reduction at 35 °C compared to 22 °C (Fig. 4b, Supplementary Fig. 4a and Supplementary Data 4; DESeq2, ERCC spike-in normalisation). The low transcription at 4°C is possibly explained by slower imbibition and lower enzymatic activity. Importantly, a comparison of new mRNA production across the imbibition gene expression clusters (Figs. 1e and 3a) indicated that early activated genes (C1) exhibited the highest transcription levels at all temperatures, including at 4 °C (Fig. 4c). The overall similarity of transcription patterns across imbibition temperatures implies that the transcription of the early-induced gene cluster may represent a universal initial response of desiccated seeds to water uptake. Surprisingly, the limited new mRNA production at 4 °C contrasts with the upregulation of expression for a significant group of genes in total mRNA samples at this temperature (Supplementary Fig. 4b, c). This differs from groups of genes that are upregulated during imbibition at 22 °C, which exhibit significant new mRNA production (Figs. 3a and 4c) and show blockage of upregulation upon α-amanitin treatment (Fig. 1). Apparent upregulation of some mRNAs in the total fraction at 4 °C is most likely an outcome of slower downregulation of mRNAs whose levels drop during imbibition (Supplementary Fig. 4d; clusters C3 and C4) rather than results from the new transcription (Supplementary Fig. 4e). This observation indicates that during dynamic developmental gene expression shifts, such as early germination, the upregulation of mRNA levels observed in total RNA-seq is not always due to active transcription.

Given the lack of a temperature effect on the pattern of transcription upregulation, we investigated the impact of the ABA hormone, a well-established and specific germination inhibitor^54^. In our time-course experiment of total mRNA changes during imbibition (Fig. 1), we included samples treated with exogenous ABA for 6 h (Supplementary Fig. 4f). Differential gene expression analysis identified only a few dozen genes whose expression was significantly affected by ABA treatment compared to mock (padj < 0.05, |log₂FC| > 1) (Supplementary Fig. 4g and Supplementary Data 4). The genes whose expression is downregulated by ABA treatment largely overlap (86%, *n* = 104) with those induced at a later phase of transcription activation during standard imbibition (C2; Fig. 4d), suggesting that ABA hinders the induction of a subset of mRNAs.

In a follow-up experiment (Supplementary Fig. 4h), we sequenced new mRNAs from six-hour mock and ABA-treated seeds. As this experiment lacked externally applied ERCC spike-ins, we used a set of ERCC-like endogenous imbibition-non-transcribed mRNAs as an alternative in data normalisation (Supplementary Bioinformatic Methods, Supplementary Fig. 2n; Supplementary Data 2). ABA treatment did not globally alter early new mRNA production (Supplementary Fig. 4i), and the number of differentially produced mRNAs was low (Fig. 4e). However, there is a clear difference in new mRNA production for genes differentially expressed in total mRNA analysis, confirming ABA works at the transcriptional level (Supplementary Fig. 4j). This finding indicates that, although exogenous ABA inhibits later steps of germination (Supplementary Fig. 4k), its effect on transcription during early imbibition is mild. One reason for this may be high levels of endogenous ABA in early-imbibed seeds^55^, which could mask the effects of the exogenously applied hormone until endogenous ABA levels drop in mock-treated seeds. To test this, we performed imbibition in the presence of antabactin (ANT), an ABA receptor antagonist^27^. As expected, ANT suppresses the effects of exogenously applied ABA (Supplementary Fig. 4k). Similar to ABA treatment, ANT did not significantly affect global new mRNA production (Supplementary Fig. 4l; normalisation with imbibition-non-transcribed mRNAs), except for a few dozen mRNAs (Fig. 4f). Nonetheless, among the mRNAs affected by both treatments were key factors involved in ABA signalling, confirming the phenotypic effects of ABA and ANT (Supplementary Fig. 4k). These results suggest that modulating ABA and its signalling pathways have a modest influence on transcription during the initial hours of imbibition. This limited effect supports the hypothesis that during the first hours of imbibition, the main drivers of new transcription patterns are directly inherited from the seed maturation stage rather than influenced by imbibition conditions.

Another explanation for the conservation of gene transcription patterns during early imbibition may be that any viable Arabidopsis seed must always recover from the same stresses induced by desiccation and rehydration. If this hypothesis holds true, then seed maturation conditions should also have a limited impact on the general pattern of new mRNA production during early imbibition, as seed maturation concludes in a similar dry state. To investigate this, we used seeds matured at different temperatures (15, 22, 26 °C) (Fig. 4g), known to significantly impact dormancy and germination rates^21^. 3RNA-seq analysis of total mRNA in dry seeds revealed large differences due to maturation conditions (Fig. 4h and Supplementary Fig. 5a). For instance, seeds matured at 15°C exhibited elevated mRNA levels of germination repressors such as *DOG1*, *RDO5* and *SOM* ^56^ (Supplementary Data 5), aligning with low maturation temperatures leading to higher dormancy^22,23^. We observed that transcriptomic differences in dry seeds tend to persist during 3 h of imbibition under uniform conditions (Supplementary Fig. 5a). Importantly, genes that exhibit upregulated expression during imbibition show significant overlap (Fig. 4i), although the level of gene activation may vary among seeds matured at different temperatures (Supplementary Fig. 5a).

In agreement with similar activation of gene expression during imbibition, new mRNA production was unaffected on a genome-wide scale in seeds matured at various temperatures (Supplementary Fig. 5b; normalisation with imbibition-non-transcribed mRNAs). Furthermore, the pattern of new transcription across the C1–C4 gene expression clusters (Fig. 1) remained consistent for seeds matured at different temperatures (Fig. 4j), including the universal strong induction of the early C1 group. This supports the hypothesis that these genes contribute to early recovery from desiccation-associated stresses, which is independent of seed maturation temperatures. Interestingly, a relatively small number of genes with differential new mRNA production due to maturation conditions included many HSPs (15 °C; Supplementary Fig. 5c, d and Supplementary Data 5; see Supplemental Bioinformatic Methods for a detailed description of differential analysis of mRNA levels). This may suggest that differences in early transcription for seeds matured at various temperatures are mainly attributed to the shift in temperature from maturation to imbibition.

In conclusion, our findings suggest that an early gene activation wave is initiated during the first phases of imbibition. This initial transcriptional response is resilient to all analysed conditions, implying it is a part of the default developmental programme initiated during the rehydration stage.

### The transcription reactivation pattern is conserved in *Brassica napus*

**Fig. 5:**
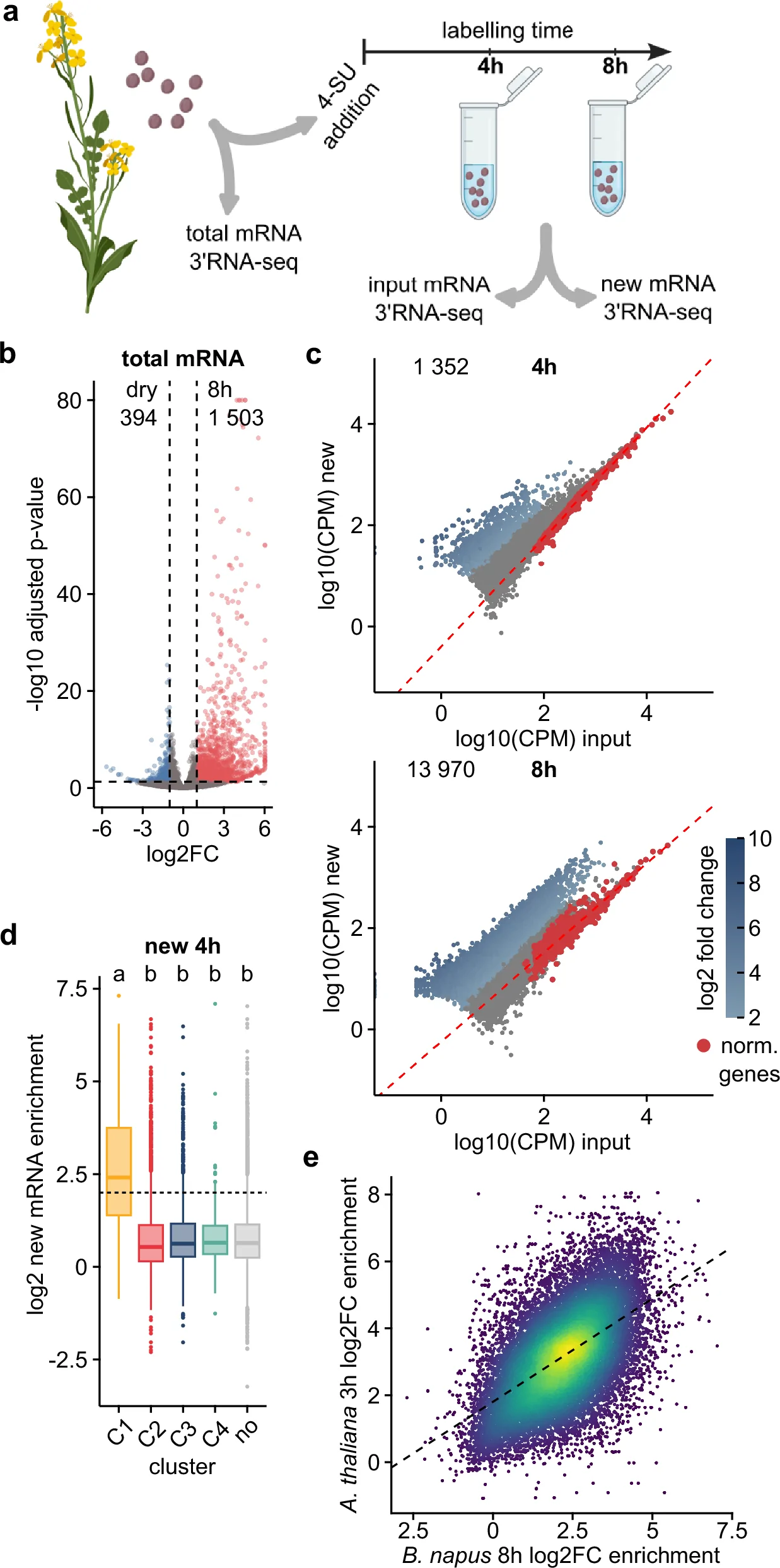
The pattern of new transcription is conserved during *B. napus* imbibition. **a** Diagram demonstrating labelling of new mRNAs in *B. napus* seeds. Created in BioRender. Halale manjunath, V. (2026) https://BioRender.com/d5hadc1. **b** A volcano plot illustrating changes in gene expression during *B. napus* seed imbibition, number of DEGs indicated (DESeq2; *n* = 4; padj < 0.05; −log_10_(padj) capped at 80; |log_2_FC| > 1, capped at 6). **c** Plots comparing input and enriched mRNAs after 4-SU labelling, with new mRNAs highlighted in blue and their number indicated in the plots (DESeq2; *n* = 4; downregulated genes normalisation—see text; padj < 0.05; log_2_FC > 2; red line—the trend of unlabelled mRNA enrichment). **d** New mRNA production during 4 h of imbibition for homologues of genes included in expression clusters affected during Arabidopsis imbibition (Fig. 1e; n = 4 biological replicates; central line represents median; whiskers denote a 1.5 interquartile range; outliers are marked with dots; dashed line denotes threshold of new mRNA calling; letters represent statistical differences (<0.05) using one-way ANOVA with Tukey’s post hoc test. Source Data file contains exact *p*-values. **e** Comparison of new mRNA production (bead enrichment) in *B. napus* (8 h) and Arabidopsis (3 h). Point density is illustrated with the Viridis scale; the line represents a trend.

We analysed mRNA production in scarified *Brassica napus* seeds at 4 and 8 h during imbibition (Fig. 5a). The total mRNA samples showed moderate transcriptomic changes compared to Arabidopsis (Fig. 1), with most genes displaying increased expression (Fig. 5b, Supplementary Fig. 6a, b and Supplementary Data 6), consistent with previous findings^57^. This suggests that transcriptomic changes in *B. napus* seeds occur more slowly during imbibition than in Arabidopsis. Notably, even among mRNAs upregulated at 8 h, we observed no enrichment of translation-related genes, a hallmark of the later imbibition phase (Supplementary Fig. 6b). The PCA map, including new mRNA and input samples, suggests that, similar to Arabidopsis (Fig. 2e), new mRNA transcriptomes precede total mRNA changes at later time points (Supplementary Fig. 6c). Next, we analysed the enrichment of new mRNAs relative to inputs and identified significantly enriched new mRNAs. As this experiment lacked ERCC spike-ins, we used a set of mRNAs with ERCC-like level changes as a normalisation control (see Supplemental Bioinformatic Methods for a detailed explanation of their identification). This revealed that new mRNA production was low at 4 h but significantly increased at 8 h (Fig. 5c, Supplementary Fig. 6d and Supplementary Data 6). As expected, genes with upregulated expression during imbibition demonstrated higher new mRNA levels (Supplementary Fig. 6e), confirming that our new mRNA identification method is applicable to rapeseeds. The analysis of the gene homologues from clusters C1–C4 (Fig. 1) indicated that the early upregulated cluster C1 remained the most highly transcribed at both 4 and 8 h (Fig. 5d and Supplementary Fig. 6f), while transcription in cluster C2 was not upregulated by 8 h imbibition. Additionally, the group of non-transcribed Arabidopsis genes is also largely not transcribed in *B. napus* (Supplementary Fig. 6g). Importantly, direct comparison of new mRNA enrichment clearly shows high similarity of new mRNA production during imbibition in both species (Fig. 5e and Supplementary Fig. 6h). This indicates that the pattern of transcription reactivation during imbibition is largely conserved between the two species.

### Pol II binding pattern in dry seeds has a limited effect on transcription reactivation

**Fig. 6:**
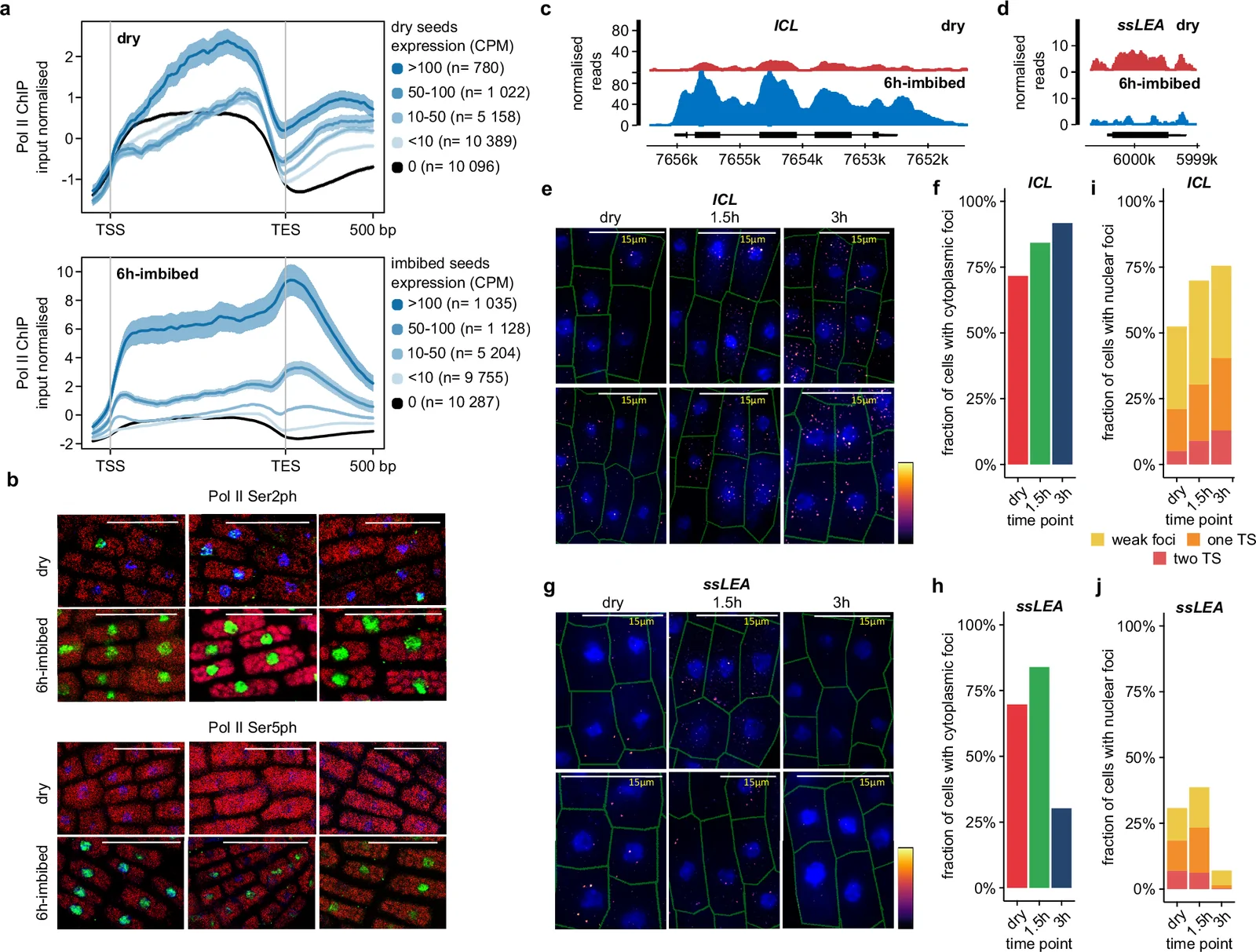
Pol II binding in dry seeds reflects seed maturation gene expression patterns. **a** Metaprofiles of Pol II along genes binned by mRNA expression for dry and 6 h imbibed seeds. ChIP-seq data (*n* = 3) were normalised to input, averaged and binned into 10 bp intervals (solid lines show means, and shaded areas indicate 95% confidence intervals; TSS refers to the transcription start site, and TES refers to the transcription end site). **b** Immunolocalisation results for phosphorylated Pol II (Ser2ph and Ser5ph) in embryo root tip cells (scale bar is 20 μm; blue—DAPI staining; red—cell autofluorescence, green—Alexa Fluor Plus 488). **c** Pol II ChIP-seq profiles of input-normalised reads in *ICL* and **d** *ssLEA* genes for dry and 6h-imbibed seeds. **e** *z*-stack max-projection images of *ICL* smFISH in the embryo root tip (blue -DAPI staining; arbitrary smFISH probes linear intensity scale is shown). **f** The proportion of cells with cytoplasmic foci for *ICL* probes. **g** *z*-stack max-projection images of *ssLEA* smFISH. **h** The proportion of cells with cytoplasmic foci for *ssLEA* probes. **i** Proportions of cells with TS for *ICL* and **j** *ssLEA* smFISH. TS is defined as having a stronger intensity than the median cellular foci (*ICL* − 3 and *ssLEA* − 1.5 times stronger; see “Methods”).

It has been shown that, although at low levels^2^, Pol II remains bound to chromatin in dry seeds^17,18^. The presence of Pol II could explain why imbibed seeds do not require new proteins for transcription reactivation (Fig. 3). We performed Pol II ChIP-seq analysis on both dry and 6-h imbibed seeds. First, we identified genes with significant Pol II binding (Supplementary Fig. 7a and Supplementary Data 7), which we confirmed with ChIP-qPCR (Supplementary Fig. 7b). The genes with Pol II peaks were likely under-detected due to technical challenges associated with ChIP analysis in seeds^4^. More importantly, gene body Pol II-enriched genes exhibit open promoter regions in ATAC-seq data from dry seeds^17^ (Supplementary Fig. 7c), confirming the specificity of ChIP-seq and consistent with their Pol II recruitment during seed maturation and imbibition. In general, Pol II binding to genes was low in dry seeds and proportional to mRNA levels (Fig. 6a). Although not surprising, this observation implies that in dry seeds, Pol II binding reflects the stored mRNA production during maturation^17^. This is also validated by GO-terms associated with Pol II peaks in dry seeds (Supplementary Fig. 7d). The observed pattern of Pol II binding in dry seeds is consistent with the genome-wide reactivation of transcription recapitulating seed maturation (Fig. 2).

Importantly, the Pol II binding metaprofile in imbibed seeds, but not in dry seeds (Fig. 6a), resembles that observed in seedlings^58^, showing a pronounced peak at the 3′ end of genes, which is characteristic of actively transcribed Arabidopsis genes^58^. Together with low levels of Pol II binding, this suggests that Pol II is inactivated and largely depleted from the DNA during seed desiccation. To verify this, we performed immunolocalisation of active Pol II complexes in different phosphorylation states of its large subunit’s C-terminal domain (Fig. 6b and Supplementary Fig. 7e). Pol II Ser5ph, indicative of transcription initiation^58^, was undetectable in dry seeds but became apparent after 6 h of imbibition. Similarly, Pol II Ser2ph, enriched in actively transcribed genes^58^, exhibited low levels in dry seeds but increased following imbibition (Fig. 6b and Supplementary Fig. 7e). These findings confirm the low potential activity of Pol II in dry seeds and the quick reactivation of Pol II transcription in 6 h imbibed seeds.

In agreement, the timing of gene expression activation during imbibition is related to detected Pol II levels in imbibed seeds, with genes activated earlier accumulating more Pol II. It is important to note that Pol II recruitment is delayed in ChIP experiments compared with newly synthesised mRNA analysis due to technical reasons. ChIP analysis requires large amounts of seed, which is not compatible with seed nicking used to enhance 4-SU uptake, and a damaged seed coat facilitates slightly faster water uptake, thereby desynchronising these two experiments. Ordered Pol II recruitment is evident when genes are grouped according to their mRNA upregulation timing (Supplementary Fig. 7f), the levels of conversion in total mRNA samples (Supplementary Fig. 7g), or by using clusters of expression changes during imbibition (C1–C4) (Supplementary Fig. 7h). However, in general, Pol II binding levels in dry seeds are not associated with gene expression upregulation during imbibition.

To confirm this conclusion, we chose two representative genes which showed Pol II binding in dry seeds but opposite transcriptional outcomes during imbibition, and performed single-molecule fluorescence in situ hybridisation (smFISH). This technique allows quantifying and localising mature mRNAs and partially transcribed molecules in embryonic root cells^59,60^. The first transcript, *ICL*, is upregulated during imbibition (Supplementary Fig. 8a) and produces new mRNAs (Supplementary Data 2), which is in line with its role in seed reserve mobilisation^61^. For the *ICL* gene, the Pol II peak was evident in both dry and imbibed seeds (Fig. 6c). The number of cytoplasmic foci corresponding to individual *ICL* mRNAs increased during imbibition (Fig. 6e and Supplementary Fig. 8b, c), along with a greater proportion of cells containing them (Fig. 6f). The second transcript, *ssLEA*, is downregulated during imbibition (Supplementary Fig. 8a), with a Pol II peak observed only in dry seeds (Fig. 6d) and no production of new mRNAs (Supplementary Data 2). The *ssLEA* gene is highly expressed during seed maturation and contributes to seed longevity^62^. In agreement with the conclusion that Pol II presence on the gene in the dry seeds is not predictive of transcription upregulation upon imbibition, the number of *ssLEA* foci decreased (Fig. 6g and Supplementary Fig. 8d, e), and the proportion of cells containing that mRNA was lower during imbibition (Fig. 6h). Moreover, both probe sets revealed prominent nuclear dots corresponding to mRNAs around transcription sites (TS)^60^, with the number of nuclear dots increased for *ICL* (Fig. 6i) and decreased for *ssLEA* (Fig. 6j) during imbibition in accordance with ChIP-seq results. The low number of nuclear foci observed with *ssLEA* probes already in dry seeds may align with its transcriptional shutdown during seed maturation^24^. Interestingly, the average intensity of TS remained unchanged between dry seeds and imbibition for both probe sets (Supplementary Fig. 8f–h), suggesting that transcriptional reactivation is regulated by the number of active TS (burst frequency) rather than the Pol II molecules loaded per TS (burst intensity)^60^. Taken together, smFISH results confirm that the binding of Pol II to the gene in dry seeds is not a factor deciding about its expression upregulation during imbibition.

## Discussion

Early studies in Arabidopsis demonstrated that even in seed coat-defective mutants, α-amanitin inhibits germination only at the radicle protrusion stage^10,12^. This led to the long-standing hypothesis that Pol II activity during early seed germination is largely unnecessary and that stored mRNA may initially support translation and germination^10,12,14,63^. On the other hand, numerous early experiments showed quick incorporation of radioactive nucleotides into RNA during imbibition of isolated embryos from multiple species^63^. Moreover, isolated embryos were responsive to transcription inhibitors^63,64^, which suggests that the inhibitors have limited penetrance through the seed coat^28^. In accordance, a smaller but more pleiotropic inhibitor—cordycepin can completely block germination in lettuce and Arabidopsis^10,12,28^. Nevertheless, changes in total mRNA levels upon early imbibition in the presence of cordycepin are relatively small^12^, which again may result from the limited penetrability of the seed coat. Alternatively, cordycepin during imbibition may affect other processes like mRNA polyadenylation^30^ or cellular signalling^65^.

In this study, we used scarified Arabidopsis seeds and observed nearly complete inhibition of germination following α-amanitin treatment (Fig. 1), indicating that Pol II transcription is essential for germination. RNA-seq analysis revealed that transcription inhibition results in mRNA level differences by the third hour of imbibition (Fig. 1). We also detected new mRNAs as early as one hour after imbibition, with widespread mRNA production occurring in subsequent time points (Fig. 2), suggesting an early and genome-wide reactivation of Pol II activity. Several thousand genes identified by us as transcribed during early imbibition may seem a large number, but ref. ^45^ identified more than 20,000 genes producing new transcripts in Arabidopsis seedlings. The resemblance between the total and the new mRNA fraction may also be expected. At this stage, the decision to germinate or delay germination and retain a seed-specific transcriptional program has not been made, so seed-characteristic transcription is logical. Notably, this new transcription included mRNAs downregulated during imbibition, and that effect could not be detected under standard analysis, where gene activity is inferred from total mRNA levels. Our approach consistently showed stronger gene expression changes in the new mRNA fraction than in analysis based on total mRNA levels (Fig. 3 and Supplementary Fig. 4). Importantly, any upregulation in total mRNA levels may result not only from increased transcription but also from slower downregulation, which can be distinguished by new mRNA labelling (Supplementary Fig. 4). These observations highlight the advantages of utilising new mRNA detection for studying dynamic processes such as seed imbibition and germination. It is possible that incorporating 4-SU into mRNA affects its metabolism, for example, by stabilising it. Although our pulse labelling and 5-EU data indicate that this is unlikely, we view the stabilisation of mRNA by 4-SU as an improvement in the method’s sensitivity rather than a significant flaw. As demonstrated in Supplementary Bioinformatic Methods and Supplementary Fig. 9, the reproducibility of new mRNA identification by enrichment is high. Although new mRNA calling is easier when external positive and negative spike-ins are applied, they can be replaced by a set of endogenous non-transcribed controls. In fact, due to the inherent imprecision in keeping the RNA-to-spike-in ratio, the usage of endogenous controls may increase reproducibility between samples.

The maturation of orthodox seeds ends with desiccation, which triggers specific pathways important for seed survival^1^. Prolonged storage also causes seed damage, primarily due to the accumulation of reactive oxygen species^66^. Various repair pathways activated during desiccation that enhance seed longevity remain functional during imbibition to support recovery from the dry state before advancing to germination^66^. Our findings support this notion, as the transcription of the seed maturation-expressed genes is continued during early imbibition. mRNAs stored at high levels in seeds encode proteins related to desiccation tolerance and longevity^14^, and perhaps, under favourable conditions, they are sufficient to complete the recovery phase, potentially reducing the need for new transcription. However, such ideal circumstances are rare in nature. Firstly, if seeds are stored for a prolonged period, a substantial portion of the mRNA pool may become damaged^66^, necessitating new transcription to replenish it. Secondly, environmental conditions during imbibition often prevent germination, resulting in imbibed seeds that are inhibited in their progression toward the germination state. Maintaining the expression of stress-responsive pathways is likely critical in such case. The recapitulation of the seed maturation transcriptional programme may also explain the limited impact of exogenous ABA and ABA signalling inhibitor during the early imbibition (Fig. 4). ABA-related regulation is crucial for achieving desiccation tolerance during seed maturation^67^. Our data suggest that this regulatory imprint likely persists when transcriptional activity resumes during early imbibition, and it does not depend on continuous ABA sensing, as demonstrated by antabactin treatment. The transcriptional resumption is likely facilitated by the chromatin configuration established during desiccation, including patterns of Pol II binding (Fig. 6), which preserves the expression profile of maturation genes. However, low Pol II phosphorylation in dry seeds indicates minimal transcriptional potential^58^, suggesting these complexes do not directly reactivate transcription. Instead, they likely serve as chromatin marks to help the recruitment of new Pol II complexes during imbibition.

A subset of genes associated with the recovery phase and hypoxia response are the first to be upregulated in imbibed seeds^38^ (Fig. 1), regardless of maturation and germination conditions (Figs. 2–4). This is likely due to the conserved seed maturation process, which can vary with temperature (Fig. 4) but invariably concludes with seed desiccation, thereby leading to a similar pattern of transcription reactivation during imbibition. For example, limited oxygen diffusion through the seed coat and dense cytoplasm always leads to hypoxic conditions during imbibition^51^. A complementary explanation for the uniformity of transcription upregulation might be that seeds are unable to sense and adjust to surrounding conditions at the early imbibition stage. Consequently, the early-transcribed genes may have been evolutionarily selected as the most robust set, for “unsupervised” reinitiation of Pol II activity across diverse imbibition scenarios. Conversely, some genes remain non-transcribed during imbibition despite being highly expressed during maturation. These include genes essential for preparing for desiccation, accumulating storage materials, or enhancing dormancy (Supplementary Data 2). It appears that these genes were deemed unnecessary during early imbibition, perhaps because their expression could delay seed germination in favourable conditions.

The transition towards germination is characterised by an upregulation of genes involved in ribosome biogenesis and translation^35^ (Fig. 3). Cycloheximide-treated seeds stop changing their transcriptomes at this stage (Fig. 3), suggesting the decision to germinate depends on the seed’s ability to recover from desiccation and rehydration-induced stresses^51^. The differential response of biphasic induction to translation inhibition was previously shown for inflammation-induced genes^68^, suggesting a similar mechanism. Notably, in dormant seeds, transcriptional changes are likely to pause early, as transcriptomic divergence between dormant and non-dormant seeds becomes apparent only after initial imbibition^69^. The germination-related transcription supports the resumption of cellular activities and the expansion of cells during germination. However, the shift to this highly active metabolic phase may be reversed, as evidenced during imbibition leading to secondary dormancy induction, wherein genes related to translation are initially activated but subsequently shut down^35^. In summary, our results show that while stored protein factors support the reinitiation of seed metabolism during imbibition, de novo transcription is essential for the germination process.

## Methods

### Plant growth, seed assays and treatments

The *Arabidopsis thaliana* Col-0 ecotype and *Brassica napus* Bazyl cultivar were used in this study. All biological replicates were collected as distinct samples. Plants were grown in a greenhouse under a long day-night cycle at 22 °C. Seeds from 8 to 12 plants were combined to create a single biological replicate. To analyse Arabidopsis seeds that matured at different temperatures, plants were grown in a Percival growth chamber until the bolting stage at 22 °C long-day photoperiod (LD) and transferred to 15 or 26 °C LD for flowering and seed maturation. Seed imbibition was performed in darkness at 22 °C (standard conditions) or 4 and 35 °C (stress conditions).

We performed seed scarification to facilitate the rapid uptake of various drugs during imbibition. Arabidopsis seeds were immobilised on a microscopic slide covered with double-sided tape, and the seed coat was nicked under a dissecting microscope using an insulin needle. For *B. napus*, the seed coat was locally abraded using a grindstone. Seeds were imbibed in 250 μM α-amanitin (Merck; prepared from a 1 mM stock solution in water), 2 mM 4-thiouridine (4-SU) (Merck; from a 50 mM stock solution in 6% methanol), 2 mM 5-ethynyluridine (5-EU; Click-iT Nascent RNA Capture Kit, Thermo Fisher Scientific; 100 mM water solution), 1 mM cordycepin (Merck; prepared from 5 mg/ml water solution), 3 μM ABA (Merck; from a 1 mM stock solution in ethanol), 100 μM antabactin (Kerafast; from a 50 mM stock solution in DMSO), 4 mM puromycin (BioShop; from 50 mg/ml water solution), 500 μM cycloheximide (Merck; from a 100 mg/ml stock solution in ethanol) and 25 μM dexamethasone (DEX) as indicated. Corresponding concentrations of drug solvents were used as mock.

For short 4-thiouridine labelling, 100 mg of intact seeds were first imbibed for 2.5 h or 5.5 h, then squeezed between two microscopic glass slides, yielding a mixture of seed coats and both intact and fragmented embryos. Such a mixture was incubated in ½ MS medium supplemented with 4-SU for 0.5 h.

Germination assays were conducted on blue paper (HoffmanMfg) with a layer of water-soaked fabric underneath, placed in sealed plates under long-day conditions at 22 °C. Tetrazolium red (Merck) staining was carried out by overnight imbibition in a 1% solution, followed by transferring the seeds onto plant agar plates for photographic documentation. Seed germination in the presence of inhibitors, ABA or antabactin, was conducted by soaking in solutions for 8 h (250 μM α-amanitin, 100 μM flavopiridol; MedChemExpress, from 10 mM stock solution in DMSO, 1 mM cordycepin, 2 μM ABA, 100 μM antabactin). Afterwards, the seeds were placed on a thin layer of 0.5% plant agar containing the tested substances on microscopic glass and maintained in a high-humidity chamber under long-day conditions at 22 °C.

### Labelled spike-ins synthesis

Templates for in vitro transcription were a kind gift of Prof. Andrzej Dziembowski. They were obtained by PCR of the *E. coli LacZ* gene (different fragments of around 400 bp; templates A–G) or the synthetic construct containing the UTRs of the Moderna vaccine (template H). Forward primers contained the T7 promoter, and reverse primers were extended with the template for a poly(A) tail. PCR products were purified using AmpureXP beads (Beckman) and used for in vitro transcription with the TranscriptAid T7 High Yield Transcription Kit (Thermo Fisher Scientific). Two versions of the transcripts were prepared for each template: labelled (transcription with 7.5 mM UTP and 2.5 mM 4-sUTP; Jena Bioscience) and unlabelled (transcription with 10 mM UTP). For template F, we also prepared a fully labelled transcript (transcription with 10 mM 4-sUTP). Labelled and unlabelled transcripts were diluted, mixed at different ratios (1–20%) and used as spike-ins during enrichment of endogenous 4-SU labelled transcripts.

### RNA isolation, labelled RNA recovery and conversion

Seed RNA was isolated according to a standard seed protocol^70^ and treated with Turbo DNase (ThermoFisher). Reverse transcription was conducted using SuperScript III (ThermoFisher) and custom barcoded oligo(dT) primers for library preparation^35^ or a mixture of random and oligo(dT) primers for RT-qPCR analysis. In the RT-qPCR analysis, cDNA was diluted tenfold and qPCR was performed using SYBR Green I Master in a LightCycler 480 Real-time system (Roche). RT-qPCR results were normalised to *UBC21* (*AT5G25760*) or *ACT7* (*AT5G09810*) mRNA. The sequences of all primers are provided in Supplementary Table 1.

4-thiouridine (4-SU) labelled RNAs were purified using established protocols^40,42^ with slight modifications. Biotinylation was performed for 10–25 μg of freshly isolated RNA in a 200 μl reaction mixture (10 mM Tris pH 7.5; 1 mM EDTA; 0.2 mg/ml EZ-Link Biotin-HPDP (ThermoFisher)) for 1.5 h at 22 °C. In some experiments, 1 μl of a tenfold diluted ERCC RNA Spike-In Mix (Thermo Fisher) and in vitro-prepared spike-ins were added to the RNA before biotinylation reactions. Unbound Biotin-HPDP was removed by chloroform/isoamyl alcohol (24:1) extraction. Subsequently, a 1/10 volume of 5 M NaCl and an equal volume of isopropanol were added, and RNA was precipitated by centrifugation at 20,000 × *g* for 20 min. The pellet was washed with 1 ml of 80% ethanol and, after drying, resuspended in 25 μl of RNase-free water. Biotinylated RNA was purified using 50 μl of streptavidin C1 Dynabeads (ThermoFisher). The beads were washed in binding buffer (0.2 M NaCl; 0.1 M sodium phosphate pH 6.8; 25 mM MgCl_2_; 0.4% Tween), blocked in the binding buffer with 1 μg/μl glycogen for 10 min, and washed again in binding buffer. RNA samples were denatured at 65 °C for 5 min, followed by 5 min on ice. Then, 25 μl of 2× binding buffer and 50 μl of washed streptavidin beads were added, mixed and incubated at 22 °C with gentle shaking for 20 min. The beads were washed three times with 0.5 ml of washing buffer (100 mM Tris-HCl pH 7.5; 10 mM EDTA; 1 M NaCl; 0.1% Tween20) at 65 °C, followed by three washes at room temperature. Biotinylated RNA was eluted from the beads by two incubations with 50 μl of 200 mM DTT. RNA from the combined eluates was purified using RNA Clean & Concentrator-5 columns (Zymo), following the protocol for long RNA purification with DNase treatment. The entire volume of the sample was used for reverse transcription.

The conversion of 4-SU to cytidine (C) was carried out according to published protocols^41,71^, with minor modifications. Streptavidin-enriched or total labelled RNA was combined with a reaction mixture (600 mM 2,2,2-trifluoroethylamine (TFEA); 1 mM EDTA; 100 mM sodium acetate pH 5.2). Following this, meta-chloroperoxybenzoic acid (mCPBA) was added to a concentration of 10 mM. The reaction was incubated at 45 °C for 1 h, and subsequently, RNA was purified using an equal volume of RNAClean beads (Beckman) according to the manufacturer’s protocol. The RNA was eluted with 18 μl of water and mixed with 2 μl of 10× reducing mix (100 mM Tris-HCl pH 7.5; 100 mM DTT; 1 M NaCl; 10 mM EDTA). The reaction was incubated at 37 °C for 30 min. RNA was again purified using an equal volume of RNAClean beads (Beckman) following the manufacturer’s protocol, with an elution volume of 12 μl. This RNA was used for reverse transcription and the 3′RNA-seq library preparation or amplification of the *HSP17.6II* mRNA fragment analysed by Sanger sequencing.

For enrichment of 5-EU-labelled RNAs, we used the Click-iT Nascent RNA Capture Kit (Thermo Fisher Scientific). We started with 5 μg of total RNA and reacted it with 2.5 μl of 10 mM biotin azide. Biotinylated RNAs were purified using 50 μl of streptavidin beads, with reverse transcription performed on bead-bound RNAs in a 50 μl reaction with an oligo(dT)/random primers mix and SuperScript III enzyme. In parallel, reverse transcription of 0.5 μg input was performed. Both reactions were diluted to the same volume and used for qPCR analysis.

### 3′RNA-seq libraries

3′RNA-seq libraries were prepared following previously established protocols^35,72,73^. In brief, reverse transcription reactions were performed using custom barcoded oligo(dT) primers (TCGTCGGCAGCGTCAGATGTGTATAAGAGACAGNNNNNNNNNXXXXXXVVVVVTTTTTTTTTTTTTTTTTTTTTTTTTTTTTTVN with a Tn5-A sequence and X as barcode nucleotides). For each library, a fraction of the reverse transcription reactions with different barcodes was pooled. The volume used for pooling was determined by the number of libraries pooled, aiming to achieve a total RNA amount ranging from 1 to 1.5 μg in the pooled sample. For reverse transcription of enriched 4-SU-labelled transcripts, the entire reaction volume was pooled. Pooled samples were purified and concentrated using 1.5 volumes of RNAClean beads (Beckman) according to the manufacturer’s protocol and eluted with 16 μl of water. Next, 15 μl of the recovered sample was mixed with 15 μl of a second-strand synthesis mix containing 3 μl NEBNext Second Strand Synthesis (dNTP-free) Reaction Buffer (NEB), 3 μl RNaseH (NEB), 1.5 μl *E. coli* DNA ligase I (NEB), 7 μl *E. coli* DNA polymerase (NEB) and 0.5 μl of 0.4 μM dNTPs, and the mixture was incubated overnight at 16 °C. The reactions were purified using 1.5 volumes of RNAClean beads (Beckman) according to the manufacturer’s protocol and eluted with 4 μl of water. The Tn5 enzyme was overexpressed and purified^73^, loaded with the Tn5-B duplex, and diluted fivefold. Next, 3 μl of the recovered dsDNA sample was used for tagmentation, along with 3 μl of the diluted Tn5 enzyme and 6 μl of freshly prepared 2× buffer (20 mM Tris-HCl pH 7.5; 20 mM MgCl_2_; 50% DMF). Reactions were mixed by pipetting and incubated for 7 min at 55 °C, followed by 5 min at 80 °C. Water was added up to 36 μl, and samples were purified with 0.8 volumes of RNAClean beads and eluted with 21 μl of water. For Illumina indexing PCR, 20 μl of the recovered sample was used in 50 μl reactions with Q5 Hot Start High Fidelity 2× PCR Master Mix (NEB) and a final primer concentration of 0.5 μM. To prevent PCR over-cycling, the number of cycles was estimated as detailed in ref. ^74^. Libraries were sequenced on the Illumina system using paired-end sequencing, with R1 containing the barcode and Unique Molecular Identifier (UMI) and R2 containing the mRNA sequence.

### ChIP-seq and ChIP-qPCR

250 mg of dry and 6-h-imbibed seeds were ground in liquid nitrogen. The resulting powder was combined with 20 ml of crosslinking buffer (0.1 M sucrose; 0.1 M NaCl; 20 mM HEPES pH 7.0; 1% formaldehyde) in 50 ml tubes and gently mixed in the cold room for 10 min. Subsequently, 1.25 ml of 2 M glycine was added to each sample and mixed gently in the cold room for 5 min. The samples were then centrifuged at 1800 × *g* for 15 min at 4 °C, after which the supernatant was removed, and 20 ml of Honda buffer (0.44 M sucrose; 1.25% Ficoll; 2.5% Dextran 40; 25 mM HEPES pH 7.0; 10 mM MgCl_2_; 0.5% Triton ×-100; 0.5 mM PMSF; 10 mM DTT; 1× cOmplete EDTA-free Protease Inhibitor Cocktail (Roche)) was added to the pellet, which was then resuspended by mixing with a spatula. The samples were mixed gently in the cold room for 10 min and filtered through two Miracloth layers. They were then centrifuged at 1800 × *g* for 15 min at 4 °C; the supernatant was removed, and the pellet was resuspended in 500 µl of fresh NLB buffer (50 mM Tris-HCl pH 8.0; 10 mM EDTA; 1% SDS; 1× cOmplete EDTA-free Protease Inhibitor Cocktail (Roche)), after which the samples were incubated on ice for 30 min. Next, 500 µl of ChIP dilution buffer I (16.7 mM Tris-HCl pH 8.0; 167 mM NaCl; 1.2 mM EDTA) was added to the samples. The samples were sonicated using a Bioruptor (Diagenode) with internal cooling, employing 30 s ON/30 s OFF (High) settings for 20 cycles with 5-min breaks for cooling on ice after every 5 cycles. Successful sonication was verified by comparing a portion of the sample taken before and after sonication. Briefly, one volume of 10% Chelex was added to the samples, which were then incubated for 10 min at 95 °C and cooled to 37 °C. Next, 1 μl of RNaseA (10 mg/ml) was added, followed by a 15-min incubation. The samples were then heated to 55 °C, and 1 μl of proteinase K (20 mg/ml) was added, followed by an additional 15-min incubation. The samples were purified using phenol:chloroform extraction and then subjected to isopropanol precipitation. The pellet was resuspended in 14 μl of water, and the samples were resolved on an agarose gel.

Following successful sonication, the samples were centrifuged for 5 min at 20,000 × *g* and 4 °C, with the supernatants being carefully transferred to new tubes to avoid contamination from the upper layer. 60 μl of each sample was retained as input controls. Immunoprecipitation was conducted in high-recovery tubes using 400 µl of chromatin, to which 1600 µl of ChIP dilution buffer II (16.7 mM Tris-HCl pH 8.0; 167 mM NaCl; 1.2 mM EDTA; 1.1% Triton ×-100×; 0.6 mM PMSF; 1.2× cOmplete EDTA-free Protease Inhibitor Cocktail (Roche)) was added. The IP was performed with 2.5 µg of anti-RPB1 antibody (Agrisera AS11 1804; lot 2003), and the samples were incubated in the cold room with gentle rotation. The next day, 15 µl of ChIP dilution buffer II-washed Dynabeads Protein G (ThermoFisher) were added to the samples and incubated in the cold room with gentle rotation for 3 h. The supernatant was discarded, and the beads were washed twice with low salt buffer (150 mM NaCl; 0.1% SDS; 1% Triton ×-100; 2 mM EDTA; 20 mM Tris-HCl pH 8.0), once with high salt buffer (500 mM NaCl; 0.1% SDS; 1% Triton ×-100; 2 mM EDTA; 20 mM Tris-HCl pH 8.0), and twice with TE buffer.

For ChIP-seq, antibody complexes were eluted by adding 100 µl of elution buffer (1% SDS; 0.1 M NaHCO_3_) to the beads and incubating at 65 °C for 15 min. The supernatant was collected, and the elution was repeated with a fresh 100 µl of elution buffer. The supernatants were combined, mixed with 8 µl of 5 M NaCl, and incubated overnight at 65 °C. Input samples were processed similarly. To the input samples, 100 µl of TE buffer, 7.1 µl of 5 M NaCl and 17.4 µl of 10% SDS were added, followed by an overnight incubation at 65 °C. The following day, 5 µl of 0.5 M EDTA, 8 µl of 1 M Tris-HCl pH 6.5 and 2 µl of 20 mg/ml proteinase K were added to all samples, which were then incubated for 3 h at 55 °C. DNA was recovered using ChIP DNA Clean & Concentrator columns (Zymo), and libraries were prepared using the NEBNext Ultra DNA Library Prep Kit for Illumina.

For qPCR analysis, antibody complexes from beads and input samples were purified by adding 100 µl of 10% Chelex. The samples were mixed and incubated at 95 °C for 10 min, followed by adding 2 µl of 20 mg/ml proteinase K and incubation for 30 min at 55 °C. Proteinase K was inactivated by incubation at 95 °C for 5 min, and the samples were centrifuged for 5 min at 20,000 × *g*. 75 µl of supernatant was transferred to new tubes. Magnetic and Chelex beads were mixed with 75 µl of TE, centrifuged for 5 min at 20,000 × *g*, and the supernatants were combined. qPCR was performed with SYBR Green I Master in a LightCycler480 Real-time system (Roche). ChIP-qPCR results were normalised to the primers located at the second exon of the *TUB2* (*AT5G62690*) gene. The sequences of all primers are provided in Supplementary Table 1.

### Bioinformatic analysis of 3′RNA-seq

For 3′ RNA-seq, reads R1 and R2 were processed separately. Our oligo(dT) primers consist of two segments of the UMI separated by a barcode sequence. The read R1 fastq file was transformed using the command: *awk ‘NR%2* = = *1 {print $0} NR%2* = = *0 {print substr($1,16,5) substr($1,1,15)}’*. Read R2 was trimmed to remove potential contamination with the poly(A) tail using BRBseqTools^72^ (v 1.6) Trim with parameters *-polyA 10 -minLength 30*. The trimmed reads were then mapped using STAR^75^ (v 2.7.8a) to the TAIR 10 genome version and Araport11 genome annotation (or AST_PRJEB5043_v1 genome assembly for *B. napus*) with parameters *–sjdbOverhang 99 –outSAMtype BAM SortedByCoordinate –outFilterMultimapNmax 1*. For mapping of libraries containing ERCC spike-ins, their sequences were added to the Arabidopsis genome. Subsequently, the count matrix for each library and each gene was obtained using BRBseqTools^72^ (v 1.6) CreateDGEMatrix with parameters *-p UB -UMI 14 -s yes*, employing Araport11 genome annotation and a list of used barcodes in oligo(dT) primers. This generated count tables are available in Gene Expression Omnibus under accession number GSE297302. Count matrices were combined and used for differential expression analysis with DESeq2^76^. 4-SU conversion to C was analysed using SlamDunk^77^. GO term analysis was conducted using gprofiler2^78^, and we used the WGCNA^79^ R package for gene expression clustering. Data normalisation and additional control experiments are explained in Supplementary Bioinformatic Methods and shown in Fig. S9. A detailed description of the data analysis is provided on the GitHub website (https://github.com/mk1859/seed_transcription). The number of sequenced reads, as well as the number of deduplicated reads assigned to genes, is provided in Supplementary Table 2.

### Bioinformatic analysis of ChIP-seq

For ChIP-seq, adapter sequences were trimmed from reads using cutadapt^80^ (v 1.18) with the parameters: *-a AGATCGGAAGAGCACACGTCTGAACTCCAGTCA -A AGATCGGAAGAGCGTCGTGTAGGGAAAGAGTGT -e 0.2 –minimum-length* = *30*. Next, reads were mapped using bowtie2^81^ (v 2.3.5.1) with parameters: *–no-unal –local*. The mapped reads were then converted to BAM files and sorted using samtools^82^ (v 1.13). Mapped reads were initially filtered with sambamba^83^ (v 0.8.0) using the parameter *-F “[XS] == null and mapping_quality ≥ 10”*, followed by the removal of duplicated reads with gatk^84^ MarkDuplicates (v 4.2.6.1). Normalised bigwig tracks for each library and merged biological replicates were prepared using bedtools^85^ genomecov (v 2.30.0) and bedGraphToBigWig^86^ (v 4). Bigwig tracks normalised for the input were generated using bigwigCompare^87^ (v 3.5.1) with parameters *–binSize 1 –outFileFormat bigwig –skipZeroOverZero --operation subtract*. Metaprofiles were created using the seqplots^88^ R package. Peaks were called using macs2^89^ callpeak (v 2.2.7.1) with the *broad* option. Peaks identified in at least two biological replicates were used to find overlapping protein-coding genes. Results of these analyses are available in Gene Expression Omnibus under accession number GSE297302.

### smFISH and immunolocalisation of Pol II

The smFISH method was conducted^60^ for embryonic root tips isolated from dry, 1.5 and 3 h imbibed seeds. The set of smFISH probes labelled with the Quasar670 fluorophore was designed for *ssLEA* and *ICL* (Supplementary Table 3) using Stellaris Probe Designer (v 4.2; Biosearch Technologies). For smFISH image analyses, we used PartSeg^90^ and developed a plug-in ref. ^60^. All steps of picture pre-processing and analysis were described earlier^60^. To identify transcription sites (TS), we eliminated the two brightest nuclear foci per cell, as up to two foci can correspond to TS in diploid cells. We calculated the median fluorescence intensity from the remaining foci, approximating a single mRNA molecule intensity. Next, we calculated the fold-change intensity relative to this median value for all foci in the nucleus and nuclear periphery. The threshold for TS identification (3 times for ICL and 1.5 times for ssLEA) was manually adjusted to ensure that 90% of cells have up to two TS. For cells containing more than two foci exhibiting a fold-change above the threshold, we regarded the two brightest foci as the active TS. Processed microscopy data and a detailed description of their analysis are provided on the GitHub webpage (https://github.com/mk1859/seed_transcription).

For immunolocalisation, embryonic root tips were isolated from dry and 6 h imbibed seeds. The tips were placed on microscopic slides and fixed in 4% formaldehyde. The slides were snap-frozen in liquid nitrogen and then washed with 70% ethanol, followed by treatment with absolute methanol for 2 h. Next, they were washed three times with PBS buffer and once with citrate buffer pH 4.8 (5 min each) and treated with a 2% cellulase (MASEC0BAC—Merck) and 3% pectinase (P4716—Merck) enzyme solution in citrate buffer for 30 min at 35 °C. After washing with citrate buffer three times, the slides were incubated with 0.15% Triton in PBS for 10 min and washed three times with PBS. Finally, the slides were incubated overnight at 7 °C with primary antibodies diluted 1:100 (PBS pH 7.2 with 0.02% acBSA). We used Mouse anti-Phospho RNA polymerase II CTD repeat YSPTSPS antibody pS5 (ab5408) or Rabbit anti-Phospho RNA Polymerase II CTD repeat YSTPSTS antibody pS2 (ab5095) from Abcam. The following day, the slides were washed with PBS five times for three minutes each. They were then incubated with secondary antibodies: Goat anti-Rabbit IgG (H + L) Alexa Fluor Plus 488 or Goat anti-Mouse IgG (H + L) Alexa Fluor Plus 488 (ThermoFisher A-11008 and A-11001) at a dilution of 1:100 (PBS pH 7.2 with 0.01% acBSA) for three hours at 35 °C. After this, the slides were washed five times with PBS for three minutes. The slides were stained with DAPI. Images were captured with a Leica SP8 confocal microscope and with an optimised pinhole, long exposure time (200 kHz) using a 63× magnification (numerical aperture, 1.4) Plan Apochromat DIC H oil immersion lens. Images were collected sequentially. To minimise bleedthrough between fluorescence channels, the laser was set to low power (0.3% of maximum power) and sequential image captures were performed. For bleedthrough analysis and control experiments, the Leica SP8 software was used. Image acquisition was performed using constant parameters (laser power, detector gain, emission band and resolution).

### Western blot

Transgenic seeds expressing 3xmCherry under a dexamethasone-inducible promoter (Doumane et al., 2021) were imbibed in water (mock) or water + dexamethasone (25 µM) or water + dexamethasone + cycloheximide (150 µg/ml) for 6 h. Treated seeds were homogenised in liquid nitrogen, and total protein was isolated using the protein extraction buffer (50 mM Tris-HCl (pH 7.5), 150 mM NaCl, 1% Triton ×-100, 10% glycerol, 2 mM EDTA, 1 mM DTT, 1 mM PMSF and protease inhibitor cocktail). The concentration of protein was determined using the Bradford assay, and 70 µg of total protein was resolved on an 8.5% SDS-PAGE gel. Proteins were transferred onto PVDF membrane (Amersham Hybond, 10600023) using a wet transfer system at 80 V for 2 h at 4 °C (cold room). Following transfer, the membrane was blocked with 5% (w/v) skim milk in TBST for 30 min at room temperature. The membrane was then incubated overnight at 4 °C with anti-mCherry antibody (Abcam, ab167453, dilution 1:2000 in 5% skim milk). After washing with TBST, the membrane was incubated with goat anti-rabbit HRP-conjugated secondary antibody (Agrisera, AS09602, diluted 1:10,000 in 5% skim milk) for 1 h at room temperature. Following washes, protein bands were detected using Radiance ECL substrate (Azure Biosystems, AC2204) and visualised using an Azure 400 Imaging system.

### Statistics and reproducibility

All statistical analyses were performed using the R environment, and code is available on GitHub [https://github.com/mk1859/seed_transcription]. Libraries with too few reads, caused by technical issues, were omitted from the analysis. That is described on GitHub [https://github.com/mk1859/seed_transcription] in the step-by-step data analysis description. Sample sizes are mentioned in figure legends and/or displayed in plots as individual data points. Sample sizes were not pre-specified. Information regarding the statistical test applied to each dataset is mentioned in the corresponding figure legend. Significant differences were accepted at *p* < 0.05. The experiments were not randomised. The Investigators were not blinded to allocation during experiments and outcome assessment.

### Declaration of generative AI and AI-assisted technologies in the writing process

During the preparation of this work, the authors used Grammarly to improve the readability and language of the manuscript. After using this tool, the authors reviewed and edited the content as needed and take full responsibility for the content of the published article.

### Reporting summary

Further information on research design is available in the Nature Portfolio Reporting Summary linked to this article.

## Supplementary information

Supplementary Information

Supplementary Data 1

Supplementary Data 2

Supplementary Data 3

Supplementary Data 4

Supplementary Data 5

Supplementary Data 6

Supplementary Data 7

Reporting Summary

Transparent Peer Review file

## Source data

Source data

## Data Availability

Raw data of RNA-seq and ChIP-seq are deposited in the NCBI Gene Expression Omnibus under accession number GSE297302. In the manuscript, we used published data from ATAC-seq GSE250331. Processed microscopy data and the description of high-throughput sequencing analysis using R are available on GitHub [https://github.com/mk1859/seed_transcription]. Source Data is provided with this paper. Source data are provided with this paper.
