# Supplementary material for: Transcriptional activity during seed imbibition is conserved, robust and required for germination
Source: Nat Commun. 2026 Aug 19;17:9916. doi: 10.1038/s41467-026-76795-8 (PMC13582861; doi:10.1038/s41467-026-76795-8)
Supplement: Supplementary file 1 [file 41467_2026_76795_MOESM10_ESM.pdf]

nature portfolio

Peer Review File

Transcriptional activity during seed imbibition is conserved, robust and required for germination

Corresponding Author: Dr Szymon Swiezewski

This file contains all reviewer reports in order by version, followed by all author rebuttals in order by version.

Version 0:

Reviewer comments:

Reviewer #1

(Remarks to the Author)
This is a significant and well-executed study that fundamentally challenges the long-standing paradigm that stored mRNAs alone suffice for early seed germination, demonstrating instead that early and widespread Pol II transcription is essential. The work is rigorous, employs multiple complementary techniques, and provides compelling evidence across two species. The findings substantially advance our understanding of seed biology. However, certain descriptions in the manuscript lack clarity, and some interpretations of the data could benefit from more rigorous framing. These aspects warrant further refinement to enhance precision.

Major Concerns,
1. The most significant concern is the authors' lack of transparency in describing the data processing and analytical methods. Although the manuscript states that data and analysis code have been deposited in public databases or websites, the absence of accessible links prevents independent verification of the results' reliability. As noted by the authors: "A detailed description of the data analysis is provided on the GitHub website (to be provided upon publication)."
This issue is further illustrated by the inconsistent application of differential expression thresholds in the DESeq2 analyses. The reported $|log_2FC|$ cutoffs vary arbitrarily—ranging from >0 to >1.5 to >2—across different sections of the manuscript, leading to considerable confusion. Most critically, the methodology for data normalization—a fundamental step in the analysis—is inadequately described, undermining confidence in the analytical rigor.
2. In the third section of the Results, the authors treated seeds with the translation inhibitor CHX. They report minimal differences in total RNA levels between CHX- and mock-treated samples at 3h and 6h, concluding that early transcription relies primarily on transcriptional machinery pre-bound to chromatin from the seed maturation stage rather than regulation by newly synthesized proteins. However, the authors provide no experimental evidence demonstrating that the CHX concentration used effectively inhibited translation. Validation is essential to support this critical interpretation of negative results.
While the authors report comparable overall quantities of newly synthesized RNA between CHX and mock treatments, the specific genes affected differ significantly. At 3h imbibition, CHX treatment shows increased new mRNA synthesis for more genes relative to mock, whereas at 6h, CHX results in decreased synthesis for more genes. Based on this, the authors conclude that early translation inhibition may enhance transcription of certain genes, while later-phase transcription requires regulation by newly synthesized protein factors. This interpretation appears insufficiently rigorous, as alternative explanations exist. Crucially, despite substantial differences in new mRNA profiles between CHX and mock, total RNA levels remain similar. This suggests potential compensatory regulation through balanced RNA synthesis and degradation dynamics that maintain transcriptome homeostasis.
3. The authors provide no experimental evidence confirming the effectiveness of ABA and ANT treatments. This critical validation could be achieved by assessing seed germination phenotypes (e.g., germination percentage or radicle emergence) under these treatment conditions, as both compounds are established modulators of germination physiology.
4. During the enrichment of biotin-labeled RNAs, the authors used glycogen as a blocking agent. Is this approach methodologically sound? Glycogen is not a conventional blocking agent for streptavidin-based pulldowns, where proteins like BSA or non-specific nucleic acids (e.g., salmon sperm DNA) are typically employed to reduce non-specific binding. Please justify this choice or provide validation data demonstrating that glycogen effectively minimizes background without compromising RNA recovery specificity.
5. In a previous publication (https://doi.org/10.1105/tpc.19.00214), 4sU was reported to be toxic to Arabidopsis. Given that the concentration of 4tU used in this study is quite high (2 mM), it is reasonable to question whether the stress-responsive

genes in Cluster 1 were induced by the elevated levels of 4tU over time, rather than being naturally upregulated during the germination process.
6. Regarding the conservation or robustness of transcription waves across species, a correlation analysis of orthologous gene expression between Arabidopsis and B. napus is suggested to provide stronger support for the claims.
7. The normalization method between input and “new” RNA library is not clearly presented. It remains unclear whether the trace amount of spike-in RNA is considered as background noise during biotin enrichment or if it serves another purpose.
8. I understand that utilizing the 3’ RNA-seq is a cost-effective way. However, I am curious what is ratio of T-to-C mutation in the 3’ sequencing tag?
Minors,
1. The authors consistently use “4-tU” to refer to 4-thiouridine throughout the manuscript. However, in standard biochemical nomenclature, “4-sU” is the accepted abbreviation for 4-thiouridine, while “4-tU” specifically denotes 4-thiouracil. To prevent confusion, I recommend replacing all instances of “4-thiouridine” with “4-thiouracil” when referring to the nucleobase analog used in the labeling experiments.
2. In Figure S1C, the Y-axis is labelled “fold change”. If this value represents expression normalized only to a reference gene (e.g., UBC21) within the same sample group, rather than a comparison between distinct experimental groups, it would be more accurate and less misleading to label it as “relative expression”. Using “fold change” typically implies a comparison between different conditions or time points.
3. In the figure legend of Fig. S1, it is described that error bars are present in panels C and D, but they are not actually shown in the figures.
4. Regarding the clustering of differentially expressed genes (DEGs) in Figure 1C, which was performed using WGCNA. What specific parameters were applied for this analysis? To my understanding, WGCNA is typically used for co-expression network construction that groups genes into distinct modules. Does this mean the four gene clusters (C1-C4) shown in the figure correspond to the four modules identified by WGCNA?
5. In Fig. S4, panel I appears to lack its label. Please ensure all panels are clearly marked.

Reviewer #2

(Remarks to the Author)
In this manuscript, Krzyszton and colleagues investigate the activity and features of Pol II transcription during the early stage of seed imbibition. For decades, the field has been influenced by a persistent misconception, stemming from early, methodologically limited studies, that transcription is dispensable for seed germination. A key strength of this work lies in the authors’ use of the seed nicking technique, which enhances the uptake of alpha-amanitin and thereby provides convincing phenotypic evidence that Arabidopsis germination is absolutely dependent on transcription. Together with a recent report published in Nature Communications (from Questa group), these findings compellingly highlight the importance of transcription in germination, a conclusion that will be highly appreciated by the community. In addition, by employing 4-tU RNA-seq, smFISH, and Pol II ChIP, the authors present evidence consistent with transcriptional activity occurring during early imbibition. With above said, however, I remain unconvinced by the data regarding the scale of active transcription during early imbibition, as well as the regulatory logic and biological meaning of such events. My major concern centers on the reliance on 4-tU labeling coupled with 3′RNA-seq to detect newly synthesized RNA. In my view, this approach likely suffers from substantial limitations in analytical rigor and interpretive clarity, raising the possibility that the extent and magnitude of newly synthesized RNA are considerably overestimated. In addition, when explaining polyadenylated RNA-seq data (as for the 4-tU RNA-seq in this study), contributions of not only the transcription but also RNA decay should be considered. Below, I outline my concerns in detail.

1.Regarding Fig. 1, α-amanitin is less frequently used in plant research. To enhance the confidence of the results, please provide an estimate on how soon the transcription is inhibited after the treatment. Additionally, please consider testing the phenotype/mRNA expression using another transcription inhibitor.
2.Page 6, lines 17-24: Please briefly describe the data quality, for example, how many reads were mapped to how many transcripts, especially for the detection of new mRNAs. In addition, although the author mentioned that they have considered the non-specific binding of RNA to the streptavidin beads, it remains unclear to me how they determined the threshold and why such a threshold was chosen. Without knowing these details, it is difficult to agree with the author's conclusion.
3.Page 6, line 25-34: The author described their 4-tU labeled RNA-seq datasets. Although I am convinced that there is likely some active transcription early on during imbibition, I am very concerned about the analysis pipeline and the number of actively transcribing genes at 3 and 6 hrs. As I mentioned earlier, the number of genes confidently detected in the 4-tU data is critical. The author used DESeq2; the assumption here is that the 4-tU and input data have roughly an equal number of transcripts detected with similar read coverage, which is very unlikely to be true in this case. The 8723 genes actively transcribing at 3 hours is a very large number that requires very careful proof. The fact that the 4-tU data correlate very well with the input data (Fig. 2F) further casts the doubt that a large fraction of the 4-tU data is a carryover of the unlabeled RNAs.
4.Related to the previous point, the authors report detecting transcription of 8,000–10,000 genes at the early stage of imbibition. A critical issue here is the extent or levels of such transcription. An extreme scenario could be that most of the detected transcription reflects only very low-level events (for example, a gene being transcribed in just one out of 1,000 cells). If this is the case, then the number “over 8,000 genes” is quite misleading. I recommend that the authors explicitly address this issue in both the Abstract and Results sections, and provide supporting evidence to clarify the actual magnitude and biological significance of these transcriptional events.
5.The author performed the 3’end RNAs-seq, which captures the mRNA and depletes any nascent RNA that is unpolyadenylated. I also noted that instead of a pulse label, they treated the seeds with an extended period of time. It was reported that extended 4-sU/4-tU treatment can lead to altered RNA decay dynamics; therefore, the author needs to exclude the possibility that the levels of “new RNA” observed is due to altered RNA decay dynamics.
6.Page 6, line 30-31: The author found that the new RNA pool was inversely correlated with the effects of alpha-amanitin on

mRNA levels (Fig. 3E and S2D). This data actually substantiates my concerns raised above. When transcription is inhibited, the extent of mRNA alteration reflects the RNA decay instead of RNA synthesis. Thus, these results indicate that the new RNA pool detected is not a reflection of de novo transcription, but rather likely (at least partially) results from RNA decay. The effect of RNA decay when interpreting the 4-tU RNA data should be considered.

7.Page 9, line 31: It is odd that CHX induces new RNA synthesis of 2237 genes at 3hrs. An obvious possibility that needs to be excluded is that the new RNA detection method is not robust enough, resulting in significant variations in the number of new RNAs detected from batch to batch. Also, what's the relationship between the 2237 genes and the genes in C1? In page 10, line 12-13, the author claims the CHX treatment enhances the first wave of gene upregulation that is supposed to be the C1 cluster (Fig. 1D), but the C1 cluster only has 316 genes. Please clarify these discrepancies.

8.Page 10, lines 1 to 6: It is difficult to read and understand the rationale behind this part. Please specify what is plotted in Fig. S3E. In addition, I'm not convinced that this data proves the claim that newly produced proteins in 3hrs are necessary for the RNA upregulation in 6hrs. A similar argument is being mentioned in the summary, which should be substantiated with more evidence.

9.Page 12, line 26-28: Here, the author mentioned that some upregulation of mRNA is not due to active transcription, while it seems the author assumed that C1 and C2 clusters (Fig. 1D) are mainly due to transcriptional change. This is one of the impressions I left throughout the manuscript; there are lots of arguments/discussion-like sentences, but the evidence supporting them is often complicated or indirect, which reduces the readership of this manuscript. I suggest the author re-polish the manuscript and strive for clarity in the results section.

10.In Fig. 6A, the authors performed Pol II ChIP-seq analysis on dry seeds and imbibed seeds. An important comparison missing here is between the ChIP-seq signal and the new RNA identified by the author through 4-sU RNA-seq. This provides an opportunity to validate if the new RNA identified is indeed due to Pol II transcription. Also, the results in Fig. s7g seem to contradict those in Fig. 2A-C, as in 6 hours, the new RNA group detected in 1 hour displayed the highest level of Pol II, while the new RNA group detected in 6 hours showed almost no Pol II. These observations further my concerns related to the robustness of the new RNA detected. I would expect the Pol II profile change to occur before or at least in parallel with the new RNA change.

Minor point:

1.Please indicate the duration of α-amanitin treatment in the main text (Page 3, lines 21-22).

2.Page 3, Line 35, what is the judgment that the selected "stable mRNAs" are actually stable, especially during the early seed imbibition stage?

3.Page 6 line 11, two peaks of mRNA upregulation, please clarify in the main text the relationship between this upregulation and transcription.

4.Please briefly explain the selection of 4tU over 4sU for labeling the newly synthesized RNA.

5.The authors performed multiple batches of 4tU–labeled RNA-seq at 3 hours under different conditions. How well do these replicates agree with each other?

Version 1:

Reviewer comments:

Reviewer #1

(Remarks to the Author)
In this revision, the authors have addressed most of my concerns raised last time. I appreciate the inputs provided here and think it would be a valuable resource for 4sU labelling methodology, as the authors have included more control experiments this time. It is a good idea to have both unlabeled and labeled spike for nonspecific binding control and normalization reference. And I am almost convinced that the new transcription is robust and essential for seed germination. Nevertheless, I find it difficult to read due to the complex nature of this new methodology establishment and the lack of clarity in some data presentations. I would like to see a clearer and more precise description of the data processing, especially normalization and new RNA calling, as well as the underlying logic.

The authors note that non-specific binding of biotinylated RNA to streptavidin beads constitutes a major technical challenge in 4sU labeling assays. To assess background noise, the use of exogenous spike-in RNA is indeed a recommended practice. However, the authors simultaneously employ the same spike-in RNA for normalization—a strategy that may introduce systematic bias. Specifically, if non-specific binding varies across samples, the residual unlabeled spike-in RNA remaining after streptavidin bead purification will likewise vary, rendering it an unreliable internal reference for normalization. An alternative approach—using only the labeled fraction of spike-in RNA as the normalization standard, however challenges associated with mRNA quantification in RNA isolated from dry versus imbibed seeds remains. The supplementary methods indicate that the proposed normalization against an internal standard; revisiting this approach may offer greater robustness. In the case of B. napus, I cannot understand the logic for normalization.

Figure S2g: Does the grey shading denote mRNAs that are not newly synthesized during imbibition? The criterion used to classify transcripts as "new" versus "pre-existing" is not explicitly defined. Notably, transcripts annotated as "not new" exhibit relatively high C-to-T conversion ratios (e.g., up to 2.5%), which challenges the biological interpretability of this classification. It remains unclear whether a quantitative threshold—such as a minimum C-to-T ratio—or a statistically rigorous approach (e.g., significance testing against background mutation rate or technical noise) was applied to support the distinction. In contrast, Figure 2f adopts a more straightforward definition: any transcript bearing at least one T-to-C mutation is designated as "newly synthesized." If the Fig2f and FigS2g depict the same two transcript categories (new and no), their

reported C-to-T conversion ratios appear inconsistent, raising questions about methodological coherence or data interpretation.

Line 599 What is the exact amount of ERCC RNA added to the biotinylated total RNA.

I recommend relocating the description of normalization procedures for both control and T-to-C–converted samples in 3′ RNA-seq data from the Supplementary Bioinformatics section to the main Methods section, to improve accessibility and methodological transparency for readers. Apparently, more details are needed.

Reviewer #2

(Remarks to the Author)
In this revised version, the authors have addressed the concerns I raised previously. In particular, they estimated nascent RNA levels by incorporating both ERCC controls and 4sU-labeled spike-ins. With these controls, the data are much more convincing.

I have only one remaining suggestion. The authors show that the conversion ratio reaches approximately 40% of reads after enrichment. Although this is an impressive level of enrichment, it also implies that around 60% of the sequenced reads remain unconverted and may represent background. Under these conditions, the authors should clarify how they determine that the remaining unconverted reads do not lead to an overestimation of newly synthesized RNA. Relatedly, why not restrict the analysis to converted reads only? This point should be clarified in the revised manuscript before publication.

Version 2:

Reviewer comments:

Reviewer #1

(Remarks to the Author)
In this revision, the authors have clarified the normalization methodology, which incorporated both external and internal reference standards, and demonstrated that both approaches yield consistent conclusions. This comparative analysis constitutes a valuable contribution to the field and enhances the methodological rigor of the study. Furthermore, the authors have refined the descriptions of the experimental procedures and bioinformatic analyses, improving clarity and reproducibility. Having addressed all prior concerns, I have no further substantive questions. However, I recommend that the Discussion section include a concise summary of the key methodological aspects and their implications for interpretation and future applications.

Open Access This Peer Review File is licensed under a Creative Commons Attribution 4.0 International License, which permits use, sharing, adaptation, distribution and reproduction in any medium or format, as long as you give appropriate credit to the original author(s) and the source, provide a link to the Creative Commons license, and indicate if changes were

made.
In cases where reviewers are anonymous, credit should be given to 'Anonymous Referee' and the source.
The images or other third party material in this Peer Review File are included in the article's Creative Commons license, unless indicated otherwise in a credit line to the material. If material is not included in the article's Creative Commons license and your intended use is not permitted by statutory regulation or exceeds the permitted use, you will need to obtain permission directly from the copyright holder.
To view a copy of this license, visit https://creativecommons.org/licenses/by/4.0/

The following secure token has been created to allow review of record GSE297302 while it remains in private status: kjgpcmcwjparrmb.

Reviewer #1 (Remarks to the Author):

This is a significant and well-executed study that fundamentally challenges the long-standing paradigm that stored mRNAs alone suffice for early seed germination, demonstrating instead that early and widespread Pol II transcription is essential. The work is rigorous, employs multiple complementary techniques, and provides compelling evidence across two species. The findings substantially advance our understanding of seed biology. However, certain descriptions in the manuscript lack clarity, and some interpretations of the data could benefit from more rigorous framing. These aspects warrant further refinement to enhance precision.

We appreciate the Reviewer's positive feedback on our work, and we are grateful for the valuable comments, which greatly improved the manuscript. We have now performed additional analyses to confirm the specificity of the new mRNA enrichment, assess the toxic effects of 4-SU, and analyse mRNA decay upon CHX treatment. We also expanded the description of the bioinformatic analysis to improve clarity of our work. In the initial submission, we limited the description of data analysis due to manuscript length constraints.

Major Concerns:

1. The most significant concern is the authors' lack of transparency in describing the data processing and analytical methods. Although the manuscript states that data and analysis code have been deposited in public databases or websites, the absence of accessible links prevents independent verification of the results' reliability. As noted by the authors: "A detailed description of the data analysis is provided on the GitHub website (to be provided upon publication)."

We apologise for not providing sufficient transparency and explanation in our data analysis at that stage of manuscript submission. To enhance transparency, we: 1) published our data analysis and figure preparation code on GitHub (https://github.com/mk1859/seed_transcription); 2) included essential information about the data analysis steps in the manuscript; and 3) greatly expanded the Supplemental Bioinformatic Methods to clarify why specific data analysis approaches were chosen.

This issue is further illustrated by the inconsistent application of differential expression thresholds in the DESeq2 analyses. The reported $|\log_2 FC|$ cutoffs vary arbitrarily—ranging from >0 to >1.5 to >2—across different sections of the manuscript, leading to considerable confusion. Most critically, the methodology for data normalization—a fundamental step in the analysis—is inadequately described, undermining confidence in the analytical rigor.

We apologise once again for the lack of clarity. The data normalisation methods vary between experiments due to differences in their setups and objectives. In the differential gene expression analysis, where no global changes in total mRNA levels were anticipated (except for α-amanitin or CHX treatments), we employed standard DESeq or CPM normalisation, which accounts for all mRNA levels. Otherwise, we used ERCC spike-ins or preselected endogenous stable transcripts for normalisation. That approach was not adopted uniformly, as it can introduce higher technical noise. The ERCC or non-transcribed gene normalisation was used to account for nonspecific enrichment, and was therefore applied to new mRNA identification and new transcripts differential expression analysis. The rationale and control analysis for choosing specific gene sets for normalisation are now explained in the manuscript and further detailed in the Supplemental Bioinformatic Methods.

Importantly, in the revised manuscript, we verified the specificity of the new mRNA enrichment and identification using labelled spike-ins and the conversion of enriched mRNAs (Fig. 2 and S2).
Due to varying experimental setups, we used different |log_2FC| cut-offs consistently for different types of experiments. For differential gene expression and analysis of changes in mRNA levels, we use |log_2FC| > 1, but for new mRNA identification, we always use log_2FC > 2 to be more stringent in calling. A cut-off of 1.5- and 3-fold change was used only to identify transcription sites in the smFISH microscopic data analysis. Those differences are now more clearly described in the manuscript, explained in the Supplemental Bioinformatic Methods, and detailed in the GitHub code.

2. In the third section of the Results, the authors treated seeds with the translation inhibitor CHX. They report minimal differences in total RNA levels between CHX- and mock-treated samples at 3h and 6h, concluding that early transcription relies primarily on transcriptional machinery pre-bound to chromatin from the seed maturation stage rather than regulation by newly synthesized proteins. However, the authors provide no experimental evidence demonstrating that the CHX concentration used effectively inhibited translation. Validation is essential to support this critical interpretation of negative results.

We thank the Reviewer for this valid remark. To demonstrate that in our setup, cycloheximide (CHX) inhibits translation during early water uptake in seeds, we used a reporter line (3xmCherry) driven by a DEX-inducible promoter (Doumane *et al.*, 2021). 6h imbibition in the presence of DEX allows detection of the 3xmCherry protein by western blot, whereas seeds not induced with DEX and seeds imbibed in the presence of both DEX and CHX do not show 3xmCherry protein expression. This result validates CHX-mediated translation inhibition during early seed germination and is now included as Supplementary Fig. S3A.

While the authors report comparable overall quantities of newly synthesized RNA between CHX and mock treatments, the specific genes affected differ significantly. At 3h imbibition, CHX treatment shows increased new mRNA synthesis for more genes relative to mock, whereas at 6h, CHX results in decreased synthesis for more genes. Based on this, the authors conclude that early translation inhibition may enhance transcription of certain genes, while later-phase transcription requires regulation by newly synthesized protein factors. This interpretation appears insufficiently rigorous, as alternative explanations exist. Crucially, despite substantial differences in new mRNA profiles between CHX and mock, total RNA levels remain similar. This suggests potential compensatory regulation through balanced RNA synthesis and degradation dynamics that maintain transcriptome homeostasis.

We are thankful for these valuable comments. First, we aimed to confirm our observations, which is why we conducted several additional experiments on Pol II transcription and mRNA level changes during translation inhibition. We repeated the new and total mRNA 3'RNA-seq at 6h of CHX treatment, obtaining highly consistent results with the initial experiment (Fig. S3F-I). In fact, the primary purpose of that experiment was to serve as a control for the puromycin treatment, another translation inhibitor with a different mechanism of action. That treatment provided results consistent with both CHX treatments (Fig. S3F-I). The differences in the effects of the two inhibitors may be explained by their distinct modes of action and distinct effects on mRNA stability (Kramer, 2017). Next, we validated changes in new mRNA levels at 6h of CHX treatment using RT-qPCR. RT-qPCR is generally noisier than RNA-seq, as it depends on a single reference gene rather than robust normalisation across a set of transcripts. Nevertheless, we observe that most tested genes show changes in line with 3' RNA-seq (Fig. S3J). These experiments confirm the reproducibility of our findings.
Next, we sought to exclude the possibility that a global mRNA abundance shift affected normalisation and differential gene expression analysis following CHX treatment. We recalculated

the initial 3'RNA-seq for CHX treatment and the new one with CHX and puromycin treatments, using added ERCC spike-ins for normalisation in total mRNA analysis. This produced results very similar to those in the original submission in terms of the number of affected genes, suggesting no strong genome-wide changes in total mRNA levels (Fig. S3C and F).

As suggested by the Reviewer, to compensate for increased transcription, CHX treatment should decrease mRNA stability if total mRNA levels are unchanged. Precise measurement of mRNA half-lives is not feasible during seed imbibition because the mRNA pool and transcriptional profiles are significantly reshaped. That is why we directly compared seeds imbibed in the presence of α-amanitin with those incubated with both α-amanitin and CHX (Fig. S3K). To our surprise, differential comparison suggested upregulation of a large group of total mRNAs in response to CHX treatment (Fig. S3L-M). Notably, such stabilisation was shown earlier (Kramer, 2017). A closer examination of both ERCC-normalised CHX treatments without α-amanitin, indeed showed that these mRNAs tend to be upregulated, but mostly do not exceed a log2FC threshold of 1 (Fig. S9I).

These observations suggest that mRNA stabilisation could lead to increased detection of new mRNA after CHX treatment (Fig. S3N). The most straightforward explanation is nonspecific binding of total mRNA misinterpreted as enhanced transcription. We demonstrated, using labelled spike-ins and conversions, that enrichment of newly transcribed mRNAs is largely specific (Fig. 2 and S2). Therefore, the effect caused by nonspecific binding of total mRNAs should be more noticeable for mRNAs with lower enrichment levels, due to a greater contribution from total nonspecific mRNAs. Notably, this is not the case (Fig. S9J). Another possibility is that the binding is specific as expected, but CHX treatment also stabilises new 4-SU-labelled mRNAs. It is probable that newly synthesised mRNAs in seeds are more stable than older ones, which could accumulate damage during dry seed storage. Testing this hypothesis is challenging because it would require first labelling with 4-SU, then washing out the nucleotide or applying an inhibitor, which would be inefficient in already imbibed seeds. These scenarios suggest that the upregulation of new mRNAs upon CHX treatment conceivably does not, to some extent, depend on transcription. However, it is also possible that the inhibition of new mRNA synthesis by α-amanitin is delayed or incomplete (Fig. S9C and 1E), and that the increased detection actually reflects genuine new mRNAs with heightened transcription due to CHX treatment. We cannot distinguish between these possibilities, and it is likely that all play a role in the increased detection of new mRNAs following CHX treatment. That is why we no longer claim that CHX causes increased transcription, but only that it results in increased detection of some new mRNAs, which is likely at least partly due to their stabilisation.

It is important to emphasise that the second part of our claim, which states that the block of translation inhibits the activation of transcription of another group of genes, still holds. In fact, reports of a similar phenomenon in the inflammatory response have been published (Yamazaki and Takeshige, 2008). During the inflammatory response, the first responsive mRNAs are stabilised by translation inhibition, and the transcriptional activation of the second wave is blocked. That exactly corresponds to our interpretation of the seed's response to water uptake in the presence of CHX.

3. The authors provide no experimental evidence confirming the effectiveness of ABA and ANT treatments. This critical validation could be achieved by assessing seed germination phenotypes (e.g., germination percentage or radicle emergence) under these treatment conditions, as both compounds are established modulators of germination physiology.

We have now performed germination assays on both intact and nicked seeds, with similar results. ABA blocks seedling establishment, just after the testa-rupture stage. Antabactin (ANT) does not affect the germination of after-ripened seeds. Treatment with both ABA and ANT reverses ABA's inhibitory effect, validating ANT's activity. These results are now included as Supplementary Fig. S4K.

4. During the enrichment of biotin-labelled RNAs, the authors used glycogen as a blocking agent. Is this approach methodologically sound? Glycogen is not a conventional blocking agent for streptavidin-based pulldowns, where proteins like BSA or non-specific nucleic acids (e.g., salmon sperm DNA) are typically employed to reduce non-specific binding. Please justify this choice or provide validation data demonstrating that glycogen effectively minimizes background without compromising RNA recovery specificity.

To avoid extensive optimisation steps, we closely followed existing protocols that appeared suitable for use with RNA from seed samples. Seed RNA is well known to copurify with storage polysaccharides and sugar-containing polymers present in the seed coat mucilage layer (Meng and Feldman, 2010). For these reasons, we blocked the beads with glycogen as described, for example, by (Barrass *et al.*, 2015) and (Swiatkowska *et al.*, 2012).

5. In a previous publication (https://doi.org/10.1105/tpc.19.00214), 4sU was reported to be toxic to Arabidopsis. Given that the concentration of 4tU used in this study is quite high (2 mM), it is reasonable to question whether the stress-responsive genes in Cluster 1 were induced by the elevated levels of 4tU over time, rather than being naturally upregulated during the germination process.

C1 cluster was identified as early upregulated in the absence of 4-SU in the analysis of total mRNAs in the mock control for the α-amanitin time-course experiment (Fig. 1). It also shows the highest Pol II enrichment during imbibition, as verified by ChIP-seq (Fig. S7H, bottom). So its identification was not dependent on the presence of 4-SU in the imbibition solution. We now also performed a control experiment in which we avoided prolonged incubation with 4-SU. The main limitation of brief labelling is that the uptake of 4-SU into already imbibed seeds is low, even when seeds are nicked before imbibition. To enhance uptake, a large number of intact seeds were first imbibed for 2.5 h or 5.5 h, then squeezed between two microscopic glass slides, yielding a mixture of seed coats and both intact and fragmented embryos. Such a mixture was incubated in ½ MS medium supplemented with 4-SU for 0.5h. This approach was not chosen first because it required destroying the seeds before labelling. Nevertheless, analysis of such briefly labelled samples yielded results highly concordant with continuous labelling (Fig. S2J-K). Finally, we performed 5-EU labelling (assumed to be less toxic than 4-SU; (Szabo *et al.*, 2020)) followed by enrichment, reverse transcription on beads and qPCR. Tested two genes from the C1 cluster showed new mRNA production, as their enrichment recovered a high percentage of the input (Fig. S2L).

6. Regarding the conservation or robustness of transcription waves across species, a correlation analysis of orthologous gene expression between Arabidopsis and B. napus is suggested to provide stronger support for the claims.

Thank you for this remark. We now provide plots showing a high correlation of enrichment during new mRNA identification in *A. thaliana* and *B. napus*. Since *B. napus* imbibition appears to be delayed compared to Arabidopsis, the most similar results are observed at different time points. These plots are now presented in Fig. 5E and S6H.

7. The normalization method between input and "new" RNA library is not clearly presented. It remains unclear whether the trace amount of spike-in RNA is considered as background noise during biotin enrichment or if it serves another purpose.

We apologise for this lack of clarity. Spike-ins serve an important role in our method for identifying new mRNAs. Detection of mRNA among transcripts bound by the beads in the enrichment step cannot be considered as proof that it is newly produced. As the reviewer noted, there is always some level of nonspecific binding of unlabelled mRNAs, which is readily detectable with highly sensitive

sequencing approaches. It was reported that up to 30% of bead-bound RNAs can be unspecific carry-over (Duffy, Schofield and Simon, 2019). This is very well illustrated by the binding of unlabelled ERCC spike-ins in our experiments. This, however, can be an advantage because their levels can be compared with those of mRNAs, allowing identification of mRNAs showing nonspecific or irreproducible enrichment. Computationally, we achieve this by treating ERCC spike-ins as a group of normalisation transcripts between the total and enriched samples. After normalisation, we select transcripts that show strong and reproducible upregulation relative to the input (DESeq2). This is now more clearly stated in the manuscript, the Supplemental Bioinformatic Methods, and the comments to the GitHub code.

8. I understand that utilizing the 3′ RNA-seq is a cost-effective way. However, I am curious what is ratio of T-to-C mutation in the 3′ sequencing tag?

For the first version of the manuscript, we converted total mRNA samples in which newly produced labelled mRNAs constitute only a small fraction. As a result, the conversion ratio (T -> C) is, on average, only 0.25%, but higher than in unconverted samples (0.09% - PCR and sequencing errors). This is also illustrated by Sanger sequencing of RT-PCR products for selected mRNAs in Fig. S2B. For the new version of the manuscript, we repeated this experiment, including the conversion of samples that had been earlier enriched using biotin attachment. This was to confirm that mRNAs bound to the beads are mostly labelled and are not a nonspecific carryover. For the new set, we observed a 0.15% conversion rate across total mRNAs, compared to 0.04% in the negative control, while bead-enriched mRNAs showed a 3.3% conversion rate. These numbers may be considered low, but when we count reads containing a converted nucleotide rather than directly counting conversions, we observe 42% of converted reads for mRNAs bound by the beads, compared to 2.6% in the total mRNA sample and 1.17% in the negative control. A relatively high level of observed mutations in non-converted samples is consistent with the effects of 4-SU on reverse transcription (Herzog *et al.*, 2017). At the same time, the positive control spike-in (UTP was replaced with 4-sUTP during synthesis) shows a high fraction of converted reads, both in total and enriched samples. However, it is important to note that, despite all Us in the spike-in transcripts being 4-SU, the conversion ratio is relatively low, suggesting that this approach may underestimate the fraction of new transcripts for endogenous mRNAs. This suggests that relying on a number of reads with a conversion rather than the absolute level of C-to-T conversion events is more reliable for estimating the recovery of true new mRNA levels. All these results are now presented in Fig. 2F and S2G. A relatively low level of conversion of endogenous transcripts may be explained by a few factors: 1) 4-SU needs to be first phosphorylated to 4-sUTP, which, under conditions of restarting seed metabolism, may not be a fast process. 2) Labelled nucleotides need to compete with the endogenous pool of stored nucleotides. 3) Even enriched samples may not show a high level of read conversion, as we sequence only the 3′ end of mRNA, and conversion (and biotin attachment) could take place in the unsequenced part of the molecule.

Minors,
1. The authors consistently use "4-tU" to refer to 4-thiouridine throughout the manuscript. However, in standard biochemical nomenclature, "4-sU" is the accepted abbreviation for 4-thiouridine, while "4-tU" specifically denotes 4-thiouracil. To prevent confusion, I recommend replacing all instances of "4-thiouridine" with "4-thiouracil" when referring to the nucleobase analog used in the labeling experiments.

We apologise for the confusion; this was our mistake. Throughout the experiments, we have consistently used 4-SU instead of 4-tU. We have now corrected this and applied the proper nomenclature throughout the manuscript.

2. In Figure S1C, the Y-axis is labelled “fold change”. If this value represents expression normalized only to a reference gene (e.g., UBC21) within the same sample group, rather than a comparison between distinct experimental groups, it would be more accurate and less misleading to label it as “relative expression”. Using “fold change” typically implies a comparison between different conditions or time points.

In this case, the plot shows expression data, first normalised to UBC21 and then compared with the control condition (imbibition of intact seeds). Accordingly, we change the description of this and the corresponding axes to “*UBC21* normalised fold change”.

3. In the figure legend of Fig. S1, it is described that error bars are present in panels C and D, but they are not actually shown in the figures.

Thank you for spotting this mistake. That was a copy-paste error – we did not include error bars as we presented data points for all biological replicates.

4. Regarding the clustering of differentially expressed genes (DEGs) in Figure 1C, which was performed using WGCNA. What specific parameters were applied for this analysis? To my understanding, WGCNA is typically used for co-expression network construction that groups genes into distinct modules. Does this mean the four gene clusters (C1-C4) shown in the figure correspond to the four modules identified by WGCNA?

That is correct. Clusters C1-C4 correspond to the four modules identified by WGCNA, and this is now clarified in the manuscript. The WGCNA parameters are detailed in the GitHub code description.

5. In Fig. S4, panel I appears to lack its label. Please ensure all panels are clearly marked.

Thank you for noticing the error. It has now been corrected.

In summary, we would like to thank the Reviewer for pointing out the 4-SU – 4-tU misunderstanding, proposing important controls, and asking key questions, which prompted us to improve our work. We strongly believe that by including the additional controls, including the spike-in, and tuning down our conclusions, for example, about the contribution of RNA stability/new RNA production, the manuscript became stronger and ready for publication.

Reviewer #2 (Remarks to the Author):

In this manuscript, Krzyszton and colleagues investigate the activity and features of Pol II transcription during the early stage of seed imbibition. For decades, the field has been influenced by a persistent misconception, stemming from early, methodologically limited studies, that transcription is dispensable for seed germination. A key strength of this work lies in the authors’ use of the seed nicking technique, which enhances the uptake of alpha-amanitin and thereby provides convincing phenotypic evidence that Arabidopsis germination is absolutely dependent on transcription. Together with a recent report published in Nature Communications (from Questa group), these findings compellingly highlight the importance of transcription in germination, a conclusion that will be highly appreciated by the community. In addition, by employing 4-tU RNA-seq, smFISH, and Pol II ChIP, the authors present evidence consistent with transcriptional activity occurring during early imbibition. With above said, however, I remain unconvinced by the data regarding the scale of active transcription during early imbibition, as well as the regulatory logic and biological meaning of such events. My major concern centers on the reliance on 4-tU labeling coupled with 3’RNA-seq to detect newly synthesized RNA. In my view, this approach likely suffers from substantial limitations in analytical rigor and interpretive clarity, raising the possibility that the extent and magnitude of newly synthesized RNA are considerably

overestimated. In addition, when explaining polyadenylated RNA-seq data (as for the 4-tU RNA-seq in this study), contributions of not only the transcription but also RNA decay should be considered. Below, I outline my concerns in detail.

We are grateful for both positive and critical remarks on our manuscript, which led to the preparation of an improved version. In particular, we included additional experiments to demonstrate the specificity of labelled new mRNA enrichment, quantify its magnitude, and verify the toxic effect of 4-SU treatment. We believe the main issue raised by the Reviewer concerns the specificity of new mRNA identification, both in experimental design and in bioinformatic processing of the results. We tried to address all these concerns in the following section of the response, but at this point, we wanted to highlight the additional experiments and analyses that, in our opinion, support our results:

1. We repeated the identification of the new mRNAs during the time course of seed imbibition, which yielded highly consistent results. Importantly, in this analysis, we used labelled spike-ins and observed their increasing competition for binding to endogenous mRNAs over time. This should not have been the case if binding to the beads is mostly nonspecific.

2. We performed the conversion of total and enriched labelled RNA. This independent method showed that enriched mRNAs are newly transcribed. Importantly, the added spike-in (all U replaced with 4-SU) showed that the conversion is not complete, which suggests that even non-converted enriched endogenous mRNAs most likely contain 4-SU.

3. We show that when genes are binned based on mRNA conversion levels, the Pol II levels analysed by ChIP correspond to new mRNA production.

4. We performed 5-EU labelling, followed by enrichment and RT-qPCR analysis. This showed that the majority of transcripts identified as newly produced by 4-SU 3'RNA-seq are also identified as new using the orthogonal 5-EU approach.

5. We performed short labelling with 4-SU, which showed a consistent pattern of new mRNA detection with continuous labelling, suggesting that possible 4-SU-induced mRNA metabolism defects have only a minor contribution to the identification of new mRNA in our setup.

6. We identified a group of endogenous mRNAs with a range of levels and consistent ERCC-like enrichments. This shows that nonspecific carryover is not a major factor shaping a pool of enriched mRNAs.

7. We show the high consistency of new mRNA identification, using both external spike-ins and the mentioned above pool of endogenous transcripts, for several experiments performed.

1. Regarding Fig. 1, α-amanitin is less frequently used in plant research. To enhance the confidence of the results, please provide an estimate on how soon the transcription is inhibited after the treatment. Additionally, please consider testing the phenotype/mRNA expression using another transcription inhibitor.

We are grateful to the Reviewer for these questions. First differences between mock- and α-amanitin-treated samples can be observed using 3'RNA-seq as early as the 1h time point (Fig. 1B-C). We confirmed this using RT-qPCR and observed a modest decrease in total mRNA levels at 1h for genes whose expression was strongly induced during imbibition (Fig. S1D). Given that the restart of metabolic activity takes some time during imbibition, we consider this a very early inhibition. We also tested cordycepin as an inhibitor and compared it with α-amanitin at the 6h time point of

imbibition using RT-qPCR, with both inhibitors showing a similar decrease in mRNA levels (Fig. S1F). As shown before (Bai *et al.*, 2018), cordycepin completely blocked germination (Fig. S1E), and flavopirodol (Shu *et al.*, 2022) blocked germination after testa rupture (Fig. S1E). We were aware of these observations, but we decided to use α-amanitin for several reasons. Among these, the most important finding was that cordycepin may not only inhibit Pol II transcription but also affect the activities of other polymerases, including those involved in poly(A) tail formation.

2. Page 6, lines 17-24: Please briefly describe the data quality, for example, how many reads were mapped to how many transcripts, especially for the detection of new mRNAs. In addition, although the author mentioned that they have considered the non-specific binding of RNA to the streptavidin beads, it remains unclear to me how they determined the threshold and why such a threshold was chosen. Without knowing these details, it is difficult to agree with the author's conclusion.

We apologise for not providing this information and for the lack of clarity. The table showing sequencing depths for each library and the number of deduplicated counts assigned to genes is now provided in Supplementary Table S2. In our work, we mostly use 3' RNA-seq with a limited number of reads to accommodate a large number of testes conditions, followed by UMI-based removal of PCR duplicates. This strategy yields low counts but is highly correlated with true mRNA levels. We agree that when read counts are highly imbalanced, normalisation can lead to artefacts. In fact, in some experiments, the number of identified deduplicated reads is low; nevertheless, these experiments are highly consistent with those in which we obtained a higher number of reads (Fig. S9D-F). We also identified a set of endogenous transcripts with spike-in-like behaviour that covers a range of levels. Their low enrichment level is consistent across experiments, suggesting that normalisation-related artefacts are unlikely.
To determine the levels of unspecific (unlabelled) transcript binding to the beads, we initially used commercially available ERCC spike-ins (Thermo Fisher Scientific). Before the enrichment step, ERCC spike-ins were added to the RNA sample. A small fraction of the sample was used to prepare the input mRNA library, and the remaining fraction was biotinylated, enriched with streptavidin beads, and used to prepare the new mRNA library. As expected, the ERCC spike-ins are always detected in the enriched fraction, albeit at lower levels than in the input. Importantly, their presence allows comparison of mRNA levels between input and new mRNA samples to calculate their enrichment. The easiest way to achieve this is by using ERCC spike-in amounts as a normalisation factor in the DESeq2 pipeline. As a result, we obtain an adjusted p-value reflecting the consistency of the biological replicates and a log2 fold change indicating enrichment relative to the input. To be stringent, we removed low-count genes before normalisation and used a high threshold (log2FC enrichment > 2) to identify new mRNAs. We also used the same strategy (log2FC < 1) to identify endogenous mRNAs with ERCC spike-in-like enrichment, which we classified as non-transcribed. That set of transcripts can constitute an alternative means of normalising and comparing input and enriched samples based on endogenous mRNAs (see Supplemental Bioinformatic Methods). The choice of these thresholds for mRNA classification (new and non-transcribed) was arbitrary, but the results are highly reproducible across experiments (Fig. S2E and S9D-F).
Comparing our multiple ERCC spike-in experiments, we noticed variations caused by unavoidable small technical differences in ERCC content, as also reported by (Lin *et al.*, 2020) and (Zhang *et al.*, 2026). In principle, using a non-transcribed set of endogenous transcripts would possibly yield even more consistent results, but here we preferred to use external ERCC spike-ins for clarity in the experimental design. Our rationale for using the spike-ins and thresholds setup, and the advantage of using a non-transcribed set of endogenous transcripts, is now more clearly described in the manuscript, the Supplemental Bioinformatic Methods, and the comments in the GitHub code (https://github.com/mk1859/seed_transcription).

3. Page 6, line 25-34: The author described their 4-tU labeled RNA-seq datasets. Although I am convinced that there is likely some active transcription early on during imbibition, I am very concerned about the analysis pipeline and the number of actively transcribing genes at 3 and 6 hrs. As I mentioned earlier, the number of genes confidently detected in the 4-tU data is critical. The author used DESeq2; the assumption here is that the 4-tU and input data have roughly an equal number of transcripts detected with similar read coverage, which is very unlikely to be true in this case. The 8723 genes actively transcribing at 3 hours is a very large number that requires very careful proof. The fact that the 4-tU data correlate very well with the input data (Fig. 2F) further casts the doubt that a large fraction of the 4-tU data is a carryover of the unlabeled RNAs.

We are grateful for this comment, as it prompted us to perform additional experiments that improved our manuscript. The main challenge in identifying newly produced mRNAs during seed imbibition is that seeds already store many mRNAs from the maturation stage. This means that the quantitative contribution of newly transcribed transcripts to the existing transcriptome may be minor, especially during early imbibition, when Pol II activity resumes. Another caveat is that 4-SU from the imbibition solution must reach the seed cells, be converted to 4-SUTP, and be incorporated into new transcripts while competing with the pool of stored endogenous UTP. As a result, even for actively transcribed genes, 4-SU incorporation may be delayed and low. Based on 4-SU properties, we used two alternative procedures to identify new mRNAs.

The first strategy is based on biotin attachment and streptavidin bead enrichment, which is sensitive but does not allow quantification of the absolute amount of newly synthesised mRNA. As described in the answer to the previous Reviewer's point, thanks to the addition of unlabelled ERCC spike-ins and stringent filtering of sequencing results, we can account for unspecific transcript binding to the beads. This strategy, which always compares enriched samples to the input, also accounts for potential changes in total mRNA levels. To improve our manuscript, we wanted to estimate the sensitivity of detecting labelled transcripts during streptavidin-mediated enrichment. For this, we prepared eight positive spike-ins transcribed in the presence of a mixture of 4-SUTP:UTP (1:3), and then mixed them with the same spike-ins transcribed only with UTP at different ratios (from 1% to 20% labelled transcript). These mixed spike-ins were added to labelled seed RNA samples together with negative ERCC spike-ins. Two spike-ins (A and B) with the highest labelled fraction show consistently high enrichment at 1h, 3h, and 6h timepoints (Fig. 2B-D). For spike-ins with lower levels of labelled transcript (for example, C-H in Fig. 2B-D), enrichment levels decrease over time during imbibition. This may suggest that the amount of streptavidin beads is a limiting factor at the 6h timepoint, resulting in the exclusion of some lowly expressed genes from being classified as transcribed. To independently confirm the enrichment approach, we also performed 5-EU labelling, irreversible biotin attachment via click chemistry, streptavidin enrichment, reverse transcription, and qPCR analysis along with the input samples. This independent method showed high overlap with mRNAs enriched using 4-SU labelling (Fig. S2L).

A different approach based on 4-SU labelling is the conversion of labelled transcripts that leads to T into C change during cDNA synthesis. In the initial version of the manuscript, this experiment was performed on total mRNA, but not with positive and negative spike-ins. Now, we added negative-control ERCC spike-ins and a positive spike-in (100% labelling), and also included samples that were first streptavidin-bead-enriched before conversion to independently estimate the amount of unlabelled mRNA bound by the beads. The conversion level in total labelled mRNAs is low but consistent with the previous experiment (Fig. 2F). The low conversion across total mRNAs can be explained by the factors mentioned at the beginning of this response, i.e., a large amount of stored mRNA and delayed labelling. Importantly, the conversion rate for enriched samples showed at least one conversion in 42% of reads on average (with ERCC close to zero and positive spike-in close to

100%) (Fig. 2F). Although this appears to support the Reviewer's suspicion that most mRNA bound to the beads is nonspecific, a detailed examination of the conversion ratio for individual U residues shows it is relatively low, even with a 100%-labelled positive spike-in. This indicates that the conversion may not be efficient and could underestimate the actual level of labelling (Fig. S2G). This issue may be especially significant for newly synthesised endogenous transcripts, in which only a small fraction of U was replaced by 4-SU due to competition with the endogenous UTP pool. It is also important to note that we used 3' RNA-seq, and the labelled nucleotide could be present in unsequenced regions of the transcript. To sum up, we observed that at least 42% of the beads bound endogenous mRNA is newly produced, a number that is most likely largely underestimated due to inefficient conversion. It is important to note that, although inefficient, conversion of the total mRNA sample may be the most direct way to estimate the magnitude of new mRNA production relative to the total mRNA amount. Nevertheless, the scarcity of conversions prevents statistical analysis that would enable independent, enrichment-free calling of the new mRNAs.
Finally, we want to point out that we disagree with the Reviewer that 8,723 transcribed genes is a large number. Using 5-EU labelling, Szabo et al. (Szabo *et al.*, 2020) identified above 20,000 genes producing new transcripts in Arabidopsis seedlings. We cannot see why metabolically active seed cells (at 3h or 6h of imbibition) should have much lower transcriptional activity. Also, the resemblance between the input and the new mRNA fraction may be expected. The seedling establishment process is still ahead of the seed, so it still expresses seed-characteristic transcripts that were, to varying degrees, produced during seed maturation and stored. This comparison of the number of transcribed genes has been added to the discussion section. We want to stress that the resemblance of maturation and imbibition transcriptional activity is not complete. As we described in our manuscript, there is a group of seed maturation-highly-expressed genes that we cannot detect among the new mRNAs (Fig. S2N-P).

4. Related to the previous point, the authors report detecting transcription of 8,000–10,000 genes at the early stage of imbibition. A critical issue here is the extent or levels of such transcription. An extreme scenario could be that most of the detected transcription reflects only very low-level events (for example, a gene being transcribed in just one out of 1,000 cells). If this is the case, then the number "over 8,000 genes" is quite misleading. I recommend that the authors explicitly address this issue in both the Abstract and Results sections, and provide supporting evidence to clarify the actual magnitude and biological significance of these transcriptional events.

As previously mentioned, quantifying newly synthesised mRNA in absolute terms (for example, using 4-SU conversion) is challenging because of the large amounts of stored mRNA. During the 6-hour imbibition period, seeds likely cannot replace a significant portion of the stored mRNAs with new transcripts. Transcription during early imbibition can produce relatively low levels of new mRNA compared to the amount of stored mRNA, but this does not necessarily indicate low transcription. Our analysis pipeline excludes mRNAs with low detection levels and focuses on qualitative rather than quantitative description of new mRNA production during imbibition, by comparing new and total (mostly stored) mRNA fractions. Importantly, as explained in detail above, new results obtained using positive spike-ins and conversion analysis suggest that we rather underestimate than overestimate the extent and magnitude of the Pol II transcription during early seed imbibition. We do not believe the number of genes identified as transcribed during seed imbibition is overestimated, especially in light of results from Arabidopsis seedlings (Szabo *et al.*, 2020).
While we demonstrated that application of Pol II inhibitors blocks germination, explicitly demonstrating the biological importance of new transcription during the first 6 hours of imbibition is largely impractical. Using a transcription inhibitor on scarified seeds indicated that transcription is necessary, but it cannot pinpoint the precise timing, since the ultimate result is simply a failure to

germinate. Initial incubation with the inhibitor, followed by washing it out, might only lead to a temporary delay in restart transcription, rather than a treatment that would discriminate between the roles of early and late transcription.
In summary, we disagree with the Reviewer's view that the absence of absolute quantification of new mRNAs is a significant limitation. However, we have added clarification distinguishing between qualitative and quantitative detection of new mRNAs in the Results and Discussion sections of the revised manuscript.

5. The author performed the 3'end RNAs-seq, which captures the mRNA and depletes any nascent RNA that is unpolyadenylated. I also noted that instead of a pulse label, they treated the seeds with an extended period of time. It was reported that extended 4-sU/4-tU treatment can lead to altered RNA decay dynamics; therefore, the author needs to exclude the possibility that the levels of "new RNA" observed is due to altered RNA decay dynamics.

We are grateful for this comment. Our primary goal was not to study nascent transcriptomes, which would require specialised methods, such as enriching for chromatin-bound Pol II complexes and sequencing the associated RNAs (Leng *et al.*, 2020). In our work, we aimed for a qualitative description of the extent of new mature mRNA production. For this, stabilising briefly transcribed mRNAs enhances the likelihood of their detection, which aligns with the experimental goals. Nevertheless, for the new version of the manuscript, we performed a pulse labelling experiment. As described in the response to point 5 in the remarks of Reviewer 1: "We now also performed a control experiment in which we avoided prolonged incubation with 4-SU. The main limitation of brief labelling is that the uptake of 4-SU into already imbibed seeds is low, even when seeds are nicked before imbibition. To enhance uptake, a large number of intact seeds were first imbibed for 2.5 h or 5.5 h, then squeezed between two microscopic glass slides, yielding a mixture of seed coats and both intact and fragmented embryos. Such a mixture was incubated in ½ MS medium supplemented with 4-SU for 0.5h. This approach was not chosen first because it required destroying the seeds before labelling. Nevertheless, analysis of such briefly labelled samples yielded results highly concordant with continuous labelling (Fig. S2J-K)." We also performed 5-EU labelling (assumed to be less toxic than 4-SU (Szabo *et al.*, 2020)), followed by enrichment, reverse transcription on beads and qPCR. This approach also yielded results in high agreement with 4-SU enrichment and sequencing (Fig. S2L).

6. Page 6, line 30-31: The author found that the new RNA pool was inversely correlated with the effects of alpha-amanitin on mRNA levels (Fig. 3E and S2D). This data actually substantiates my concerns raised above. When transcription is inhibited, the extent of mRNA alteration reflects the RNA decay instead of RNA synthesis. Thus, these results indicate that the new RNA pool detected is not a reflection of de novo transcription, but rather likely (at least partially) results from RNA decay. The effect of RNA decay when interpreting the 4-tU RNA data should be considered.

The Reviewer suggests that, as α-amanitin causes a specific trend due to transcription blockage, the opposite trend observed with 4-SU treatment may indicate a block in mRNA decay. This may, in turn, somehow lead to enhanced nonspecific mRNA enrichment misinterpreted as new mRNAs. We disagree with this interpretation of the presented results. Most importantly, to identify genes producing new mRNA, we use ratios of new mRNA levels to total mRNA levels normalised to ERCC spike-in levels, so any changes in total mRNA abundance are accounted for. Second, our conversion experiment on the beads enriched mRNAs at 6h shows that they contain at least 42% labelled transcripts, which is most likely an underestimation for the reasons described above. Also, new mRNAs identified after a half-hour labelling experiment at 3h and 6h time points (pulse) are consistent with continuous labelling (Fig. S2J-K), suggesting that the pattern of new mRNA production does not arise from a toxic effect of 4-SU. This is further supported by RT-qPCR analysis

after 5-EU labelling (assumed to be less toxic than 4-SU; Szabo et al., 2020), consistent with 4-SU labelling (Fig. S2L).

The strongest downregulation upon α-amanitin treatment is observed for transcripts normally upregulated during imbibition. This is consistent with those genes showing the highest transcription and consequently the highest enrichment and conversion after 4-SU labelling. The open question is how the presence of incorporated 4-SU affects mRNA metabolism or its detection. Recent studies show that highly labelled, short-lived transcripts are underrepresented in libraries, largely due to technical limitations (Berg *et al.*, 2024). This again may suggest that our procedure underestimates the extent of new transcription in seeds. But even if the presence of 4-SU stabilises mRNAs, we consider this an enhancement of the method's sensitivity rather than a major drawback. Importantly, even if such stabilisation exists, it will not lead to overestimation of the number of genes producing new mRNAs. This clarification is now added to the discussion.

7. Page 9, line 31: It is odd that CHX induces new RNA synthesis of 2237 genes at 3hrs. An obvious possibility that needs to be excluded is that the new RNA detection method is not robust enough, resulting in significant variations in the number of new RNAs detected from batch to batch. Also, what's the relationship between the 2237 genes and the genes in C1? In page 10, line 12-13, the author claims the CHX treatment enhances the first wave of gene upregulation that is supposed to be the C1 cluster (Fig. 1D), but the C1 cluster only has 316 genes. Please clarify these discrepancies.

We performed additional control experiments to confirm the obtained results regarding translation inhibition. We focused on the 6h imbibition time point, as at this time point, CHX treatment led to decreases and increases in mRNA production for specific gene groups. Initially, we validated changes in new mRNA levels at 6 hours of CHX treatment using RT-qPCR. Although RT-qPCR is generally noisier than RNA-seq due to reliance on a single reference gene rather than comprehensive normalisation across all transcripts, we observed that most tested genes showed changes consistent with 3'RNA-seq (Fig. S3J). We then repeated the 3'RNA-seq for new and total mRNA at 6 hours of CHX treatment, obtaining results highly consistent with the initial 3'RNA-seq experiment (Fig. S3F-I). This experiment primarily served as a control for puromycin treatment, another translation inhibitor with a different mechanism of action, and yielded results consistent with both CHX treatments (Fig. S3F-I). The differences in the effects of the two inhibitors may be explained by their distinct modes of action and distinct effects on mRNA stability (Kramer, 2017). These experiments confirm the reproducibility of our findings.

Translation inhibition with CHX was reported to cause mRNA stabilisation (Kramer, 2017). We aimed to exclude the possibility that a global shift in mRNA levels influenced normalisation and differential gene expression analysis after CHX treatment. We reanalysed the initial 3' RNA-seq data for cycloheximide and puromycin treatments, normalising them using added ERCC spike-ins. The results closely matched those from the original data, indicating no substantial genome-wide changes in total mRNA levels (Fig. S3C and F). However, a closer examination of volcano plots for both ERCC-normalised CHX treatments suggests that many mRNAs are upregulated, but mostly do not exceed a log2FC threshold of 1 (Fig. S3C and F). This may suggest some effect on mRNA stability. Accurately measuring mRNA half-lives during seed imbibition is challenging because the mRNA pool and transcriptional profiles change dramatically. Therefore, we directly compared seeds imbibed with α-amanitin to those incubated with both α-amanitin and CHX (Fig. S3K). Differential comparison suggested upregulation of a large group of total mRNAs in response to CHX treatment (Fig. S3L-M), and these mRNAs also tend to be upregulated in CHX treatments without α-amanitin (Fig. S9I).

These observations may be in line with mRNA stabilisation leading to greater detection of new mRNA after CHX treatment (Fig. S3N). The simplest explanation is that increased total mRNA is non-specifically bound by the beads and that is misinterpreted as increased transcription. We showed,

using labelled spike-ins and conversions, that the enrichment of newly transcribed mRNAs is mostly specific (Fig. 2 and S2). Therefore, the effect of nonspecific binding of total mRNAs should be more prominent for mRNAs with lower enrichment levels, due to a larger impact from nonspecific mRNAs. Notably, this is not observed (Fig. S9J). Another possibility is that the binding is specific as expected, but CHX treatment also stabilises newly synthesised 4-SU-labelled mRNAs. Newly made mRNAs in seeds may be more stable than older ones, which could accumulate damage during dry storage. Testing this is difficult because it would require labelling with 4-SU, then washing out the nucleotide or applying an inhibitor, which would be ineffective in already imbibed seeds. These scenarios suggest that the increased detection of new mRNAs after CHX treatment may reflect not only transcription but also increased stability. However, it's also possible that the inhibition of new mRNA synthesis by α-amanitin is delayed or incomplete (Fig. S9C and 1E), meaning the higher detection could reflect genuinely new mRNAs with increased transcription due to CHX treatment. We cannot definitively distinguish among these possibilities, and they likely all contribute to the observed increase in new mRNA detection following CHX, to varying degrees. Therefore, we no longer claim that CHX induces increased transcription, but rather that it leads to higher detection of some new mRNAs, partly due to their stabilisation.
It is crucial to emphasise that our second claim—that the translation block hinders the transcription of other gene groups—remains valid. Similar observations have been noted in the inflammatory response (Yamazaki and Takeshige, 2008). During inflammation, the initial responsive mRNAs are stabilised by translation inhibition, preventing the transcriptional activation of the subsequent wave. This precisely mirrors the seed's response to water uptake in the presence of CHX.
We did not claim a direct one-to-one link between new mRNAs showing differential accumulation after CHX treatment and the clusters in Fig 1. We found that a significant portion of genes producing imbibition-affected new mRNAs are among those whose levels are influenced by CHX. To strengthen our findings, we conducted a statistical analysis that confirmed these results (Fig. 3E). In the clustering analysis in Fig. 1, we used a 2-fold change cutoff, including only genes with substantial expression changes. Many genes with statistically significant changes did not meet this threshold. Additionally, some genes with minor changes that do not qualify as differentially expressed may still show specific fluctuations. To assess whether these additional mRNAs affected by CHX treatment fit existing clusters, we repeated gene module identification with WGCNA, using the CHX-affected new mRNAs as input. This analysis did not reveal new profiles of change but did allow the assignment of additional genes to existing clusters (Fig. 3E). This further showed that new mRNAs with increased levels due to CHX treatment tend to be produced early during imbibition, and new mRNAs with decreased levels tend to be produced later during imbibition.

8. Page 10, lines 1 to 6: It is difficult to read and understand the rationale behind this part. Please specify what is plotted in Fig. S3E. In addition, I'm not convinced that this data proves the claim that newly produced proteins in 3hrs are necessary for the RNA upregulation in 6hrs. A similar argument is being mentioned in the summary, which should be substantiated with more evidence.

We apologise for the lack of clarity. We included this explanation to avoid confusion about CHX's mode of action at the new mRNA level. It is important to stress that new mRNAs downregulated and upregulated by CHX treatment are produced early during imbibition, both under mock and CHX conditions. In mock-treated samples, CHX-upregulated mRNAs do not change expression between 3h and 6h, while CHX-downregulated mRNAs show increased levels of new mRNAs. During CHX treatment, CHX-upregulated mRNAs show very high new mRNA levels, while the main difference is only a weak increase in CHX-downregulated genes between the 3h and 6h time points. The plot S3E is supposed to show exactly that. These observations are confirmed by the overlap with C1-C2

clusters described in the previous point (Fig. 3E). We clarified this in the new version of the manuscript.
We agree that blocking translation does not necessarily indicate that newly produced proteins activate genes related to germination. An alternative explanation is that translation is required for quality control and damage repair during recovery from the dry stage. That is why we decided to remove those strong statements and discuss these possibilities.

9. Page 12, line 26-28: Here, the author mentioned that some upregulation of mRNA is not due to active transcription, while it seems the author assumed that C1 and C2 clusters (Fig. 1D) are mainly due to transcriptional change. This is one of the impressions I left throughout the manuscript; there are lots of arguments/discussion-like sentences, but the evidence supporting them is often complicated or indirect, which reduces the readership of this manuscript. I suggest the author re-polish the manuscript and strive for clarity in the results section.

We apologise for the lack of clarity. We proved that the C1 and C2 clusters are due to transcriptional activity, as their upregulation is almost completely blocked by α-amanitin treatment (Fig. 1D-E). This is also supported by the fact that these genes exhibit high enrichment for newly synthesised mRNAs (Fig 3A). We acknowledge that our claim that total mRNA upregulation in seeds imbibed at 4°C, compared to 22°C, results from impaired degradation is indirect. This arose from contradictory observations: upregulation of mRNAs in total mRNA (Fig. S4B-C) and an almost complete lack of new mRNA production at 4°C (Fig. 4B). Importantly, the upregulated mRNAs are enriched among those downregulated during imbibition under standard conditions (Fig. S4D). We agree that this is not an important observation. For transparency, we decided to show the effects of temperature treatment not only for new but also for total mRNAs. In our opinion, leaving this discrepancy unexplained would decrease confidence in our results. However, we believe that experimental evidence supporting our explanation is beyond the scope of this work.

10. In Fig. 6A, the authors performed Pol II ChIP-seq analysis on dry seeds and imbibed seeds. An important comparison missing here is between the ChIP-seq signal and the new RNA identified by the author through 4-sU RNA-seq. This provides an opportunity to validate if the new RNA identified is indeed due to Pol II transcription. Also, the results in Fig. s7g seem to contradict those in Fig. 2A-C, as in 6 hours, the new RNA group detected in 1 hour displayed the highest level of Pol II, while the new RNA group detected in 6 hours showed almost no Pol II. These observations further my concerns related to the robustness of the new RNA detected. I would expect the Pol II profile change to occur before or at least in parallel with the new RNA change.

We are grateful for this remark. There is an important technical difference between ChIP and labelling experiments, arising from the requirement for the amount of starting material. For labelling and inhibitor experiments, we performed seed nicking to enhance uptake, and only a few hundred seeds are required per biological replicate. However, the ChIP protocol requires several thousand seeds per replicate, which is why manually nicked seeds were not feasible. Seed nicking, by facilitating water uptake, speeds up processes induced by imbibition. That is why there is a delay in intact seeds used for ChIP compared to those used for labelling experiments. That explanation was added to the new version of the manuscript. Moreover, as the Reviewer pointed out earlier and as we explained in detail, the identification of new mRNAs using the sensitive enrichment method is largely qualitative and does not provide information on the absolute amounts of new mRNAs. This sensitivity and delayed imbibition explain why mRNAs showing enrichment at 6h in new mRNAs do not exhibit high Pol II binding to their genes. To obtain more quantitative labelling results, we used a less sensitive conversion from total mRNA. It is important to emphasise that, due to technical and biological reasons, the low efficiency of conversion makes it difficult to use it for calling new mRNAs.

Nevertheless, we binned genes based on the proportion of converted reads in their mRNAs (after correcting for conversion levels in the negative control). A high proportion of converted reads may arise from early transcription restart and/or high transcription levels. As expected, a high proportion of reads with conversion (>5%) corresponds to high Pol II binding, and a decreasing proportion is associated with lower Pol II enrichment. We replaced the old figure based on enrichment levels with the new one based on conversion in the new version of the manuscript (Fig. S7G). Together with the Pol II binding for gene clusters with different mRNA profiles along imbibition (Fig. S7H bottom), this shows that Pol II enrichment confirms the ordered restart of transcriptional activity.

Minor point:

1. Please indicate the duration of α-amanitin treatment in the main text (Page 3, lines 21-22).

That was 6h. This was corrected.

2. Page 3, Line 35, what is the judgment that the selected "stable mRNAs" are actually stable, especially during the early seed imbibition stage?

This was described in detail in Supplemental Bioinformatic Methods and is now included in the main body of the manuscript. We initially planned to use yeast mRNAs for normalisation, as our library was spiked with total yeast RNA; however, the yeast counts proved unreliable for normalisation (described in Supplemental Bioinformatic Methods). We then used the strategy described by (Sorenson *et al.*, 2018), based on the observation that stable mRNAs tend to increase their levels over the time course of transcription inhibition. Importantly, this criterion alone is insufficient, as it assumes the Pol II transcription profiles do not change and Pol II activity is completely shut down by the inhibitor. During seed imbibition, we observed a strong restart of Pol II activity and shifts in gene expression. In addition, seeds are compact, delaying the penetration of the α-amanitin, which allows for some initial upregulation even in the presence of the inhibitor (Fig. 1 – C1 cluster, Supplemental Bioinformatic Methods). Including such mRNAs in the normalisation set would affect the detection of mRNA-level changes during mock imbibition. That is why we also used another criterion in selecting mRNAs for the normalisation set: they should not change their levels during imbibition under standard conditions. mRNAs that combined those characteristics (Dataset S1) were used to renormalise the entire data set before differential analysis.

3. Page 6 line 11, two peaks of mRNA upregulation, please clarify in the main text the relationship between this upregulation and transcription.

The C1 and C2 clusters categorise genes that are upregulated during seed imbibition. C1 genes are upregulated earlier and stop changing their expression in the later stages, while C2 genes show increased expression during the later phase of imbibition. Importantly, the upregulation of all these genes is strongly inhibited by α-amanitin, confirming that it is driven by new mRNA production. This explanation is now part of the new manuscript version.

4. Please briefly explain the selection of 4tU over 4sU for labelling the newly synthesised RNA.

We apologise for the confusion. As Reviewer 1 also pointed out, this was our mistake. Throughout the experiments, we consistently used 4-SU (4-thiouridine) rather than 4-tU (4-thiouracil). We have now corrected this and applied the proper nomenclature throughout the manuscript. Both compounds must first be modified by endogenous enzymes into UMP analogues before being further phosphorylated into UTP analogues, which allows them to be used by polymerases. This process may be relatively slow, especially during seed imbibition, when metabolism only restarts. There is also some nomenclature confusion, but 4-tU was used in plants to enrich for new RNAs

(Sidaway-Lee *et al.*, 2014; Doidy *et al.*, 2016). Nevertheless, its incorporation into RNAs seems relatively slow in intact plants (Sidaway-Lee *et al.*, 2014). As we were interested in the first instance of new transcription, we thought that using a modified nucleoside rather than a nucleobase would facilitate quicker incorporation of the modified nucleotide into new mRNAs.

5. The authors performed multiple batches of 4tU–labeled RNA-seq at 3 hours under different conditions. How well do these replicates agree with each other?

We apologise for not mentioning this analysis in the manuscript; these data are presented in Figure S9D-F and commented on in the Supplemental Bioinformatic Methods.

We would like to thank the Reviewer for asking important questions about the specificity of our assays, which helped us to improve our work.

References:

Bai, B. *et al.* (2018) ‘Combined transcriptome and translatome analyses reveal a role for tryptophan-dependent auxin biosynthesis in the control of *DOG1* -dependent seed dormancy’, *New Phytologist*, 217(3), pp. 1077–1085. Available at: https://doi.org/10.1111/nph.14885.

Barrass, J.D. *et al.* (2015) ‘Transcriptome-wide RNA processing kinetics revealed using extremely short 4tU labeling’, *Genome Biology*, 16(1), p. 282. Available at: https://doi.org/10.1186/s13059-015-0848-1.

Berg, K. *et al.* (2024) ‘Correcting 4sU induced quantification bias in nucleotide conversion RNA-seq data’, *Nucleic Acids Research*, 52(7), pp. e35–e35. Available at: https://doi.org/10.1093/nar/gkae120.

Doidy, J. *et al.* (2016) ‘“Hit-and-Run” transcription: de novo transcription initiated by a transient bZIP1 “hit” persists after the “run”’, *BMC Genomics*, 17(1), p. 92. Available at: https://doi.org/10.1186/s12864-016-2410-2.

Doumane, M. *et al.* (2021) ‘Inducible depletion of PI(4,5)P2 by the synthetic iDePP system in Arabidopsis’, *Nature Plants*, 7(5), pp. 587–597. Available at: https://doi.org/10.1038/s41477-021-00907-z.

Duffy, E.E., Schofield, J.A. and Simon, M.D. (2019) ‘Gaining insight into transcriptome-wide RNA population dynamics through the chemistry of 4-thiouridine’, *WIREs RNA*, 10(1), p. e1513. Available at: https://doi.org/10.1002/wrna.1513.

Herzog, V.A. *et al.* (2017) ‘Thiol-linked alkylation of RNA to assess expression dynamics’, *Nature Methods*, 14(12), pp. 1198–1204. Available at: https://doi.org/10.1038/nmeth.4435.

Kramer, S. (2017) ‘Simultaneous detection of mRNA transcription and decay intermediates by dual colour single mRNA FISH on subcellular resolution’, *Nucleic Acids Research*, 45(7), pp. e49–e49. Available at: https://doi.org/10.1093/nar/gkw1245.

Leng, X. *et al.* (2020) ‘Organismal benefits of transcription speed control at gene boundaries’, *EMBO reports*, 21(4), p. e49315. Available at: https://doi.org/10.15252/embr.201949315.

Lin, L. *et al.* (2020) ‘Normalizing single-cell RNA sequencing data with internal spike-in-like genes’, *NAR Genomics and Bioinformatics*, 2(3), p. lqaa059. Available at: https://doi.org/10.1093/nargab/lqaa059.

Meng, L. and Feldman, L. (2010) ‘A rapid TRIzol-based two-step method for DNA-free RNA extraction from *Arabidopsis* siliques and dry seeds’, *Biotechnology Journal*, 5(2), pp. 183–186. Available at: https://doi.org/10.1002/biot.200900211.

Shu, J. *et al.* (2022) ‘Transcription elongator SPT6L regulates the occupancies of the SWI2/SNF2 chromatin remodelers SYD/BRM and nucleosomes at transcription start sites in Arabidopsis’, *Nucleic Acids Research*, 50(22), pp. 12754–12767. Available at: https://doi.org/10.1093/nar/gkac1126.

Sidaway-Lee, K. *et al.* (2014) ‘Direct measurement of transcription rates reveals multiple mechanisms for configuration of the Arabidopsisambient temperature response’, *Genome Biology*, 15(3), p. R45. Available at: https://doi.org/10.1186/gb-2014-15-3-r45.

Sorenson, R.S. *et al.* (2018) ‘*Arabidopsis* mRNA decay landscape arises from specialized RNA decay substrates, decapping-mediated feedback, and redundancy’, *Proceedings of the National Academy of Sciences*, 115(7). Available at: https://doi.org/10.1073/pnas.1712312115.

Swiatkowska, A. *et al.* (2012) ‘Kinetic analysis of pre-ribosome structure in vivo’, *RNA*, 18(12), pp. 2187–2200. Available at: https://doi.org/10.1261/rna.034751.112.

Szabo, E.X. *et al.* (2020) ‘Metabolic Labeling of RNAs Uncovers Hidden Features and Dynamics of the Arabidopsis Transcriptome’, *The Plant Cell*, 32(4), pp. 871–887. Available at: https://doi.org/10.1105/tpc.19.00214.

Yamazaki, S. and Takeshige, K. (2008) ‘Protein synthesis inhibitors enhance the expression of mRNAs for early inducible inflammatory genes via mRNA stabilization’, *Biochimica et Biophysica Acta (BBA) - Gene Regulatory Mechanisms*, 1779(2), pp. 108–114. Available at: https://doi.org/10.1016/j.bbagrm.2007.11.001.

Zhang, Yu *et al.* (2026) ‘A metrological foundation for absolute transcriptomics using International System of Units-anchored calibrators’, *Nature Communications*, 17(1), p. 2747. Available at: https://doi.org/10.1038/s41467-026-70582-1.

Reviewer #1

In this revision, the authors have addressed most of my concerns raised last time. I appreciate the inputs provided here and think it would be a valuable resource for 4sU labelling methodology, as the authors have included more control experiments this time. It is a good idea to have both unlabeled and labeled spike for nonspecific binding control and normalization reference. And I am almost convinced that the new transcription is robust and essential for seed germination. Nevertheless, I find it difficult to read due to the complex nature of this new methodology establishment and the lack of clarity in some data presentations. I would like to see a clearer and more precise description of the data processing, especially normalization and new RNA calling, as well as the underlying logic.

We appreciate the Reviewer's feedback and comments that help us improve our manuscript. We have made significant efforts to enhance the clarity of our methodological descriptions and to clarify the reasoning behind our data-processing approaches. Specifically, we reorganised the manuscript to group analyses that confirm the quality of our labelling method separately from those reporting experimental outcomes. Simultaneously, we aimed to adhere to the manuscript length restrictions as closely as possible, which is why some explanations are still included in the Supplementary Methods.

The authors note that non-specific binding of biotinylated RNA to streptavidin beads constitutes a major technical challenge in 4sU labeling assays. To assess background noise, the use of exogenous spike-in RNA is indeed a recommended practice. However, the authors simultaneously employ the same spike-in RNA for normalization—a strategy that may introduce systematic bias. Specifically, if non-specific binding varies across samples, the residual unlabeled spike-in RNA remaining after streptavidin bead purification will likewise vary, rendering it an unreliable internal reference for normalization. An alternative approach—using only the labeled fraction of spike-in RNA as the normalization standard, however challenges associated with mRNA quantification in RNA isolated from dry versus imbibed seeds remains. The supplementary methods indicate that the proposed normalization against an internal standard; revisiting this approach may offer greater robustness. In the case of B. napus, I cannot understand the logic for normalization.

Thank you for raising these important doubts. We completely agree with the Reviewer that using identified internal control transcripts rather than external spike-ins may, in certain cases, enhance the robustness and reproducibility of labelling results. In fact, we now compare the two experiments using two different normalisation methods: the ERCC spike-ins (as presented in Supplementary Fig. 2g) and endogenous non-transcribed mRNAs (Supplementary Fig. 9h; described in the Supplementary Bioinformatics Methods). For 1h labelling, internal controls allow for greater robustness; for 3h, when strong transcription is already ongoing, switching between internal and external controls does not change much (Supplementary Fig. 2g and 9h). This is most likely due to the mentioned difficulty in adding exactly the same proportion of external spike-ins to an RNA sample from seeds. As we are not leaning toward identifying each specific new mRNA but rather to pinpoint the extent of new transcription, we decided to stay with external controls. In our opinion, their use is logically clear and makes our approach simpler to explain. The identification of internal controls is much easier when external spike-ins are available, and, in our opinion, the relation between internal and external controls is largely recursive in such a case.
The sample normalisation in *B. napus* experiments best illustrates the effort required to identify internal control transcripts when external spike-ins are unavailable. We therefore had to rely on observations from early in our project, where different data normalisation methods were tested. By performing differential analysis of new versus input mRNAs using standard normalisation, we found that ERCC spike-ins and internal mRNAs, which were not newly produced, were strongly

downregulated. This occurs because new mRNAs are enriched, while non-transcribed RNAs are depleted in labelled, enriched samples. Using this insight, we analysed *B. napus* samples and identified a set of endogenous mRNAs that were heavily downregulated (new vs input at two different time points). These transcripts were then used to renormalise the *B. napus* dataset and to identify genes that produce new mRNAs. Identified sets of genes that produce and do not produce new mRNAs showed high concordance with those from Arabidopsis identified using ERCC spike-ins for normalisation (Fig. 5 and Supplementary Fig. 6). In addition, when we normalised *B. napus* data using homologs of *A. thaliana* endogenous control genes, we obtained a very similar set of new transcripts. In the new version of the Supplementary Bioinformatics Methods, we added a comparison showing that identifying new mRNAs using "downregulated mRNAs" normalisation and "non-transcribed homologues" normalisation yields highly consistent results (Supplementary Fig. 9j). We have also rewritten the corresponding section of the Supplementary Bioinformatics Methods to clarify it.

Figure S2g: Does the grey shading denote mRNAs that are not newly synthesized during imbibition? The criterion used to classify transcripts as "new" versus "pre-existing" is not explicitly defined. Notably, transcripts annotated as "not new" exhibit relatively high C-to-T conversion ratios (e.g., up to 2.5%), which challenges the biological interpretability of this classification. It remains unclear whether a quantitative threshold—such as a minimum C-to-T ratio—or a statistically rigorous approach (e.g., significance testing against background mutation rate or technical noise) was applied to support the distinction. In contrast, Figure 2f adopts a more straightforward definition: any transcript bearing at least one T-to-C mutation is designated as "newly synthesized." If the Fig2f and FigS2g depict the same two transcript categories (new and no), their reported C-to-T conversion ratios appear inconsistent, raising questions about methodological coherence or data interpretation.

Yes, the grey colour indicates genes that, in this particular 3-hour imbibition experiment, were not found to produce new mRNAs. Genes with new transcripts were identified using the previously described enrichment criteria, which are now clearly referenced in the figure description. This means that the identification of genes producing new mRNAs is orthogonal to the conversion method, which was used to validate the enrichment approach rather than as an independent method for calling genes with new mRNAs. In our enrichment approach, we rely on two criteria: high enrichment over the ERCC-defined baseline and statistical significance related to the differences between replicates. It is possible that some relatively highly labelled mRNAs do not meet these criteria, as indicated by the orthogonal conversion method (example pointed by the Reviewer). Under no circumstances were conversion levels (neither conversion ratios nor ratios of reads with conversions) used to call genes producing new mRNA. After careful consideration, we decided that conversion events are too sparse to support robust statistical analysis. This is now more clearly stated in the manuscript. Figures 2f and S2g (now 2e and S2d) depict the same transcripts identified in the same experiment. Those figures simply present two ways to represent the conversion events, while also highlighting their sparsity. Figure S2g (now S2d) shows the average frequency of each individual U in the transcript being converted, and such events are relatively rare even for highly produced new mRNAs (Herzog *et al.*, 2017). Figure 2f (now 2e) shows the frequency of the reads that contain any conversion event, thus raising apparent detection of rare conversions. That distinction is now more clearly explained in the manuscript.

Line 599 What is the exact amount of ERCC RNA added to the biotinylated total RNA.

The producer does not specify the combined concentration of ERCC RNAs (Thermo Fisher Scientific ERCC RNA Spike-In Mix, 4456740). However, based on the file containing individual concentrations of 92 ERCC RNAs, we estimated the stock concentration to be around 100 nM.

I recommend relocating the description of normalization procedures for both control and T-to-C–converted samples in 3′ RNA-seq data from the Supplementary Bioinformatics section to the main Methods section, to improve accessibility and methodological transparency for readers. Apparently, more details are needed.

Thank you for your suggestion. We have now provided a streamlined and detailed description of our labelling method and supporting analysis in the main manuscript. However, we also need to stay within the required manuscript length limit, so we decided to place some detailed information in the Supplementary Bioinformatics Methods rather than moving it to the main Methods. The advantage of this approach is that it allows a more natural flow when describing the additional analysis and the logic behind it. We hope this compromise is acceptable and that we have at least partially succeeded in explaining the details of our work in the main text.

Reviewer #2

In this revised version, the authors have addressed the concerns I raised previously. In particular, they estimated nascent RNA levels by incorporating both ERCC controls and 4sU-labeled spike-ins. With these controls, the data are much more convincing.

We are grateful for the helpful comments and for the appreciation of our efforts.

I have only one remaining suggestion. The authors show that the conversion ratio reaches approximately 40% of reads after enrichment. Although this is an impressive level of enrichment, it also implies that around 60% of the sequenced reads remain unconverted and may represent background. Under these conditions, the authors should clarify how they determine that the remaining unconverted reads do not lead to an overestimation of newly synthesized RNA. Relatedly, why not restrict the analysis to converted reads only? This point should be clarified in the revised manuscript before publication.

Thank you for raising this issue. The conversion served as an orthogonal approach to validate our enrichment method of calling new mRNAs. After careful consideration, we decided not to rely on it for new mRNA identification due to its scarcity, which would limit the robustness of the statistical analysis. Additionally, the setup specified by the Reviewer, which uses enrichment followed by conversion-event identification to subset high-confidence new mRNAs, has several limitations. As described in the new version of the manuscript: *"(…) level of conversion in enriched mRNAs is most likely still an underestimation, as: 1) 3'RNA-seq samples only the transcript's 3' end, while labelling that enabled enrichment may occur in other parts of mRNA. 2) Conversion efficiency in our experiment may be relatively low, as exemplified by our fully labelled spike-in (25-44%; Supplementary Fig. 2d). For these reasons, and because of its low per-transcript sensitivity, we use enrichment rather than conversion as our primary criterion for calling new mRNAs."* Especially low conversion efficiency may mean that even 75% of the labelled U remains unconverted. Given the sparsity of 4-SU incorporation, excluding unconverted reads from available experiments may not be a good strategy. The conversion-based new mRNA calling would require performing a new redesigned experiment to ensure full coverage and conversion of labelled mRNAs.

Herzog, V.A. *et al.* (2017) 'Thiol-linked alkylation of RNA to assess expression dynamics', *Nature Methods*, 14(12), pp. 1198–1204. Available at: https://doi.org/10.1038/nmeth.4435.

Reviewer #1

In this revision, the authors have clarified the normalization methodology, which incorporated both external and internal reference standards, and demonstrated that both approaches yield consistent conclusions. This comparative analysis constitutes a valuable contribution to the field and enhances the methodological rigor of the study. Furthermore, the authors have refined the descriptions of the experimental procedures and bioinformatic analyses, improving clarity and reproducibility. Having addressed all prior concerns, I have no further substantive questions. However, I recommend that the Discussion section include a concise summary of the key methodological aspects and their implications for interpretation and future applications.

We are grateful for the positive evaluation of our work. We added a short summary of the methodology to the Discussion section, as suggested.
