# Supplementary material for: Transcriptional activity during seed imbibition is conserved, robust and required for germination
Source: Nat Commun. 2026 Aug 19;17:9916. doi: 10.1038/s41467-026-76795-8 (PMC13582861; doi:10.1038/s41467-026-76795-8)
Supplement: Supplementary file 2 [file 41467_2026_76795_MOESM1_ESM.pdf]

Transcriptional activity during seed imbibition is conserved, robust and required for germination

Michal Krzyszton, Veena Halale Manjunath, Lien Brzezniak, Sargam Bharti, Martyna Katarzyna Adach, Silviya S. Lal, Patrycja Witek, Dariusz J. Smolinski, Szymon Swiezewski

Supplementary Bioinformatic Methods

Supplementary Figure 1. Scarification allows analysis of Pol II transcription during seed imbibition.

Supplementary Figure 2**.** Widespread production of new mRNAs during seed imbibition.

Supplementary Figure 3**.** Effects of cycloheximide on new mRNA levels during imbibition.

Supplementary Figure 4**.** The early new mRNA production phase during seed imbibition is robust.

Supplementary Figure 5**.** Seed maturation temperature has a minor effect on mRNA production during imbibition.

Supplementary Figure 6**.** The pattern of new transcription is conserved during *B. napus* imbibition.

Supplementary Figure 7**.** Pol II binding in dry seeds reflects the gene expression patterns associated with seed maturation.

Supplementary Figure 8**.** smFISH analysis confirms limited production of mRNAs for a gene with a Pol II peak specific to dry seeds.

Supplementary Figure 9**.** Additional bioinformatic analyses.

Supplementary Table 1**.** Primer list.

Supplementary Table 2**.** 3’RNA-seq statistics.

Supplementary Table 3**.** smFISH probes list.

Supplementary Bioinformatic Methods

α-amanitin data normalisation

Transcription inhibition experiments with α-amanitin were supplemented with total yeast RNA (5% of the Arabidopsis RNA amount) for subsequent normalisation. However, due to unreliable measurements of RNA concentrations, particularly in dry seed samples compared to imbibed ones, the levels of yeast mRNAs proved too variable to serve as a normalising factor (Supplementary Fig. 9a). As an alternative, we identified a group of stable mRNAs (Dataset S1) for normalisation, as previously described (Sorenson *et al.*, 2018; Viegas *et al.*, 2023). These were chosen after an initial naïve normalisation of the dataset using default DESeq2 normalisation. Selection was based on two assumptions. 1) The stable mRNA content should increase during imbibition in α-amanitin, as their relative amounts in the library rise due to the degradation of other mRNAs (Sorenson *et al.*, 2018; Viegas *et al.*, 2023). To assess the increase during imbibition due to α-amanitin treatment, we compared α-amanitin- and mock-treated seeds at the 6 h and 12 h time points and identified genes with upregulated expression (DESeq2; padj < 0.05, log2FC > 0, altHypothesis = "greater"). In the referenced studies, the only expected changes in mRNA levels during treatment were due to complete or partial transcriptional arrest. Since seed imbibition is a dynamic process, the inhibitor must counteract the rapid, strong increase in gene expression. The observed gene upregulation with α-amanitin treatment might be more easily observed if the gene expression is highly and early upregulated during imbibition and if α-amanitin has not yet fully blocked transcription. Because the selected genes will be used to normalise both mock and α-amanitin time-course samples, it is essential to exclude imbibition-induced genes from the normalisation set. Otherwise, they may lead to an underestimation of gene induction during imbibition and completely mask any possible inefficiency in transcriptional inhibition by α-amanitin. That is why: 2) Stable mRNAs should remain unchanged during imbibition in the mock-treated seeds. To identify mRNAs that remain unchanged during mock imbibition, we compared dry seeds with seeds imbibed for 6 h and 12 h (DESeq2; padj < 0.05, $|\log2FC| < \log2(1.5)$, altHypothesis = "lessAbs"). For the final list of stable mRNAs used for normalisation of that data set, selected mRNAs should show increased expression upon α-amanitin treatment and lack of expression change due to imbibition. All these steps are documented on the GitHub website with the code used for figure preparation. Notably, when averaged across biological replicates, CPM- and stable gene normalisation yielded globally consistent results, indicating that our normalisation strategy did not bias the outcomes (Supplementary Fig. 9b).

Later, we performed mRNA stability analysis comparing α-amanitin-treated mock-treated seeds with dry seeds, using ERCC spike-in normalisation (Supplementary Fig. 3k,m). In principle, we could use the mRNAs identified as stable in this experiment to normalise our dataset. We tested a set of mRNAs that do not show strong expression changes between 6 h α-amanitin-imbibed seeds compared to dry seeds (DESeq2; padj $<$ 0.05, $|\log2FC| < 1$, altHypothesis = "lessAbs"). However, while some α-amanitin effects may be observed as early as 1 h into the treatment (Supplementary Fig. 1d), we observed that the stable mRNAs identified here tend to be upregulated during standard, inhibitor-free imbibition (Supplementary Fig. 9c). This disqualifies them as a normalisation factor, as explained above. This also indicates that although α-amanitin-driven inhibition of Pol II activity is observed early on, it is not complete.

New mRNA identification after 4-SU labelling and enrichment

Detection of mRNA among transcripts bound by the beads during the enrichment step cannot be considered proof that it is newly produced, as unspecific binding is a major problem in new transcript enrichment analysis (Duffy, Schofield and Simon, 2019). In most experiments aimed at identifying newly produced mRNA, we introduced unlabelled ERCC spike-ins prior to RNA biotinylation to estimate the pulldown's specificity. As expected, ERCC spike-ins were detected in input samples as well as, to lower levels, in enriched samples. This established a baseline for determining whether the new mRNAs are truly enriched by binding to the beads. New mRNAs were identified as those showing significant upregulation when comparing new mRNA-enriched and input samples using unlabelled ERCC spike-ins for normalisation (DESeq2; padj < 0.05; log2FC > 2). Using this approach, we ensure that any mRNA identified as a new transcript exhibits much higher binding to the beads than the negative control. Additionally, we filter out mRNAs with low read counts in count matrices, requiring at least as many reads as there are biological replicates. Importantly, our method and analysis pipeline yield highly consistent results across independent experiments aimed at new mRNA enrichment (Supplementary Fig. 9 d,e,f).

We found that externally added spike-ins can be replaced by a set of mRNAs identified as not being transcribed during early imbibition (Supplementary Fig. 2n,o.p; Supplementary Data 2). A direct comparison

of enrichment estimates using ERCC spike-ins and non-transcribed genes produced almost identical results (Supplementary Fig. 9g), and the outcomes from these two normalisation methods show high similarity (Supplementary Fig. 9d,e). This indicates that this alternative normalisation approach can effectively replace ERCCs in experiments where they are absent (Fig. 4 – ABA and ANT treatments, seed maturation temperatures). We also tried to assess whether internal controls might be more robust than external spike-ins when identifying new mRNAs. For this, we renormalised two experiments (Fig. 2b,c and Supplementary Fig. 2f) using mRNAs identified as not transcribed during early imbibition and compared the outcomes in terms of the genes that produce new mRNAs (see Supplementary Fig. 2g and 9h). At the 3 h time point, with strongly active new mRNA production, alternative approaches to normalisation do not change much. However, at 1h, with still-weak transcriptional activity, internal normalisation increased both robustness and reproducibility between experiments. This is most likely due to the noted difficulty in adding exactly the same proportion of external spike-ins to an RNA sample from seeds (Supplementary Fig. 9a), which is more significant for samples with low detection levels.

The *B. napus* imbibition experiment (Fig. 5) illustrates the challenge of finding an internal normalisation reference when external spike-ins are unavailable — in this case, because technical issues prevented the usage of ERCC spike-ins. As described above, new mRNAs are identified by comparing enriched and input samples, normalised against a set of transcripts known not to be newly transcribed (behaving as unlabelled spike-ins). For Arabidopsis, this reference set was identified directly from ERCC-normalised data, as present in input samples that showed no enrichment after pulldown (Supplementary Fig. 2n). Without ERCC spike-ins, this direct identification was not possible for *B. napus*. To get around this, we drew on an observation made during a comparison of different normalisation strategies. When enriched and input samples are compared using standard normalisation (all transcript set rather than on a defined non-transcribed set), transcripts that are not newly transcribed appear downregulated in the enriched sample (Supplementary Fig. 9i). This happens because newly synthesised transcripts make up a substantial share of the enriched sample's signal, and non-specifically bound transcripts, including ERCC spike-ins, are depleted. This gave us a way to define a *B. napus*-specific reference set without spike-ins: we performed a standard-normalisation comparison of enriched versus input samples at two imbibition time points and selected the most strongly downregulated transcripts as our reference set, then used this set to renormalise the *B. napus* data and call new mRNAs. Based on homologous analysis, the resulting *B. napus* new mRNA set was highly concordant with the corresponding Arabidopsis set, indicating that this strategy recovers a biologically meaningful normalisation reference even without spike-in controls (Fig. 5; Supplementary Fig. 6). To validate this approach further, we compared the new mRNA sets obtained using this data-derived "downregulated transcripts" reference with those obtained using *B. napus* homologues of the Arabidopsis non-transcribed transcript set ("non-transcribed homologues" normalisation). The two approaches gave highly consistent results (Supplementary Fig. 9j).

New mRNA differential production analysis

We demonstrated, using various methods, that mRNAs attached to beads are significantly enriched in newly synthesised mRNAs (Fig. 2 and Supplementary Fig. 2), albeit not free from non-specifically bound total mRNAs. A key concern is whether variations in total mRNA levels might affect the detection of differentially produced new mRNAs. Generally, the impact of nonspecific total mRNA binding is minimised by normalising with unlabelled ERCC spike-ins during differential analysis, as this excludes mRNAs that are enriched to levels similar to those of ERCC. A clear counterpoint is provided by the analysis of new mRNA production at different imbibition temperatures (Supplementary Fig. 4). When comparing 4°C with 22°C imbibed seeds, some mRNAs accumulate in the total fraction at the lower temperature; however, they are not detected as new mRNAs in our enrichment analysis. Their accumulation in the total fraction likely results solely from mRNA stabilisation rather than from new mRNA production. On the other hand, in our imbibition time-course experiments, we detect many mRNAs as newly produced. During the observed widespread restart of transcription in imbibed seeds, it is actually expected that new mRNA levels should, at least to some extent, be reflected in total mRNA levels. Such a correlation makes it difficult to rule out effects related to nonspecific binding to the beads. This is perhaps best illustrated by analysing new mRNA production during CHX treatment (Fig. 3). We do not observe extensive changes in total mRNA levels after 6 hours of CHX treatment compared to mock (Supplementary Fig. 3c,f). However, combined CHX and α-amanitin treatment may indicate widespread mRNA stabilisation (Supplementary Fig. 3k,m). A closer examination of both CHX treatments without α-amanitin showed that these mRNAs tend to be upregulated, but mostly do not exceed a log2FC threshold

of 1 (Supplementary Fig. 9k). It could suggest that stabilisation contributes to increased detection of new mRNA following CHX treatment (Supplementary Fig. 3n), perhaps due to nonspecific binding. Alternatively, new mRNAs may be stabilised even more strongly than the stored mRNAs upon CHX treatment. It is also possible that the inhibition of new mRNA synthesis by α-amanitin is delayed or incomplete (Supplementary Fig. 9c) and that the increased detection actually reflects genuine new mRNAs produced by heightened transcription due to CHX treatment. If the change in the new mRNA levels was mainly caused by nonspecific binding of total mRNAs, then affected new mRNAs should display a low level of enrichment compared to the input, allowing for a greater contribution of total nonspecific mRNAs. Notably, that is not the case (Supplementary Fig. 9l). We conclude that we cannot rule out the possibility that differences in nonspecific binding to the beads may affect the identification of some differently produced new mRNAs; however, our controls suggest that this is unlikely for most mRNAs.

Some of our experiments lacked external spike-ins that could facilitate differential analysis of new mRNA levels (Fig. 4 – ABA and ANT treatments, seed maturation temperatures). For ABA and ANT treatments, we substituted them with a set of non-transcribed mRNAs, which is justified since we use the same dry seeds for imbibition in the mock, and changes in total mRNA levels are minimal. In fact, non-transcribed mRNAs may offer improved normalisation for analysing differential mRNA production between samples, compared with externally added spike-ins (Lin *et al.*, 2020), which are as reliable as measurements of RNA levels that, in seed samples, pose challenges (Supplementary Fig. 9a). For the seed maturation experiment, this group of genes remains non-transcribed in seeds matured at different temperatures, as these mRNAs showed patterns similar to the ERCC spike-ins when comparing new and input mRNA samples (Supplementary Fig. 5b). That is why we were able to use them for the new mRNA identification. However, different maturation conditions alter the initial levels of those mRNAs, potentially affecting differential analysis. Therefore, in this instance, we included all mRNAs for normalisation but excluded from the results those few that did not show new mRNA transcription (Supplementary Fig. 5b), as their changes reflect alterations in the total rather than the new mRNA fraction (Supplementary Fig. 5c).

Duffy, E.E., Schofield, J.A. and Simon, M.D. (2019) 'Gaining insight into transcriptome-wide RNA population dynamics through the chemistry of 4-thiouridine', *WIREs RNA*, 10(1), p. e1513. Available at: https://doi.org/10.1002/wrna.1513.

Lin, L. *et al.* (2020) 'Normalizing single-cell RNA sequencing data with internal spike-in-like genes', *NAR Genomics and Bioinformatics*, 2(3), p. lqaa059. Available at: https://doi.org/10.1093/nargab/lqaa059.

Sorenson, R.S. *et al.* (2018) '*Arabidopsis* mRNA decay landscape arises from specialized RNA decay substrates, decapping-mediated feedback, and redundancy', *Proceedings of the National Academy of Sciences*, 115(7). Available at: https://doi.org/10.1073/pnas.1712312115.

Tremblay, B.J.M. *et al.* (2024) 'Interplay between coding and non-coding regulation drives the Arabidopsis seed-to-seedling transition', *Nature Communications*, 15(1), p. 1724. Available at: https://doi.org/10.1038/s41467-024-46082-5.

Viegas, J.O. *et al.* (2023) 'Calculating RNA degradation rates using large-scale normalization in mouse embryonic stem cells', *STAR Protocols*, 4(3), p. 102534. Available at: https://doi.org/10.1016/j.xpro.2023.102534.

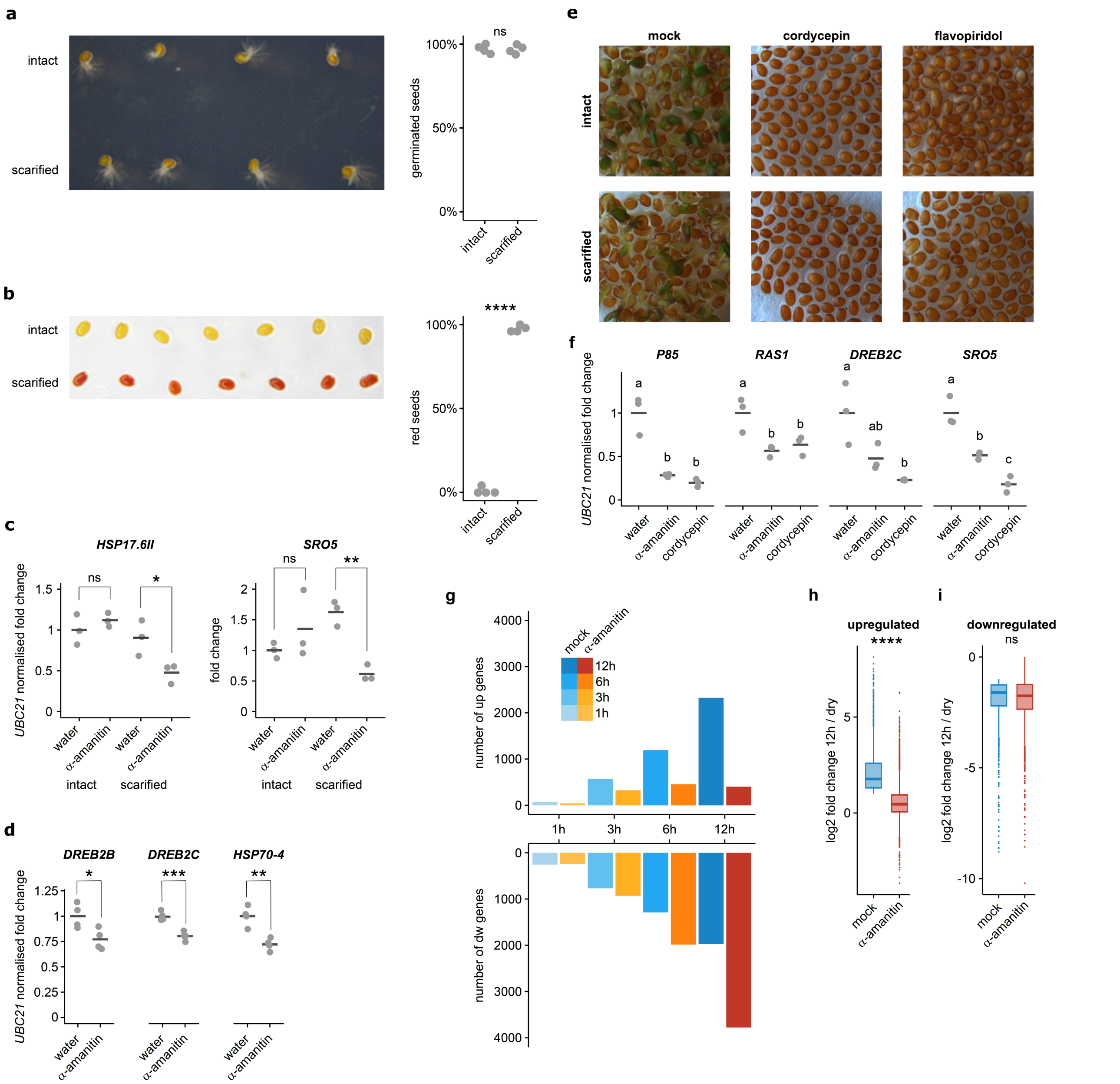

Supplementary Fig. 1. Scarification allows analysis of Pol II transcription during seed imbibition. **A)** Scarified and intact seeds were germinated. A representative picture is on the left, and quantification is on the right. **B)** Seed scarification enhances the uptake of tetrazolium red, which stains only viable seeds. A representative picture is shown on the left, and the quantification of stained seeds after overnight incubation is shown on the right. (A) and (B) n=4 biological replicates of 50 seeds each; paired two-sided t-tests (**** < 0.0001, ns – non-significant; Source Data file contains exact p-values). **C)** α-amanitin inhibits mRNA upregulation during 6 h imbibition only in scarified seeds; n=3 biological replicates. RT-qPCR analysis results were related to intact water-imbibed seeds, with *UBC21* mRNA as the reference. **D)** α-amanitin begins to inhibit mRNA regulation as early as 1 hour after the incubation of scarified seeds; n=4 biological replicates. RT-qPCR analysis results are presented relative to water-imbibed seeds, using *UBC21* mRNA as the reference. **E)** Germination phenotypes, at 3 days post-imbibition, for seeds imbibed in cordycepin, flavopiridol, and mock solutions. **F)** α-amanitin and cordycepin similarly inhibit mRNA upregulation during 6 hours of imbibition in scarified seeds; n=3 biological replicates. RT-qPCR analysis results are shown relative to water-imbibed seeds, with *UBC21* mRNA as the reference. Lines indicate the mean in (C), (D), and (F). Two-sided t-test p-value: ns - non-significant, * < 0.05, ** < 0.01, *** < 0.001; Source Data file contains exact p-values. **E)** The number of DEGs (DESeq2; selected stable genes normalisation; $|\log_2FC| > 1$, padj < 0.05) when comparing mock and α-amanitin-treated samples to dry seeds. The top plot displays genes with upregulated expression (up), while the bottom plot shows genes with downregulated (dw) expression. **H)** and **I)** Boxplots compare gene

expression fold change in dry seeds versus 12-hour imbibed seeds under mock and α-amanitin treatments for genes with upregulated (H) and downregulated (I) expression in mock (central line represents median; whiskers denote a 1.5 interquartile range; outliers are marked with dots; p-values from the two-sided t-test comparing samples;**** = 0, ns – non-significant – 0.41). A-D and F - Source data are provided as a Source Data file.

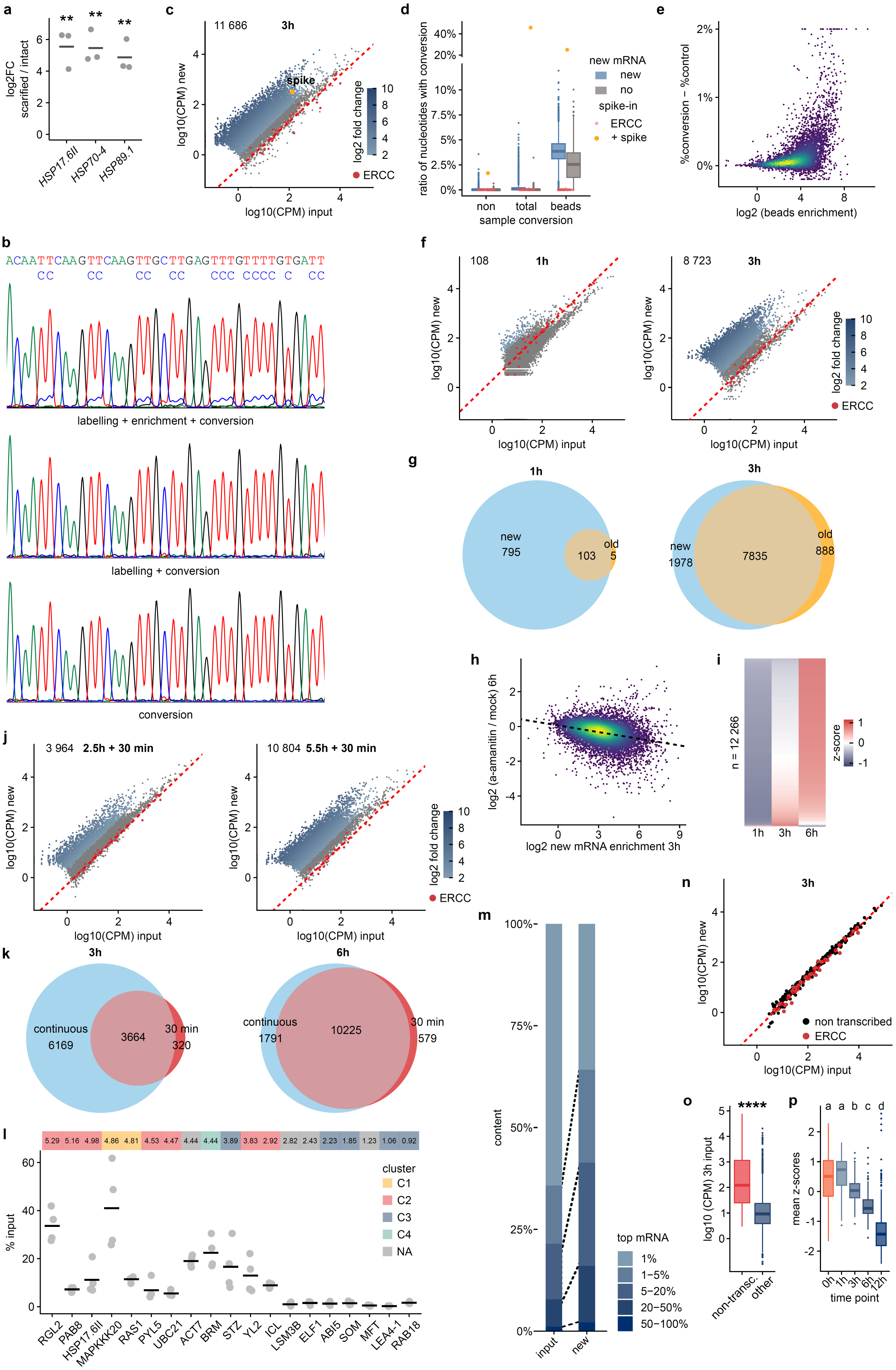

a
log2FC scarified / intact
**
HSP17.6II
HSP70-4
HSP89.1
c
11 686
3h
spike
log10(CPM) new
log10(CPM) input
log2 fold change
ERCC
d
ratio of nucleotides with conversion
40%
20%
10%
7.5%
5%
2.5%
0%
non
total
beads
sample conversion
new mRNA
new
no
spike-in
ERCC
+ spike
e
%conversion − %control
2%
1%
0%
log2 (beads enrichment)
b
ACAATTCAAGTTCAAGTTGCTTGAGTTTGTTTTGTGATT
CC CC CC CC CCC CCCC C CC
labelling + enrichment + conversion
labelling + conversion
conversion
f
108
1h
8 723
3h
log10(CPM) new
log10(CPM) input
log2 fold change
ERCC
g
1h
new
795
103
old
5
3h
new
1978
7835
old
888
h
log2 (a-amanitin / mock) 6h
log2 new mRNA enrichment 3h
i
n = 12 266
z-score
1h
3h
6h
j
3 964
2.5h + 30 min
10 804
5.5h + 30 min
log10(CPM) new
log10(CPM) input
log2 fold change
ERCC
k
3h
continuous
6169
3664
30 min
320
6h
continuous
1791
10225
30 min
579
l
5.29 5.16 4.98 4.86 4.81 4.53 4.47 4.44 4.44 3.89 3.83 2.92 2.82 2.43 2.23 1.85 1.23 1.06 0.92
% input
cluster
C1
C2
C3
C4
NA
RGL2
PAB8
HSP17.6II
MAPKKK20
RAS1
PYL5
UBC21
ACT7
BRM
STZ
YL2
ICL
LSM3B
ELF1
ABI5
SOM
MFT
LEA4-1
RAB18
m
100%
75%
50%
25%
0%
content
input
new
top mRNA
1%
1−5%
5−20%
20−50%
50−100%
n
3h
log10(CPM) new
log10(CPM) input
non transcribed
ERCC
o
****
log10 (CPM) 3h input
non-transc.
other
p
a a b c d
mean z-scores
0h
1h
3h
6h
12h
time point

Supplementary Fig. 2. Widespread production of new mRNAs during seed imbibition. **A)** Seed scarification enhances the 4-SU labelling of heat-inducible genes during imbibition. Seeds were initially imbibed for 5 h at 22°C and then transferred to 38°C for 1h. The plot shows the log2 fold change between scarified and intact seeds in the level of enriched labelled mRNAs. Three biological replicates are shown with lines depicting means; Source Data file contains exact p-values. **B)** Sanger sequencing results for *HSP17.6II* mRNA after converting 4-SU to cytidine. From the bottom: unlabelled sample, labelled sample, biotin-enriched labelled sample. **C)** Plot comparing input and enriched mRNAs after 4-SU labelling, with identified new mRNAs highlighted in blue and their number indicated on plots (DESeq2; n=4; ERCC-based normalisation; padj < 0.05; $log_2FC > 2$; red line – the trend for ERCC). New mRNAs were identified for 3 h of imbibition. The yellow dot denotes a positive control spike-in with 100% 4-SU labelling. **D)** Ratio of T to C conversion in control (non-chemically converted), total and streptavidin-enriched mRNAs (DESeq2; n=4; ERCC-based normalisation; padj < 0.05; $log_2FC > 2$). Boxplots show the ratio for new mRNAs that were called new or not in this experiment. Spike-ins are presented as separate dots: ERCC – red; positive (100% labelled with 4-SU) – yellow. Boxplot central line represents median; whiskers represent 1.5 times the interquartile range; outliers are indicated with dots. E**)** A comparison of U-to-C conversion levels in total mRNA samples with biotin enrichment after 3 h of labelling with 4-SU (enrichment is based on ERCC estimation of unlabelled molecule carry-over; point density is illustrated using the Viridis scale). **F)** Plots comparing input and enriched mRNAs after 4-SU labelling, with identified new mRNAs highlighted in blue and their number indicated on plots (DESeq2; n=4; ERCC-based normalisation; padj < 0.05; $log_2FC > 2$; red line – the trend for ERCC). New mRNAs were identified for 1 h and 3 h of imbibition. **G)** Venn diagrams illustrating the overlap in identified new mRNAs between this experiment and the experiment in Fig. 2b,c. **H)** Comparison of new mRNA production and the effect of α-amanitin treatment (point density is illustrated with the Viridis scale; the line represents a trend). **I)** Heat map plot showing the increase of new mRNA levels enriched on beads during the time course of 4-SU labelling. Z-scores were calculated using ERCC-normalised CPMs. **J)** Plots comparing input and enriched mRNAs after 30 min of 4-SU labelling of squeezed seeds. Identified new mRNAs are highlighted in blue and their number are indicated on plots (DESeq2; n=4; ERCC-based normalisation; padj < 0.05; $log_2FC > 2$; red line – the trend for ERCC). Labelling was preceded by 2.5 h and 5.5 h of imbibition. **K)** Venn diagrams illustrating the overlap in identified new mRNAs between 30 min and continuous labelling in Fig. 2c,d. **L)** New mRNA labelling, enrichment and detection using 5-EU-RT-qPCR protocol (6 h imbibition). Enrichment was calculated as a percentage of the input used. Four biological replicates are shown with lines depicting means. Above the plot, log2FC values from 4-SU 3'RNA-seq detection (Fig. 2d) are shown; colours indicate whether the mRNA belongs to the clusters identified in Fig. 1. **M)** mRNAs from 3 h imbibed seeds were binned by levels in input and enriched new mRNA samples (mean of four replicates), with each bin's content shown as a percentage of all mRNAs. **N)** Plot comparing input and enriched mRNAs after 3 h 4-SU labelling for genes identified as not being transcribed during imbibition. **O)** mRNAs not transcribed during imbibition (Fig. 2c,d; DESeq2; $log_2FC < 1$ at 3 h and 6 h) show a high level of accumulation in total mRNA samples (two-sided t-test p-value assesses the difference to all other detected mRNAs;**** < 0.0001). **P)** mRNAs not transcribed during imbibition are mostly downregulated (data from the time course Fig. 1b; mRNA levels are in z-score units; letters represent statistical differences (< 0.05) using one-way ANOVA with Tukey's post hoc test; Source Data file contains exact p-values). (O) and (P) Boxplot central line represents median; whiskers represent 1.5 times the interquartile range; outliers are indicated with dots. A and L - Source data are provided as a Source Data file.

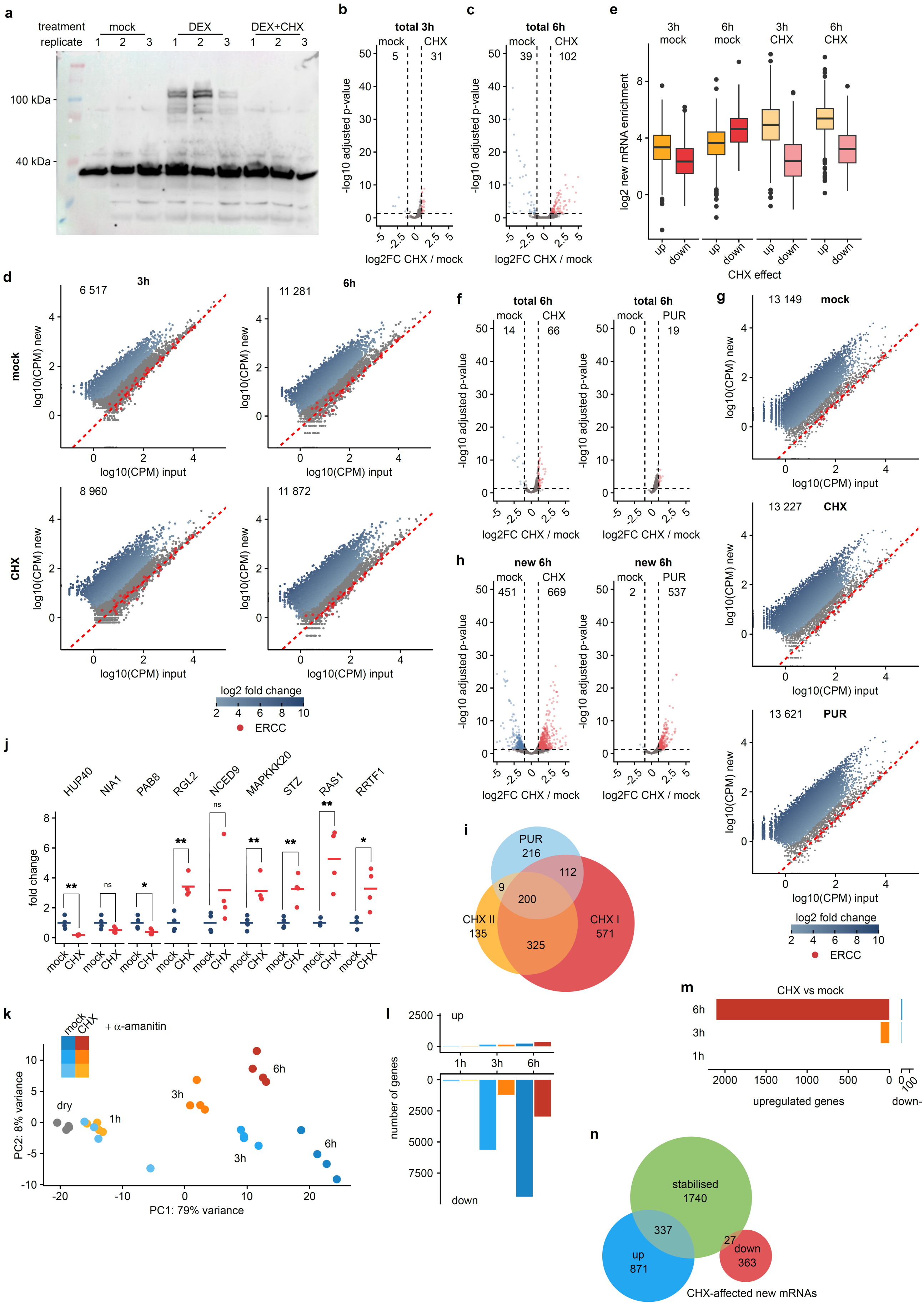

a
treatment mock DEX DEX+CHX
replicate 1 2 3 1 2 3 1 2 3
100 kDa
40 kDa
b
total 3h
mock 5
CHX 31
-log10 adjusted p-value
log2FC CHX / mock
c
total 6h
mock 39
CHX 102
-log10 adjusted p-value
log2FC CHX / mock
e
3h mock
6h mock
3h CHX
6h CHX
log2 new mRNA enrichment
CHX effect
up
down
d
3h
6h
mock
CHX
6 517
11 281
8 960
11 872
log10(CPM) new
log10(CPM) input
log2 fold change
ERCC
f
total 6h
mock 14
CHX 66
mock 0
PUR 19
-log10 adjusted p-value
log2FC CHX / mock
g
13 149 mock
13 227 CHX
13 621 PUR
log10(CPM) new
log10(CPM) input
log2 fold change
ERCC
h
new 6h
mock 451
CHX 669
mock 2
PUR 537
-log10 adjusted p-value
log2FC CHX / mock
j
HUP40
NIA1
PAB8
RGL2
NCED9
MAPKKK20
STZ
RAS1
RRTF1
fold change
mock
CHX
i
PUR 216
112
9
200
CHX II 135
325
CHX I 571
k
mock CHX + α-amanitin
dry
1h
3h
6h
PC2: 8% variance
PC1: 79% variance
l
up
down
number of genes
1h 3h 6h
m
CHX vs mock
6h
3h
1h
upregulated genes
down-
n
stabilised 1740
337
27
up 871
down 363
CHX-affected new mRNAs

Supplementary Fig. 3. Effects of cycloheximide on new mRNA levels during imbibition. **A)** Western blot (uncropped) for 3xmCherry reporter under the control of a DEX-inducible promoter. Seeds were imbibed in mock, DEX or DEX + CHX for 6 h. The expected band size is 100.5 kDa. Unspecific bands were used as a loading control; n=3 biological replicates. **B-C)** Volcano plot shows CHX effects on differential gene expression; the number of DEGs is indicated (DESeq2; n=3; ERCC-based normalisation; padj < 0.05; -log10(padj); |log2FC| > 1, capped at 5) during imbibition for 3 h (A) and 6 h (B). **D)** Plots comparing input and enriched mRNAs after 4-SU labelling in the presence of CHX or mock, with new mRNAs highlighted in blue and their number indicated (DESeq2; n=3; ERCC-based normalisation; padj < 0.05; $\log_2$FC > 2; red line – the trend of ERCC enrichment) for 3 h and 6 h of imbibition. **E)** New mRNA production in mock and CHX-treated seeds (3 h and 6 h) for genes exhibiting altered new mRNA levels following CHX treatment (boxplot central line represents median; whiskers represent a 1.5 interquartile range, and outliers are indicated with dots). **F)** Volcano plot shows CHX (second experiment) and puromycin effects on differential gene expression during 6 h of imbibition; the number of DEGs is indicated (DESeq2; n=3; ERCC-based normalisation; padj < 0.05; -log10(padj); |log2FC| > 1). **G)** Plots comparing input and enriched mRNAs after 4-SU labelling in the presence of CHX (second experiment), puromycin (PUR) or mock for 6 h of imbibition. New mRNAs are highlighted in blue, and their number is indicated (DESeq2; n>=3; ERCC-based normalisation; padj < 0.05; $\log_2$FC > 2; red line – the trend of ERCC enrichment). **H)** Volcano plot shows CHX and puromycin effects on new mRNA accumulation during 6 h imbibition. The number of DEGs is indicated (DESeq2; n=3; ERCC-based normalisation; padj < 0.05; -log10(padj); |log2FC| > 1, capped at 5). **I)** Venn diagram illustrating the overlap in upregulated new mRNAs between both CHX and puromycin treatments. **J)** RT-qPCR analysis of 4-SU-labelled new mRNA enrichment in mock and CHX-treated seeds. Enrichment values are presented relative to mock-imbibed seeds, with *ACT7* mRNA as the reference. Lines indicate the mean. Two-sided t-test p-value: ns – non-significant, * < 0.05, ** < 0.01; Source Data file contains exact p-values. **K)** PCA plot summarising 3'RNA-seq results (n=4) from seeds imbibed in the presence of α-amanitin or α-amanitin and CHX. Data were normalised using ERCC spike-ins. **L)** The number of DEGs (DESeq2; |$\log_2$FC| > 1, padj < 0.05) when comparing α-amanitin or α-amanitin and CHX-treated samples to dry seeds. The top plot displays genes with upregulated expression (up), while the bottom plot shows genes with downregulated (dw) expression. **M)** Number of DEGs (DESeq2; |$\log_2$FC| > 1, padj < 0.05) when comparing α-amanitin with α-amanitin and CHX-treated samples. **N)** Venn diagram illustrating the overlap in total mRNAs upregulated by the CHX treatment in the presence of α-amanitin, with new mRNAs showing differential accumulation due to CHX-treatment (Fig. 3d). J - Source data are provided as a Source Data file.

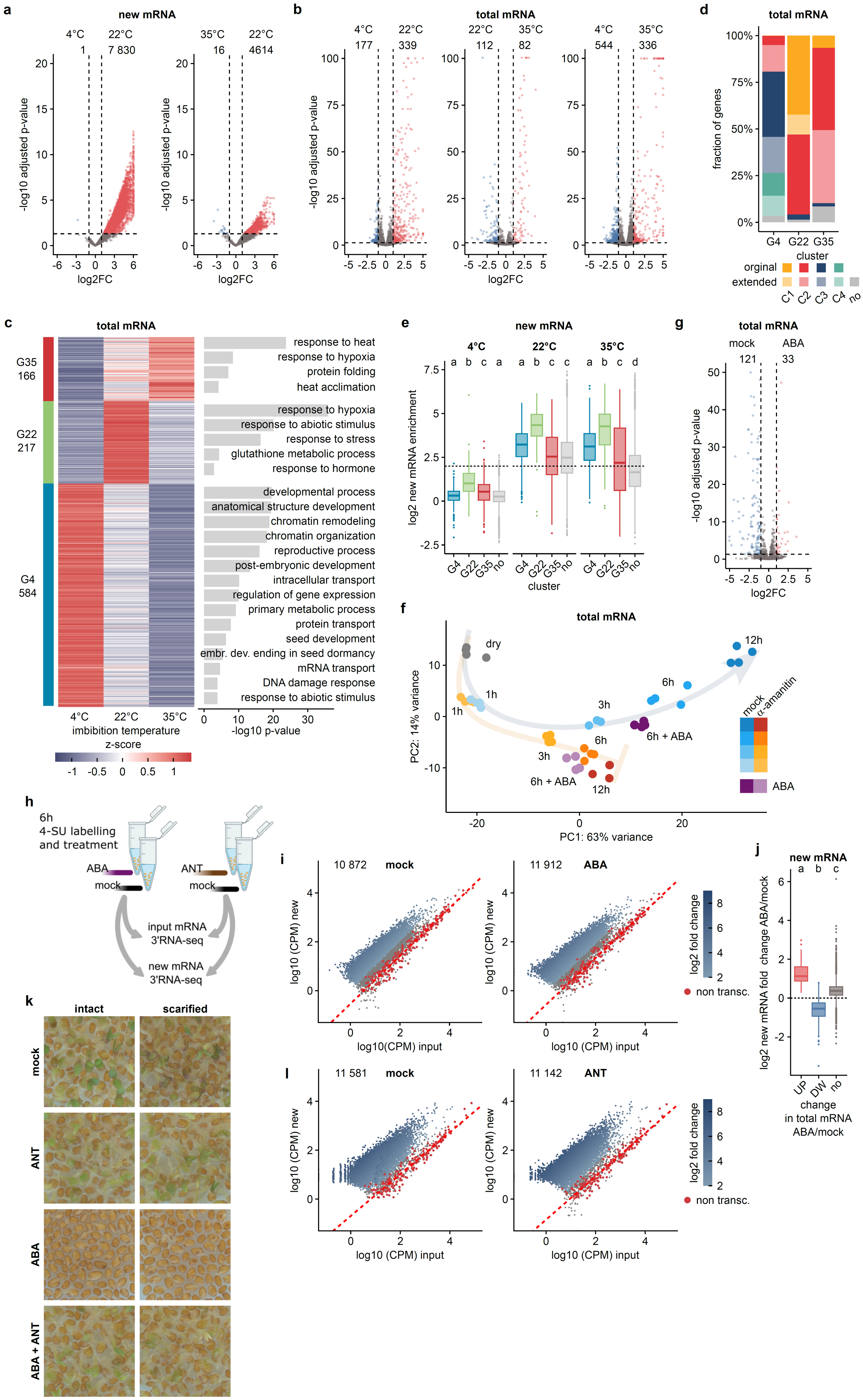
a
new mRNA
4°C
22°C
1
7 830
35°C
22°C
16
4614
-log10 adjusted p-value
log2FC
b
total mRNA
4°C
22°C
177
339
22°C
35°C
112
82
4°C
35°C
544
336
-log10 adjusted p-value
log2FC
d
total mRNA
fraction of genes
G4 G22 G35
cluster
orginal
extended
C1 C2 C3 C4 no
c
total mRNA
G35
166
G22
217
G4
584
response to heat
response to hypoxia
protein folding
heat acclimation
response to hypoxia
response to abiotic stimulus
response to stress
glutathione metabolic process
response to hormone
developmental process
anatomical structure development
chromatin remodeling
chromatin organization
reproductive process
post-embryonic development
intracellular transport
regulation of gene expression
primary metabolic process
protein transport
seed development
embr. dev. ending in seed dormancy
mRNA transport
DNA damage response
response to abiotic stimulus
4°C 22°C 35°C
imbibition temperature
z-score
-log10 p-value
e
new mRNA
4°C
22°C
35°C
a b c a
a b c c
a b c d
log2 new mRNA enrichment
G4 G22 G35 no
cluster
g
total mRNA
mock
ABA
121
33
-log10 adjusted p-value
log2FC
f
total mRNA
dry
1h
3h
6h
12h
6h + ABA
mock
α-amanitin
ABA
PC2: 14% variance
PC1: 63% variance
h
6h
4-SU labelling
and treatment
ABA
mock
ANT
mock
input mRNA
3'RNA-seq
new mRNA
3'RNA-seq
i
10 872
mock
11 912
ABA
log10 (CPM) new
log10(CPM) input
log10 (CPM) input
log2 fold change
non transc.
j
new mRNA
a b c
log2 new mRNA fold change ABA/mock
UP DW no
change
in total mRNA
ABA/mock
k
intact
scarified
mock
ANT
ABA
ABA + ANT
l
11 581
mock
11 142
ANT
log10 (CPM) new
log10 (CPM) input
log2 fold change
non transc.

Supplementary Fig. 4. The early new mRNA production phase during seed imbibition is robust. **A)** Volcano plots show the effects of varying temperatures during 3 h of imbibition on new mRNA production; the number of DEGs is indicated (DESeq2; n=4; ERCC normalisation; padj < 0.05; $|log_2FC| > 1$, capped at 6). **B)** Volcano plots show the effects of different temperatures during 3 h of imbibition on total mRNA levels; the number of DEGs is indicated on the plots (DESeq2; n=4; padj < 0.05; $-log_{10}(padj)$ capped at 100; $|log_2FC| > 1$, capped at 5). **C)** Grouping of genes exhibiting differential expression due to varying temperature during imbibition (WGCNA with z-score normalised data). GO-term enrichment results are shown for representative biological process terms. **D)** Assignment of genes belonging to G4/22/35 clusters to clusters defined during the imbibition time course (Fig. 1e). The extension of clusters was based on the similarity of the profiles. **E)** mRNA production across imbibition temperatures for genes included in expression clusters affected by the imbibition temperature (C). **F)** PCA plot summarising 3'RNA-seq results shown in Fig. 1b from dry and imbibed seeds (n=3-4), with arrows manually added to show the changed transcriptomic trajectories. 6 h ABA-treated samples are included. **G)** A volcano plot illustrating changes in gene expression due to 6 h ABA treatment; the number of DEGs is indicated (DESeq2; selected stable genes normalisation; n=4; padj < 0.05; $-log_{10}(padj)$ capped at 50; $|log_2FC| > 1$, capped at 5). **H)** Diagram demonstrating an imbibition mRNA labelling experiment upon treatment with ABA and antabactin. Created in BioRender. Halale manjunath, V. (2026) https://BioRender.com/1vzolgr. **I)** Plots comparing input and enriched mRNAs after 6 h of 4-SU labelling for mocks and ABA. **J)** New mRNA production changes upon treatment with ABA for genes with differences in total mRNA levels (G). E and J) Central line represents median; whiskers indicate a 1.5 interquartile range; outliers are marked with dots; letters denote statistical differences (< 0.05) using one-way ANOVA with Tukey's post hoc test; Source Data file contains exact p-values. **K)** Germination phenotypes at 2 days post-imbibition for seeds imbibed in ABA, ANT, ABA + ANT, and mock solutions. **L)** Plots comparing input and enriched mRNAs after 6 h of 4-SU labelling for mock and ANT. I and L) New mRNAs are highlighted in blue and their number indicated in the plots (DESeq2; n=4; non-transcribed genes normalisation; padj < 0.05; $log_2FC > 2$; red line – the trend of unlabelled mRNA enrichment).

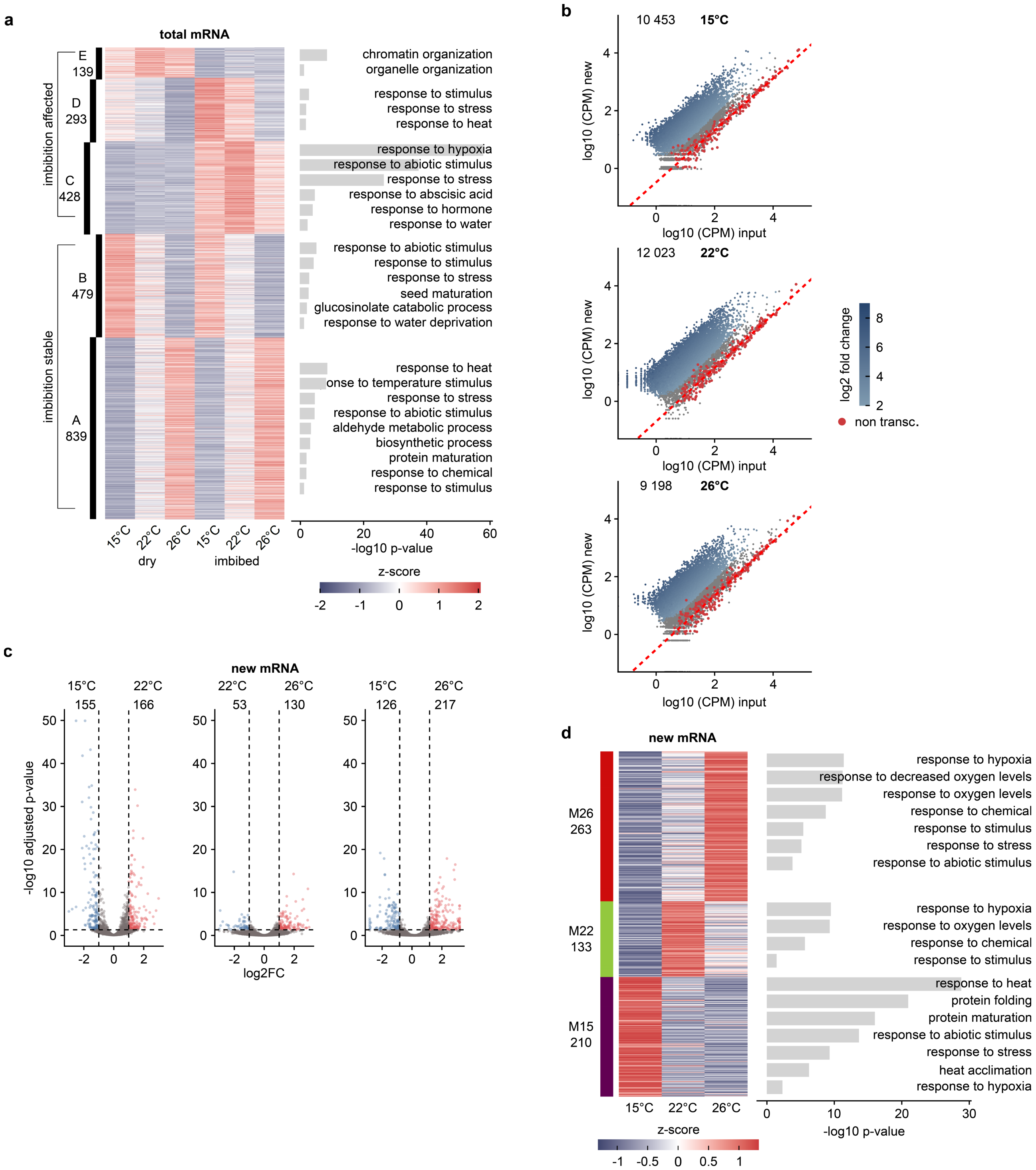

Supplementary Fig. 5. Seed maturation temperature has a minor effect on mRNA production during imbibition. **A)** Grouping of genes exhibiting differential mRNA expression (in dry and 3 h imbibed seeds) due to varying temperature during maturation (DESeq2; n=3; padj < 0.05; |log2FC| > 1, WGCNA with z-score normalised data). GO-term enrichment results are shown for representative biological process terms. This analysis identified five major gene clusters, which can be categorised into those responding (affected) and those not responding to imbibition (stable). **B)** Plots comparing input and enriched mRNAs after 3 h imbibition with 4-SU labelling of seeds matured at different temperatures. New mRNAs are highlighted in blue, and their number is indicated in the plots (DESeq2; n=3; non-transcribed genes normalisation; padj < 0.05; $\log_2$FC > 2; red line – the trend of unlabelled mRNA enrichment). **C)** Volcano plots show the effects of varying seed maturation temperatures on new mRNA production during 3 h of imbibition (the number of affected genes is indicated in the plots; DESeq2; n=3; padj < 0.05; -$\log_{10}$(padj) capped at 50; |$\log_2$FC| > 1, capped at 3; mRNAs not identified as newly produced (C) are excluded). **D)** Grouping of genes

exhibiting differential mRNA production in seeds matured at different temperatures (Supplementary Fig. 5c; WGCNA with z-score normalised data). GO-term enrichment results are shown for representative biological process terms.

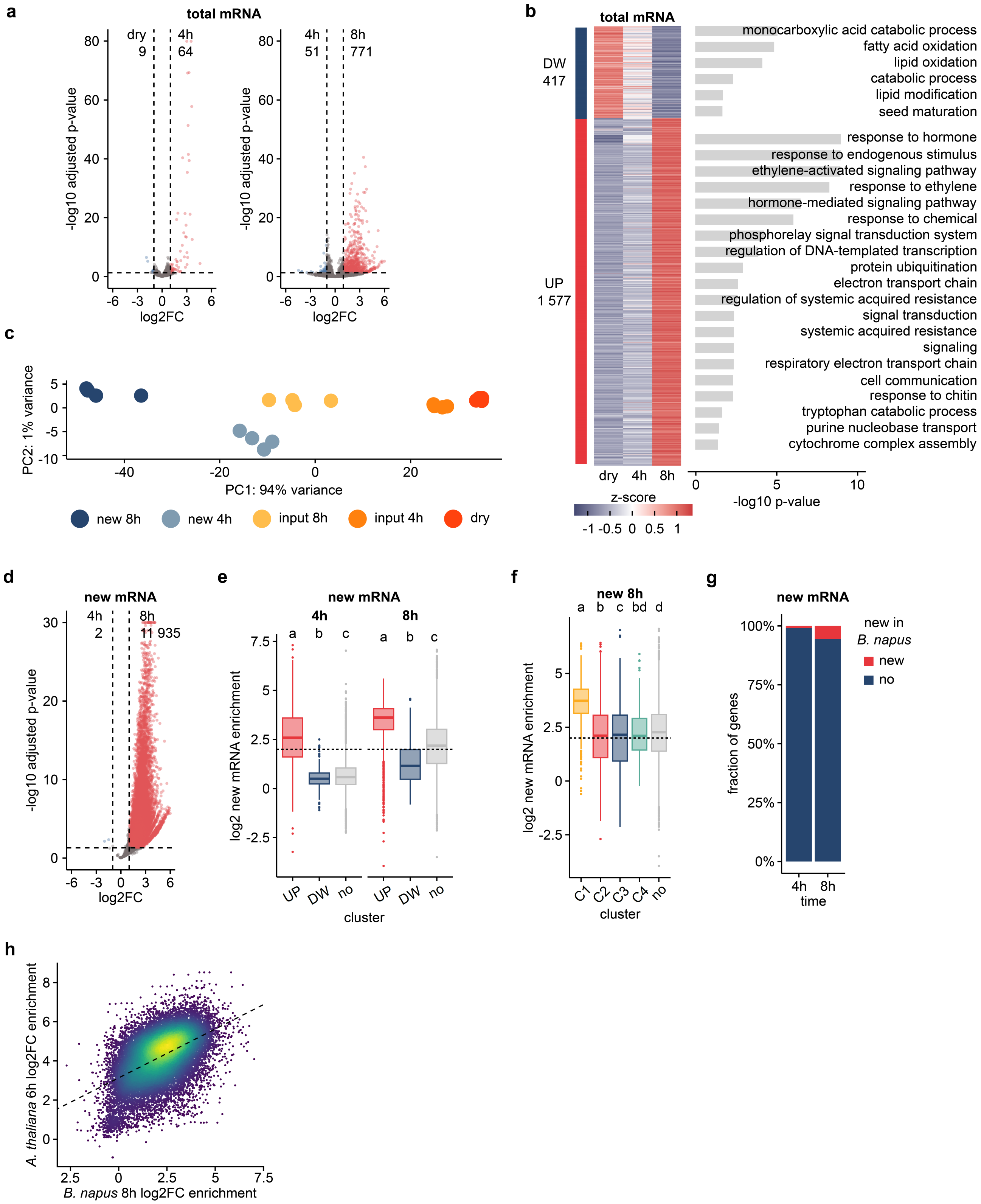

Supplementary Fig. 6. The pattern of new transcription is conserved during *B. napus* imbibition. **A)** Volcano plots showing changes in gene expression during *B. napus* seed imbibition (the number of affected genes is indicated; DESeq2; n=4; padj < 0.05; -$\log_{10}$(padj) capped at 80; |$\log_2$FC| > 1, capped at 6). **B)** Clustering of genes exhibiting differential expression during *B. napus* imbibition (Fig. 5b and Supplementary Fig. 6a; WGCNA with z-score normalised data). GO-term enrichment results are shown for representative biological process terms. **C)** PCA plot summarising *B. napus* 3'RNA-seq results from total (input) and new mRNA-enriched samples (n=4). **D)** A volcano plot shows the difference in new mRNA production in *B. napus* seeds between 8 h and 4 h of imbibition; the number of DEGs is indicated in the plot (DESeq2; n=4; downregulated genes normalisation – see text; padj < 0.05; -$\log_{10}$(padj) capped at 30; |$\log_2$FC| > 1, capped

at 6; mRNAs not identified as newly produced (Fig. 5c) are excluded). **E)** New mRNA production during *B. napus* seeds imbibition for genes included in expression clusters (Supplementary Fig. 6b). **F)** New mRNA production during 8 h of imbibition for homologues of genes included in expression clusters affected during Arabidopsis imbibition (Fig. 1E). In (E-F), central line represents median; whiskers denote a 1.5 interquartile range, outliers are marked with dots, and letters represent statistical differences (< 0.05) using one-way ANOVA with Tukey's post hoc test; Source Data file contains exact p-values. **G)** Overlap of Arabidopsis imbibition non-transcribed genes with their homologous in *B. napus*. **H)** Comparison of new mRNA production (beads enrichment) in *B. napus* (8h) and Arabidopsis (6h). Point density is illustrated with the Viridis scale; the line represents a trend.

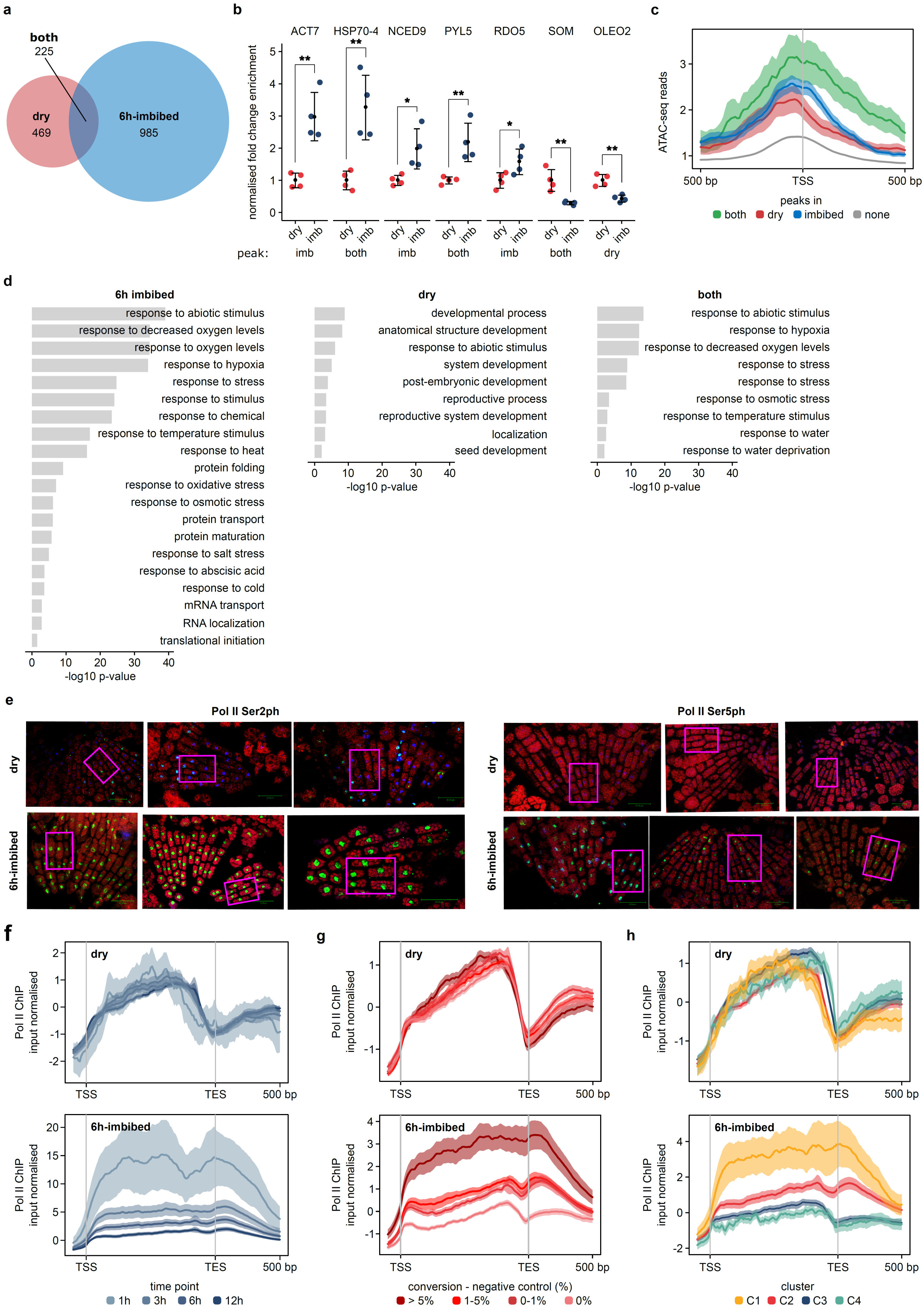
a
both
225
dry
469
6h-imbibed
985
b
ACT7
HSP70-4
NCED9
PYL5
RDO5
SOM
OLEO2
normalised fold change enrichment
**
**
*
**
*
**
**
dry
imb
peak:
imb
both
imb
both
imb
both
dry
c
ATAC-seq reads
500 bp
TSS
500 bp
peaks in
both
dry
imbibed
none
d
6h imbibed
response to abiotic stimulus
response to decreased oxygen levels
response to oxygen levels
response to hypoxia
response to stress
response to stimulus
response to chemical
response to temperature stimulus
response to heat
protein folding
response to oxidative stress
response to osmotic stress
protein transport
protein maturation
response to salt stress
response to abscisic acid
response to cold
mRNA transport
RNA localization
translational initiation
-log10 p-value
dry
developmental process
anatomical structure development
response to abiotic stimulus
system development
post-embryonic development
reproductive process
reproductive system development
localization
seed development
-log10 p-value
both
response to abiotic stimulus
response to hypoxia
response to decreased oxygen levels
response to stress
response to stress
response to osmotic stress
response to temperature stimulus
response to water
response to water deprivation
-log10 p-value
e
Pol II Ser2ph
Pol II Ser5ph
dry
6h-imbibed
f
dry
6h-imbibed
Pol II ChIP
input normalised
TSS
TES
500 bp
time point
1h
3h
6h
12h
g
dry
6h-imbibed
Pol II ChIP
input normalised
TSS
TES
500 bp
conversion - negative control (%)
> 5%
1-5%
0-1%
0%
h
dry
6h-imbibed
Pol II ChIP
input normalised
TSS
TES
500 bp
cluster
C1
C2
C3
C4

Supplementary Fig. 7. Pol II binding in dry seeds reflects the gene expression patterns associated with seed maturation. **A)** A Venn diagram for genes with Pol II peaks in dry and 6 h imbibed seeds. **B)** ChIP-qPCR results showing Pol II binding at selected genes. Chromatin was isolated from dry and 6 h imbibed seeds (n=4). The signal was normalised to Pol II binding at the *TUB2* gene and then to the average dry seed signal. Dots represent biological replicates; black dots indicate mean values, and error bars correspond to standard deviations. The results of the paired two-sided t-test are shown for each gene (p-value < 0.05 *; < 0.01 **; Source Data file contains exact p-values). The presence of peaks in the ChIP-seq data is indicated below the plot. **C)** Metaprofiles of ATAC-seq in dry seeds (Tremblay *et al.*, 2024) (GSE250331) for the promoter regions of genes with Pol II peaks (A). The plot shows data binned into 10 bp intervals with solid lines representing means and shaded areas indicating 95% confidence intervals. **D)** GO-term enrichment results for genes with Pol II peaks are shown for representative biological process terms. **E)** Immunolocalisation results for phosphorylated Pol II (Ser2ph and Ser5ph) in embryo root tip cells (scale bar is 20 µm; blue – DAPI staining; red – cell autofluorescence; green – Alexa Fluor Plus 488; rectangles indicate fragments of the images cropped to be presented in Fig. 6b). **F)** Metaprofiles of Pol II binding along genes exhibiting upregulated expression during imbibition. **G)** Metaprofiles of Pol II binding along genes that differ in the percentage of reads with converted U to C (conversions in total mRNAs – negative control; Fig. 2f). **H)** Metaprofiles of Pol II binding along genes included in expression clusters affected during imbibition (Fig. 1E). (F-H) ChIP-seq data (n=3) were normalised to input, averaged and binned into 10 bp intervals (solid lines show means, and shaded areas indicate 95% confidence intervals; TSS refers to the transcription start site, and TES refers to the transcription end site). B - Source data are provided as a Source Data file.

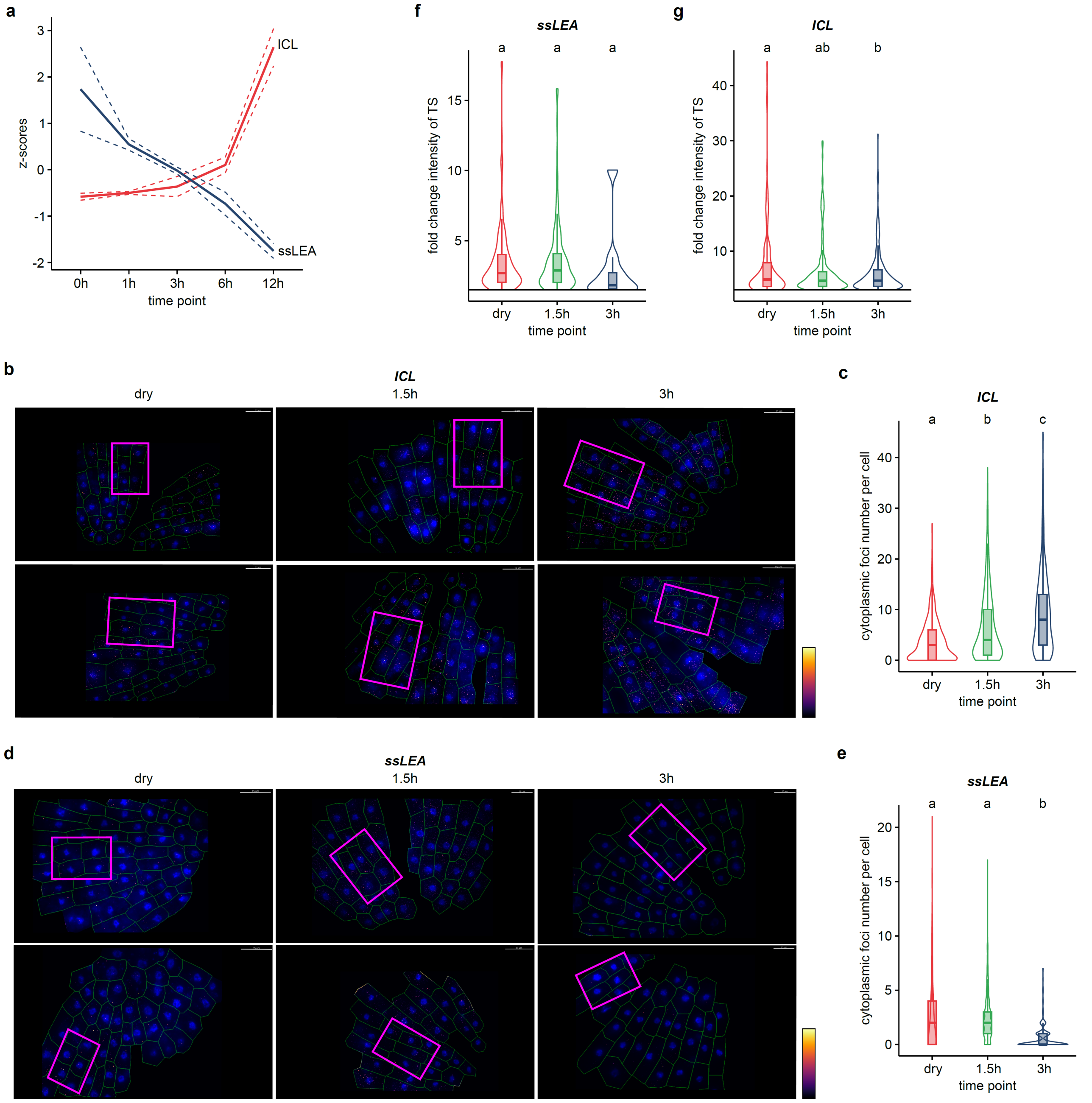

Supplementary Fig. 8. smFISH analysis confirms limited production of mRNAs for a gene with a Pol II peak specific to dry seeds. **A)** The levels (in z-score units) of *ICL* and *ssLEA* mRNAs across the time course of imbibition (Fig. 1B; solid lines represent the means; dashed lines indicate the standard deviations of replicates; n>=3). **B)** *z*-stack max-projection images of *ICL* smFISH in the embryo root tip (scale bar is 15 µm; blue -DAPI staining; arbitrary smFISH probes linear intensity scale is shown; rectangles indicate fragments of the images cropped to be presented in Fig. 6E). **C)** The average number of cytoplasmic foci for *ICL* mRNA in each analysed cell. **D)** *z*-stack max-projection images of *ssLEA* smFISH in the embryo root tip (scale bar is 15 µm; blue -DAPI staining; arbitrary smFISH probes linear intensity scale is shown; rectangles indicate fragments of the images cropped to be presented in Fig. 6G). **E)** The average number of cytoplasmic foci for *ssLEA* mRNA in each analysed cell. **F)** Mean signal intensity per TS foci for *ssLEA* and **G)** *ICL* probes. In (C, D, F-G) the central line represents the median; whiskers denote a 1.5 interquartile range, and letters represent statistical differences (< 0.05) using one-way ANOVA with Tukey's post hoc test; Source Data file contains exact p-values.

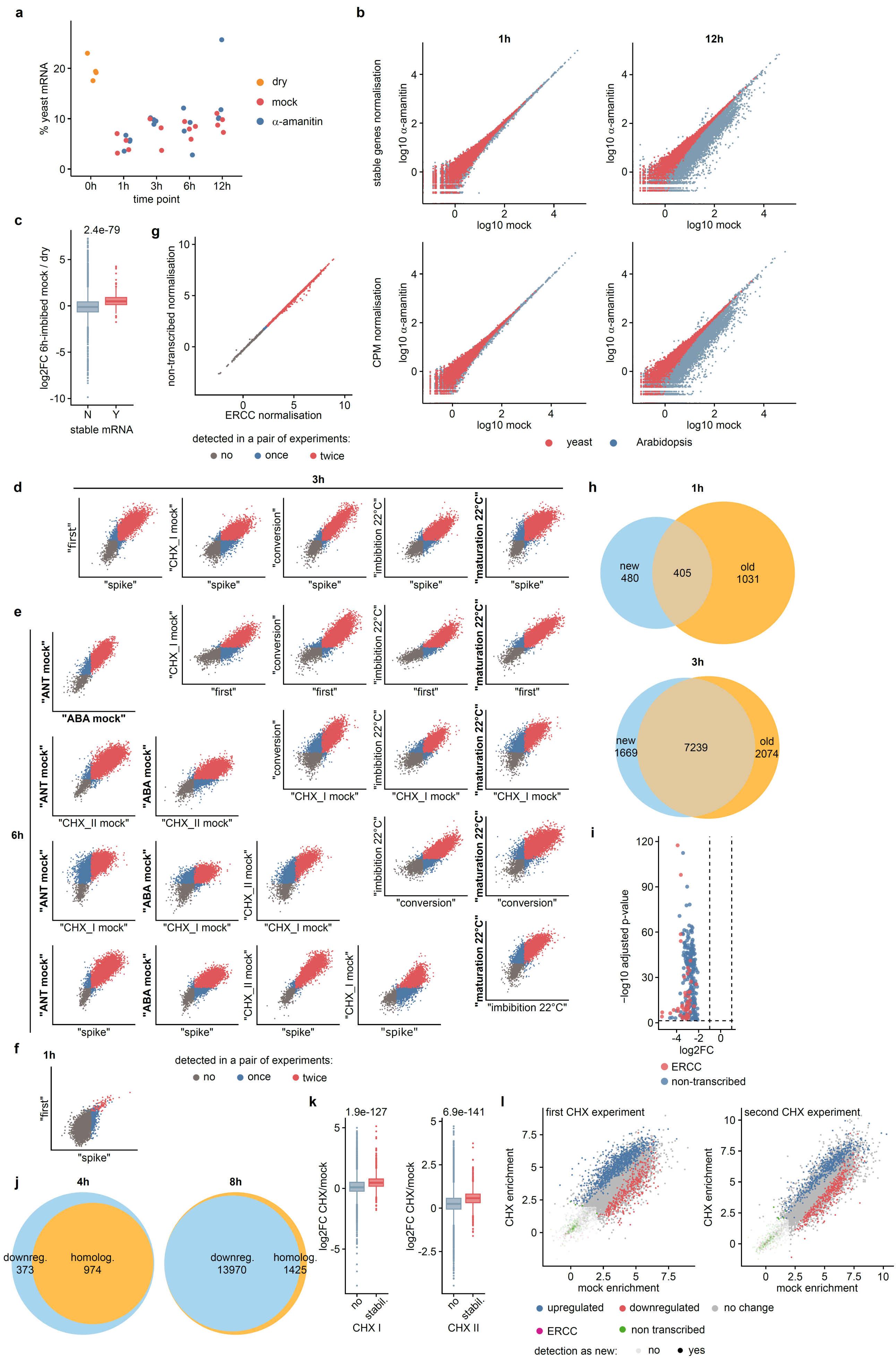

a
% yeast mRNA
time point
0h
1h
3h
6h
12h
dry
mock
α-amanitin
b
1h
12h
stable genes normalisation
CPM normalisation
log10 α-amanitin
log10 mock
yeast
Arabidopsis
c
2.4e-79
log2FC 6h-imbibed mock / dry
stable mRNA
N
Y
g
non-transcribed normalisation
ERCC normalisation
detected in a pair of experiments:
no
once
twice
d
3h
"first"
"CHX_I mock"
"conversion"
"imbibition 22°C"
"maturation 22°C"
"spike"
e
6h
"ANT mock"
"ABA mock"
"CHX_II mock"
"CHX_I mock"
"first"
"conversion"
"imbibition 22°C"
"spike"
f
1h
"first"
"spike"
detected in a pair of experiments:
no
once
twice
h
1h
new 480
405
old 1031
3h
new 1669
7239
old 2074
i
−log10 adjusted p-value
log2FC
ERCC
non-transcribed
j
4h
downreg. 373
homolog. 974
8h
downreg. 13970
homolog. 1425
k
1.9e-127
6.9e-141
log2FC CHX/mock
no
stabil.
CHX I
CHX II
l
first CHX experiment
second CHX experiment
CHX enrichment
mock enrichment
upregulated
downregulated
no change
ERCC
non transcribed
detection as new:
no
yes

Supplementary Fig. 9. Additional bioinformatic analyses. **A)** Content of yeast mRNAs in 3'RNA-seq libraries after mapping to *Arabidopsis thaliana* and *Saccharomyces cerevisiae* genomes. Most likely due to the high content of seed storage materials, the RNA concentration measurements proved unreliable, leading to an uneven spike-in addition. **B)** Plots comparing Arabidopsis gene expression in mock and α-amanitin-treated samples for 1 h and 12 h, with yeast mRNAs used as spike-ins. The bottom row displays results for CPM normalised data, while the top row shows gene expression normalised using a set of stable mRNAs. Both approaches produced very similar outcomes, validating the use of stable genes for normalisation. **C)** mRNAs classified as stable (DESeq2; padj < 0.05, |log2FC| < 1, altHypothesis = "lessAbs") when comparing dry and 6-hour imbibed seeds with α-amanitin display an increase during imbibition without α-amanitin (Fig. 1). The central line represents the median; boxplot whiskers indicate a 1.5 interquartile range, and outliers are marked with dots. A p-value of the two-sided t-test is presented above the plot. **D-F)** Comparison of 4-SU labelling experiments. Red dots indicate mRNAs identified in both experiments, while blue dots indicate those found in only one. The name of the experiment in bold indicates no newly transcribed mRNA normalisation instead of ERCC normalisation. D) 3h-labelling experiments: "spike" - Fig. 2c, "first" - Supplementary Fig. 2d, "CHX_I_mock" - Supplementary Fig. 3d, "conversion" - Supplementary Fig. 2f, "imbibition 22°C" - Fig. 4b, and "maturation 22°C" - Supplementary Fig. 5b. E) 6h-labelling experiments: "spike" - Fig. 2d, "CHX_I_mock" - Supplementary Fig. 3d, "CHX_II_mock" - Supplementary Fig. 3g, "ABA mock" - Supplementary Fig. 4i, and "ANT mock" - Supplementary Fig. 4l. F) 1h-labelling experiments: "spike" - Fig. 2b and "first" - Supplementary Fig. 2d. **G)** Comparison of ERCC-based and non-newly transcribed mRNA normalisation for identifying new mRNAs (Fig. 2c). Red dots denote mRNAs identified by both methods, while blue dots indicate those identified by only one method. **H)** Venn diagrams illustrating the overlap in identified new mRNAs between two experiments, shown in Fig. 2b-d and Supplementary Fig. 2f, when using non-transcribed genes normalisation. Compare with the overlap when ERCC normalisation is used - Supplementary Fig. 2g. **I)** A volcano plot illustrates the change in the levels of ERCC and non-newly transcribed genes when comparing new mRNA-enriched samples with input samples without subsetting genes for normalisation (Fig. 2c; DESeq2; n=4; padj < 0.05; |log2FC| > 1). **J)** Venn diagrams illustrating the overlap in identified new mRNAs in *B. napus*, when using "downregulated transcripts" normalisation as in Fig. 5 and identified "homologues of Arabidopsis non-transcribed genes" normalisation. **K)** mRNAs categorised as stabilised (DESeq2; padj < 0.05, log2FC > 1) when comparing CHX-treated and CHX-non-treated 6-hour imbibed seeds with α-amanitin (Supplementary Fig. 3K) show an increase during imbibition without α-amanitin (Supplementary Fig. 3c – CHX I and Supplementary Fig. 3f - CHX II). The boxplot central line represents the median; whiskers represent 1.5 times the interquartile range, with outliers marked as dots. The p-value from the two-sided t-test is displayed above the plots. **L)** Plots comparing the enrichment of beads-bound mRNAs in mock and CHX-treated seeds (two experiments). mRNAs with differential enrichment are shown alongside ERCC spike-ins and mRNAs identified as not undergoing transcription.

Supplementary Table 1. Primer list.

name	gene	experiment	left primer	right primer
HSP17.6II	AT5G12020	PCR for Sanger	AGACCACAACAACGAGAAGAC	CCRTRTCCCTCRCGCRTTCCG
UBC21	AT5G25760	RT-qPCR	CTGCGACTCAGGGAATCTTCTAA	TTGTGCCATTGAATTGAACCC
HSP17.6II	AT5G12020	RT-qPCR	GCTAAGGCAATGGCTGCTACA	CGTTCTCGACCTGAACCTTGA
HSP70-4	AT3G12580	RT-/ChIP-qPCR	GTGATCCCTCTGTTCAAGCGG	CTCACCTGGACCGGAAACAAC
HSP89.1	AT3G07770	RT-qPCR	TGCTCAAAGTGGAACCGCCAAG	TGTCACCACCTGCATCCTTGC
SRO5	AT5G62520	RT-qPCR	CAGAGTTTGATTCTGGTGTGGATG	TCTCTTTGGACTTCGAGTTAGG
TUB2	AT5G62690	ChIP-qPCR	ATCCGTGAAGAGTACCCAGAT	AAGAACCATGCACTCATCAGC
ACT7	AT5G09810	RT-/ChIP-qPCR	CCACAGTCCGTTTGTCTGCTCT	CGGCTCGGCTTTTGTGGTTCG
NCED9	AT1G78390	RT-/ChIP-qPCR	ACCTGTCGTGCATAACCTTCC	GGATTTGCTCCGTTTCTGACG
PYL5	AT5G05440	RT-/ChIP-qPCR	TCATCAGACAGTGCCGTATCG	GAGTCCAGAGACCACCATGAC
RDO5	AT4G11040	ChIP-qPCR	TAGTCGATGCACTAATGGCCG	CCCATGACTGAGACTACACCG
SOM	AT1G03790	RT-/ChIP-qPCR	GCAACAACAACCAAGTAGCCC	AGGTAACTTGGAGTGGAAGGC
OLEO2	AT5G40420	ChIP-qPCR	CTTCAGCCCGGTTATAGTCCC	CCGGTTAGACCGAACATCCC
RAS1	AT1G09950	RT-qPCR	GATCAGCTACGGTGTTCGACG	GGAGGTGAAGCTGTCCGATTG
P85	AT4G35985	RT-qPCR	CGATCACAAGTATGGAGCAAAGAC	AAACAGTCCACGCCGTTCC
RRTF1	AT4G34410	RT-qPCR	ATGAGCTCTTTCTTGTTGGCG	TGTTCACTTTAAACAAGCCACAG
MAPKKK20	AT3G50310	RT-qPCR	TGAAGAAACTTGGCGGTGAGG	GTGACGTAACCCTCGAAGCAC
RGL2	AT3G03450	RT-qPCR	CGAGGAAGCTAGCAGCAAGAG	CTGAGAGTCAACGAGCACCAC
HUP40	AT4G24110	RT-qPCR	CGTGTAGCGGCGAATCTGAC	AGACTCCATCTCAGCCGTCC
ICL	AT3G21720	RT-qPCR	GAAAGGTCCTCGTGGCAGTC	CATCACGTCGAACTGGAGCC
PAB8	AT1G49760	RT-qPCR	TGGTGGTGTGCATCATCGTG	TCTGGAGCTGCATTCGCAAG
LSM3B	AT1G76860	RT-qPCR	ATTGTTCGGACTACAAAGCGG	TCCTCAGCGGTGGAGACAC
BRM	AT2G46020	RT-qPCR	TCTCTGGGCAGTCAGGATCAG	TTCTGGGACTGGAAGTCGAGG
DREB2C	AT2G40340	RT-qPCR	GCCAGACTCAATCTTCCCGAG	AATGCAGTAACCGAGCCTGAC
DREB2B	AT3G11020	RT-qPCR	ATGAACAAACCGGAACCGAGC	ATCAGCCACCGTTAAACCACC
ELF1	AT5G46030	RT-qPCR	CTGCTCCTACGAAGCGAATGG	CGACACTAGACCCGTGATTGC

LEA4-1	AT1G32560	RT-qPCR	TTATGGGACACGGTTACGGG	GTCCCGTCGGAGGATAAGTC
MFT	AT1G18100	RT-qPCR	TTGTCGTGGATATTCCCGGAGG	CCACTGGTGGCCTTGGTTCC
RAB18	AT5G66400	RT-qPCR	GACTGAAGGCTTTGGAACTGGC	CAAGCCACCACCACTTTCCTTG
YL2	AT5G25280	RT-qPCR	CTGCTTCGCTCCGATCCAAATC	TGACGACGACGGAGATGATGAC
STZ	AT1G27730	RT-qPCR	ACTAGCCACGTTAGCAGTAGCC	TTCAGGGATCGGAGGGATGTTG
ABI5	AT2G36270	RT-qPCR	GTATCCACCGGTTACTACGAACCTG	CAAGTCGGTTCATCGCCAAATTGGG
NIA1	AT1G77760	RT-qPCR	AATTCGCTTTGGGAACTGCACC	GCACCTCTTCCTCGTCTTATGC

Supplementary Table 2. 3'RNA-seq statistics.

			deduplicated counts (sequenced reads)			
experiment	figure	condition	rep1	rep2	rep3	rep4
imbibition time course	Fig. 1	dry	665211(2383329)	968211(3046500)	1206607(3661820)	1297344(4005374)
imbibition time course	Fig. 1	mock 1h	3063063(7357465)	2515223(6017647)	2314663(5654633)	2519109(6332672)
imbibition time course	Fig. 1	mock 3h	1018361(2420458)	6254(386704)	1333362(3690473)	1833581(4789334)
imbibition time course	Fig. 1	mock 6h	984196(2984969)	1730700(5008179)	1568190(4664813)	1371042(4011951)
imbibition time course	Fig. 1	mock 12h	2008797(5526177)	3372140(8644860)	2326527(6153591)	2499440(6555774)
imbibition time course	Fig. 1	alpha-amanitin 1h	2769568(6733673)	1881349(4641931)	3021111(7014609)	1601689(3984681)
imbibition time course	Fig. 1	alpha-amanitin 3h	1120771(3124610)	1381748(3740320)	765337(2095767)	961781(2690525)
imbibition time course	Fig. 1	alpha-amanitin 6h	1650555(4860783)	825274(2854754)	855685(2786317)	1241632(3485643)
imbibition time course	Fig. 1	alpha-amanitin 12h	792324(3055930)	1813182(4850665)	1937647(5490228)	1499645(4197327)
ABA response	Fig. S4	mock + ABA 6h	1229555(3737462)	2437139(6855570)	2557865(7433885)	1478386(4129656)
ABA response	Fig. S4	alpha-amanitin + ABA 6h	1324312(4095335)	653200(2249465)	967094(3202516)	1220199(3716344)
spike new transcription	Fig. 2	1h input	3510033(6334458)	1937522(3565124)	2209356(4042654)	3726295(6746931)
spike new transcription	Fig. 2	3h input	2400702(4499649)	2010979(3829275)	2492327(4732049)	1357532(2560588)
spike new transcription	Fig. 2	6h input	1321572(2571327)	1570598(3017057)	1132230(2335561)	1873299(3735346)
spike new transcription	Fig. 2	1h new	673334(1463812)	920256(2139443)	514711(1114150)	670804(1453869)
spike new transcription	Fig. 2	3h new	928328(2061754)	1214875(2778032)	1521644(3105970)	2043508(4124322)
spike new transcription	Fig. 2	6h new	4312111(8390584)	2127910(4279812)	1692452(3694603)	3930083(7885831)
first new transcription	Fig. S2	1h input	1231116(4744572)	1256432(4818075)	1368513(5481238)	1759944(6813608)
first new transcription	Fig. S2	3h input	1085929(4520077)	993475(4008892)	488169(2088172)	753656(2936703)
first new transcription	Fig. S2	1h new	60026(483993)	50037(419228)	67234(724250)	77518(596431)
first new transcription	Fig. S2	3h new	588585(3509996)	293974(1774229)	234954(1594697)	736049(4322850)
pulse new transcription	Fig. S2	2.5h + 30 min input	2614706(4611064)	2277099(4197633)	2486109(4471115)	2905490(5109565)
pulse new transcription	Fig. S2	5.5h + 30 min input	1737570(3241825)	1765437(3367944)	1246973(2504633)	2042738(3892045)
pulse new transcription	Fig. S2	2.5h + 30 min new	1521254(2524425)	769660(1295296)	10070380(17308270)	1006712(1671849)
pulse new transcription	Fig. S2	5.5h + 30 min new	1130174(1896021)	2079049(3488273)	1060627(1799784)	1160460(1973566)
conversion	Fig. S2	input not modified	3201375(7025789)	3839445(8351491)	4031162(8777168)	2681351(5814674)
conversion	Fig. S2	input converted	5003821(11083144)	4227749(9274038)	3531049(7651407)	4139977(8944778)

conversion	Fig. S2	enriched converted	2740475(9342402)	1266144(4450934)	2824243(10185817)	2456310(8557039)
CHX new transcription	Fig. 3	mock 3h input	2018633(5321599)	1857234(4830083)	572512(1705253)	
CHX new transcription	Fig. 3	mock 6h input	3615254(8395386)	2085941(4807871)	1759814(3954277)	
CHX new transcription	Fig. 3	CHX 3h input	3007919(7941747)	2153888(5696983)	2672061(6898404)	
CHX new transcription	Fig. 3	CHX 6h input	4287788(6416081)	2154756(4850503)	1716471(3821523)	
CHX new transcription	Fig. 3	mock 3h new	347072(4068563)	425766(4958630)	503593(5274320)	
CHX new transcription	Fig. 3	mock 6h new	1678811(8514030)	528133(2758876)	430323(2266656)	
CHX new transcription	Fig. 3	CHX 3h new	520690(5436775)	333957(3440066)	407238(4507428)	
CHX new transcription	Fig. 3	CHX 6h new	1786455(5075566)	613906(3152513)	501870(2554828)	
PUR new transcription	Fig. S3	mock 6h input	2346361(4519100)	2494177(4869333)	2334605(4475856)	
PUR new transcription	Fig. S3	CHX 6h input	3391892(6355997)	2418387(4523769)	1866964(3487753)	
PUR new transcription	Fig. S3	PUR 6h input	2270637(4201850)	2751286(5121393)	2428466(4524703)	
PUR new transcription	Fig. S3	mock 6h new	2121737(6132082)	1263354(3633877)	3502205(9899962)	
PUR new transcription	Fig. S3	CHX 6h new	1447762(4217527)	1626415(4594663)	3061723(8708974)	
PUR new transcription	Fig. S3	PUR 6h new	2325626(6442324)	1931062(5713228)	3880794(11088342)	
CHX stability	Fig. S3	dry	3505754(5716771)	2342404(3631879)	2727541(4573923)	2770178(4282417)
CHX stability	Fig. S3	alpha-amanitin 1h	5194222(8185940)	4643256(6901133)	2643594(4075004)	4293734(6522056)
CHX stability	Fig. S3	alpha-amanitin 3h	3354041(6235239)	2127014(3458178)	2671604(4757726)	3096954(5495495)
CHX stability	Fig. S3	alpha-amanitin 6h	2228565(4317579)	2656611(5081897)	3907000(7165721)	3315050(5951035)
CHX stability	Fig. S3	CHX + alpha-amanitin 1h	3097308(4760490)	3311070(5042682)	4089090(5971906)	2462040(3869628)
CHX stability	Fig. S3	CHX + alpha-amanitin 3h	3046982(4927479)	5363122(9555877)	3945311(6687847)	4530766(7499021)
CHX stability	Fig. S3	CHX + alpha-amanitin 6h	5162678(9202573)	3740443(6879044)	2824123(5061063)	4333894(7334246)
new transcription imbibition temp	Fig. 4	imbibition 4C input	1491296 (4442853)	1911449(5616077)	2345452(6863805)	2751524(7930121)
new transcription imbibition temp	Fig. 4	imbibition 22C input	2538938(7961371)	1824121(5543482)	3031550(9260947)	1532696(4700494)
new transcription imbibition temp	Fig. 4	imbibition 35C input	1360283(4062516)	1426004(4271395)	631668(1928152)	1535046(5877602)
new transcription imbibition temp	Fig. 4	imbibition 4C new	1082938(8352496)	223558(1866922)	116724(976450)	868550(6996355)
new transcription imbibition temp	Fig. 4	imbibition 22C new	1668129(12226265)	299197(2386241)	1042645(8127145)	876692(6963408)
new transcription imbibition temp	Fig. 4	imbibition 35C new	375692(2916202)	614989(4889662)	246019(1949840)	345768(2977764)
ABA new transcription	Fig. 4	mock 6h input	1221813 (3352714)	1747800(5656309)	3215674(8873416)	2987734(7852835)
ABA new transcription	Fig. 4	mock 6h new	1407907(3020212)	1148857(2535677)	343896(855213)	1262730(2633486)
ABA new transcription	Fig. 4	ABA 6h input	2594087(7325252)	1641058(5029787)	2831613(8282358)	760657(2019595)

ABA new transcription	Fig. 4	ABA 6h new	1692086(3516430)	776441(1687788)	1911808(4058015)	1284126(2863324)
ANT new transcription	Fig. 4	mock 6h input	29645(132445)	1203013(4770308)	1298764(5256040)	661356(2768424)
ANT new transcription	Fig. 4	mock 6h new	1863286(10641788)	1061140(6563138)	590498(3560449)	962525(5737157)
ANT new transcription	Fig. 4	ANT 6h input	1070613(4594354)	674130(2875481)	924478(3747151)	663959(2781314)
ANT new transcription	Fig. 4	ANT 6h new	1165074(6592421)	754943(4644481)	611902(4644481)	1102275(6066267)
new transcription maturation temp	Fig. S5	dry 15C	2825449(7167531)	2406187(6225625)	2149880(5613564)	
new transcription maturation temp	Fig. S5	dry 22C	2260406(5788408)	2304645(5943874)	1544727(4002121)	
new transcription maturation temp	Fig. S5	dry 26C	1461207(3807859)	2663587(7003808)	1082410(2957705)	
new transcription maturation temp	Fig. S5	maturation 15C input	2966984(7277469)	1967891(4877570)	1990149(4987510)	
new transcription maturation temp	Fig. S5	maturation 22C input	1584609(4079081)	3218715(8074480)	859199(2306826)	
new transcription maturation temp	Fig. S5	maturation 26C input	1369743(3672108)	1913049(5137231)	1749603(4868720)	
new transcription maturation temp	Fig. S5	maturation 15C new	316074(2298503)	349743(2582193)	296190(2159904)	
new transcription maturation temp	Fig. S5	maturation 22C new	827785(5994688)	1322252(9393715)	956331(6831045)	
new transcription maturation temp	Fig. S5	maturation 26C new	540345(3925657)	256021(1893122)	314783(2431862)	
B. napus	Fig. 5	dry	1212302(6812422)	1263788(7290791)	1364267(7869326)	1212624(6612787)
B. napus	Fig. 5	4h input	616481(3112504)	591398(3160144)	661384(3465694)	407065(2441378)
B. napus	Fig. 5	8h input	756881(4545327)	451871(2863441)	530204(3512689)	631319(4184681)
B. napus	Fig. 5	4h new	333537(1872812)	329049(1859729)	274412(1521131)	256927(1380207)
B. napus	Fig. 5	8h new	2373724(10009228)	1098920(5152017)	555150(2523257)	1214900(5041595)

Supplementary Table 3. smFISH probes list.

	ICL (AT3G21720)	ssLEA (AT3G17520)
1	CTGCATTGGCAAATATTGCT	TCTAACCCCATTTTTGTTAC
2	CCCTATCATCTCCTTATATA	AACCAAACCGTACACTTTCC
3	AGCTTCAAGCATATTTGCTC	CCATAAGTACCAAAGATACC
4	TGCTTTGTTAGGAGCTATGA	TTCTTCCTCGATCGTAGCTT
5	CCATTATCATAGAGGGGACA	AATCAGTCCATGATTCATCC
6	TTAGCTTGAACCTCTCTGAG	TGCTTGAGACCGATCTTCTC
7	CTTGAGATGGCCACGTAGAG	TGAGTGGGTTGGATGTTGTC
8	TTGGCTTGATGGCTTTTGAG	CGTCTTGAACGGTCGTGGTG
9	TGTCCAAATGTTTAGCCATC	TTTTTGACTCGCTCTCCAAG
10	TGCCAACCAGAGACATAGAT	TTCCTCTGCTTTGCGTTTAG
11	TAGTGGATGTGTGAGTGGAC	TAGCTGTCTCGTATGCCGAA
12	ATCAGCAAGATCAGGACCAG	GGCCTTGTCTTTTACAGAAG
13	TGTTAGGAACGGTGTCGTAC	CTTAACTTGACCAGCTGAGT
14	GAGCGAAGAAGAGGTGTTCA	TCCCGACAAGCTATCTTTAA
15	TGCTTTCTGTCATGGTACTG	TCCAAGACTCATCGTTTTCG
16	TCTGCTCATGCTCATTCTTG	GATTTTCTCTTTGGCCCAAC
17	CACGAACGGAGTTTTTGTCC	ATACCGTCTCTCCCAAGTTA
18	CGATGATGGGCTTTAGGTAG	TTAGCTTCTTTTGCCTTCTC
19	GCCTCTTTCAACGAAAAGCT	AACTTCTCTTTAGCATCTCC
20	CACACTTCTTGGTGACGGAG	TTCTCTTTCGCCGTCTCAAC
21	ACAAGGCGGTTGATGTGTTC	CTTTAATTTTCTCCTTCGCA
22	CAACAAGGACGGTCTCTGTA	CTTTCGCGGACTCTAAAGTC
23	ATGTTCGACTGGATCAGAGT	TGAGTCGTAACTCTGCGACG
24	TTAGTGGCACCGAGGATGAA	TTGACTTGTGAGACACGGTA
25	AGCCAGAAGCGAGGACAAAC	GTCAAGCTCTCTTTAACACG
26	ATTCTTGCCTACAGTCATTC	TCAGAGCTCAGCATCATCGT
27	CACGACAGCTTCCGAGAAAG	TCGATGAGTTTCTCCCAACT
28	TGAGGTTCATGCGCTTGATG	ACGTAATGTGTATATTCCGA
29	TCAGTCTCTGATTCTTCTCG	TCACTAATGGGCCCGATTAG
30	CATACCTTGCATGGGTTAAC	TAGAAGACTTGGGCCACTAT
31	CCAAGTTTTGCTGCTAACAC	TAGCAAGTGTGCTGCTTCTG
32	GTCCCAGAAAAGATCTGTCA	TGACCAAATAACGAGTTCGT
33	CAGATGATGTCTGCGATCTG	
34	TGGGTGCATTCATTGAGATC	
35	TGTCTTGGACTTGATACCTT	
36	AGAGATTGTAGGCGAGCATG	
37	AATATCCGAGCCTAGCAATC	
38	AAACCCGCAAGCGTTATGAA	
39	TATCAACCACAAGAGCATCC	
40	CTAGCGTAATCCTTTGCAAA	
41	TTTGTATCCTCTCCACATAA	
42	TGGTGAGCCAAAGTGTCAAC	

43	ATAGTAATTAGCACCGGACC	
44	TTCTTCAGTGACACCTTTTC	
45	TTGTCCAACTCTCCTTGAAC	
46	CTTACATTCTTGACTTGGCG	
47	CACCGCCGTAATGAACATAT	
48	TTCCAAGCTTTCCTACGAAG	
49	AAATATTGCCCCAAACCTTT	
50	ATACAAAGAAGCAAACCCCA	
51	GCCAATAACACCAAATTCCA	
