# Supplementary material for: Transcriptional activity during seed imbibition is conserved, robust and required for germination
Source: Nat Commun. 2026 Aug 19;17:9916. doi: 10.1038/s41467-026-76795-8 (PMC13582861; doi:10.1038/s41467-026-76795-8)
Supplement: Supplementary file 3 [file 41467_2026_76795_MOESM9_ESM.pdf]

nature portfolio

Corresponding author(s): Michal Krzyszton
Szymon Swiezewski

Last updated by author(s): Jul 24, 2026

Reporting Summary

Nature Portfolio wishes to improve the reproducibility of the work that we publish. This form provides structure for consistency and transparency in reporting. For further information on Nature Portfolio policies, see our Editorial Policies and the Editorial Policy Checklist.

Statistics

For all statistical analyses, confirm that the following items are present in the figure legend, table legend, main text, or Methods section.

n/a	Confirmed	
☐	☒	The exact sample size (*n*) for each experimental group/condition, given as a discrete number and unit of measurement
☐	☒	A statement on whether measurements were taken from distinct samples or whether the same sample was measured repeatedly
☐	☒	The statistical test(s) used AND whether they are one- or two-sided<br>*Only common tests should be described solely by name; describe more complex techniques in the Methods section.*
☒	☐	A description of all covariates tested
☐	☒	A description of any assumptions or corrections, such as tests of normality and adjustment for multiple comparisons
☐	☒	A full description of the statistical parameters including central tendency (e.g. means) or other basic estimates (e.g. regression coefficient) AND variation (e.g. standard deviation) or associated estimates of uncertainty (e.g. confidence intervals)
☐	☒	For null hypothesis testing, the test statistic (e.g. *F*, *t*, *r*) with confidence intervals, effect sizes, degrees of freedom and *P* value noted<br>*Give P values as exact values whenever suitable.*
☒	☐	For Bayesian analysis, information on the choice of priors and Markov chain Monte Carlo settings
☒	☐	For hierarchical and complex designs, identification of the appropriate level for tests and full reporting of outcomes
☒	☐	Estimates of effect sizes (e.g. Cohen's *d*, Pearson's *r*), indicating how they were calculated

Our web collection on statistics for biologists contains articles on many of the points above.

Software and code

Policy information about availability of computer code

Data collection	Sequencings were performed using Illumina NovaSeq 6000, and qPCR analysis was performed with Roche LightCycler 480.
Data analysis	3’RNA-seq reads were processed with BRBseqTools (v 1.6) and mapped with STAR (v 2.7.8a).<br>ChIP-seq:<br>Adapter sequences were trimmed with cutadapt (v 1.18).<br>Reads were mapped using bowtie2 (v 2.3.5.1).<br>Mapped reads were processed using samtools (v 1.13), filtered with sambamba (v 0.8.0), and duplicated reads were removed with gatk (v 4.2.6.1). Normalised bigwig tracks were prepared using bedtools (v 2.30.0) and bedGraphToBigWig (v 4). Bigwig tracks normalised for the input were generated using bigwigCompare (v 3.5.1). Peaks were called using macs2 (v 2.2.7.1).<br>For smFISH analysis, cells were manually segmented in Napari using the PartSeg plugin, which enables foci identification and classification.<br>The final data analysis for 3’RNA-seq, ChIP-seq, and smFISH was performed using various R packages, as detailed in https://github.com/mk1859/seed_transcription.

For manuscripts utilizing custom algorithms or software that are central to the research but not yet described in published literature, software must be made available to editors and reviewers. We strongly encourage code deposition in a community repository (e.g. GitHub). See the Nature Portfolio guidelines for submitting code & software for further information.

Data

Policy information about availability of data

All manuscripts must include a data availability statement. This statement should provide the following information, where applicable:

- Accession codes, unique identifiers, or web links for publicly available datasets
- A description of any restrictions on data availability
- For clinical datasets or third party data, please ensure that the statement adheres to our policy

Raw data of RNA-seq and ChIP-seq are deposited in the NCBI Gene Expression Omnibus under accession number GSE297302 [https://www.ncbi.nlm.nih.gov/geo/query/acc.cgi?acc=GSE297302]. smFISH analysis results can be found at https://github.com/mk1859/seed_transcription

Research involving human participants, their data, or biological material

Policy information about studies with human participants or human data. See also policy information about sex, gender (identity/presentation), and sexual orientation and race, ethnicity and racism.

Reporting on sex and gender	*Use the terms sex (biological attribute) and gender (shaped by social and cultural circumstances) carefully in order to avoid confusing both terms. Indicate if findings apply to only one sex or gender; describe whether sex and gender were considered in study design; whether sex and/or gender was determined based on self-reporting or assigned and methods used.* *Provide in the source data disaggregated sex and gender data, where this information has been collected, and if consent has been obtained for sharing of individual-level data; provide overall numbers in this Reporting Summary. Please state if this information has not been collected.* *Report sex- and gender-based analyses where performed, justify reasons for lack of sex- and gender-based analysis.*
Reporting on race, ethnicity, or other socially relevant groupings	*Please specify the socially constructed or socially relevant categorization variable(s) used in your manuscript and explain why they were used. Please note that such variables should not be used as proxies for other socially constructed/relevant variables (for example, race or ethnicity should not be used as a proxy for socioeconomic status).* *Provide clear definitions of the relevant terms used, how they were provided (by the participants/respondents, the researchers, or third parties), and the method(s) used to classify people into the different categories (e.g. self-report, census or administrative data, social media data, etc.)* *Please provide details about how you controlled for confounding variables in your analyses.*
Population characteristics	*Describe the covariate-relevant population characteristics of the human research participants (e.g. age, genotypic information, past and current diagnosis and treatment categories). If you filled out the behavioural & social sciences study design questions and have nothing to add here, write "See above."*
Recruitment	*Describe how participants were recruited. Outline any potential self-selection bias or other biases that may be present and how these are likely to impact results.*
Ethics oversight	*Identify the organization(s) that approved the study protocol.*

Note that full information on the approval of the study protocol must also be provided in the manuscript.

Field-specific reporting

Please select the one below that is the best fit for your research. If you are not sure, read the appropriate sections before making your selection.

- [x] Life sciences
- [ ] Behavioural & social sciences
- [ ] Ecological, evolutionary & environmental sciences

For a reference copy of the document with all sections, see nature.com/documents/nr-reporting-summary-flat.pdf

Life sciences study design

All studies must disclose on these points even when the disclosure is negative.

Sample size	No statistical approach was used to predetermine sample size. The sample size for almost all experiments was 3-4, except for the germination experiment in the presence of alpha-amanitin, which used 8 replicates. These sample sizes are standard in seed biology studies.
Data exclusions	Libraries with too few reads, caused by technical issues, were omitted from the analysis. That is described on GitHub [https://github.com/mk1859/seed_transcription] in the step-by-step data analysis description.
Replication	We replicated several experiments aimed at identifying new mRNAs, which yielded highly consistent results (Supplementary Fig. 9).
Randomization	Plants from which we collected seeds were grown in random locations in growing chambers. Collected seed samples were randomly assigned a pool number.
Blinding	Blinding was not required, as for each experiment samples were handled simultaneously under identical conditions, eliminating the possibility of bias.

Reporting for specific materials, systems and methods

We require information from authors about some types of materials, experimental systems and methods used in many studies. Here, indicate whether each material, system or method listed is relevant to your study. If you are not sure if a list item applies to your research, read the appropriate section before selecting a response.

Materials & experimental systems

n/a	Involved in the study
☐	☒ Antibodies
☒	☐ Eukaryotic cell lines
☒	☐ Palaeontology and archaeology
☒	☐ Animals and other organisms
☒	☐ Clinical data
☒	☐ Dual use research of concern
☐	☒ Plants

Methods

n/a	Involved in the study
☐	☒ ChIP-seq
☒	☐ Flow cytometry
☒	☐ MRI-based neuroimaging

Antibodies

Antibodies used

rabbit polyclonal antibody anti-RPB1 antibody (Agrisera AS11 1804); Lot 2003 - ChIP (2.5 ug per IP)
rabbit polyclonal antibody anti-mCherry antibody (Abcam, ab167453) - Western blot (1:2000 dillution)
Mouse anti-Phospho RNA polymerase II CTD repeat YSPTSPS antibody pS5 (Abcam ab5408) - immunolocalisation (1:100 dillution)
Rabbit anti-Phospho RNA Polymerase II CTD repeat YSTPSTS antibody pS2 (Abcam ab5095) - immunolocalisation (1:100 dillution)
Goat anti-Rabbit IgG (H+L) Alexa Fluor Plus 488 (ThermoFisher A-11008) - secondary Ab immunolocalisation (1:100 dillution)
Goat anti-Mouse IgG (H+L) Alexa Fluor Plus 488 (ThermoFisher A-11001) - secondary Ab immunolocalisation (1:100 dillution)

Validation

Pol II antibody was validated earlier by us (information on the producer's website - https://www.agrisera.com/en/artiklar/polymerase-ii.html). In the scope of this work, we also checked it using ChIP-qPCR (Supplementary Fig. 7b).
mCherry antibody is commonly used and verified multiple times as stated on producers website (https://www.abcam.com/en-us/products/primary-antibodies/mcherry-antibody-ab167453).

Dual use research of concern

Policy information about dual use research of concern

Hazards

Could the accidental, deliberate or reckless misuse of agents or technologies generated in the work, or the application of information presented in the manuscript, pose a threat to:

No	Yes
☒	☐ Public health
☒	☐ National security
☒	☐ Crops and/or livestock
☒	☐ Ecosystems
☒	☐ Any other significant area

Experiments of concern

Does the work involve any of these experiments of concern:

No	Yes
☒	☐ Demonstrate how to render a vaccine ineffective
☒	☐ Confer resistance to therapeutically useful antibiotics or antiviral agents
☒	☐ Enhance the virulence of a pathogen or render a nonpathogen virulent
☒	☐ Increase transmissibility of a pathogen
☒	☐ Alter the host range of a pathogen
☒	☐ Enable evasion of diagnostic/detection modalities
☒	☐ Enable the weaponization of a biological agent or toxin
☒	☐ Any other potentially harmful combination of experiments and agents

Plants

Seed stocks	Col-0 Arabidopsis was originally obtained from the NASC repository. Seeds of the Brassica napus Bazyl cultivar were obtained from the seed producer Obrol.
Novel plant genotypes	No novel genotypes were generated in this work.
Authentication	We used wild-type plants - authentication was not required.

ChIP-seq

Data deposition

- [x] Confirm that both raw and final processed data have been deposited in a public database such as GEO.
- [x] Confirm that you have deposited or provided access to graph files (e.g. BED files) for the called peaks.

Data access links<br>*May remain private before publication.*	Raw data of RNA-seq and ChIP-seq are deposited in the NCBI Gene Expression Omnibus under accession number GSE297302 [https://www.ncbi.nlm.nih.gov/geo/query/acc.cgi?acc=GSE297302].
Files in database submission	raw files:<br>dry_1_R1_001.fastq.gz<br>dry_1_R2_001.fastq.gz<br>dry_2_R1_001.fastq.gz<br>dry_2_R2_001.fastq.gz<br>dry_3_R1_001.fastq.gz<br>dry_3_R2_001.fastq.gz<br>dry_in_1_R1_001.fastq.gz<br>dry_in_1_R2_001.fastq.gz<br>dry_in_2_R1_001.fastq.gz<br>dry_in_2_R2_001.fastq.gz<br>dry_in_3_R1_001.fastq.gz<br>dry_in_3_R2_001.fastq.gz<br>imb_1_R1_001.fastq.gz<br>imb_1_R2_001.fastq.gz<br>imb_2_R1_001.fastq.gz<br>imb_2_R2_001.fastq.gz<br>imb_3_R1_001.fastq.gz<br>imb_3_R2_001.fastq.gz<br>imb_in_1_R1_001.fastq.gz<br>imb_in_1_R2_001.fastq.gz<br>imb_in_2_R1_001.fastq.gz<br>imb_in_2_R2_001.fastq.gz<br>imb_in_3_R1_001.fastq.gz<br>imb_in_3_R2_001.fastq.gz<br><br>processed files:<br>dry_1.bigwig<br>dry_2.bigwig<br>dry_3.bigwig<br>dry_in_1.bigwig<br>dry_in_2.bigwig<br>dry_in_3.bigwig<br>imb_1.bigwig<br>imb_2.bigwig<br>imb_3.bigwig<br>imb_in_1.bigwig<br>imb_in_2.bigwig<br>imb_in_3.bigwig<br>dry_1.broadPeak<br>dry_2.broadPeak<br>dry_3.broadPeak<br>imb_1.broadPeak

imb_2.broadPeak
imb_3.broadPeak

Genome browser session (e.g. UCSC)

Provide a link to an anonymized genome browser session for "Initial submission" and "Revised version" documents only, to enable peer review. Write "no longer applicable" for "Final submission" documents.

Methodology

Replicates

Three biological replicates of IP from dry and 6h-imbibed seeds were analysed, along with and the input samples. Replicates clustered together when analysed using multiBamSummary with TAIR10 protein-coding genes as the bed file input.

Sequencing depth

ChIP-seq was performed using NovaSeq 6000, obtaining on average 51 M paired-end 100 bp reads for IP samples, and 41 M reads for input samples. Quality of reads was verified using FastQC, and low-quality mapped reads were removed with sambamba.

Antibodies

polyclonal rabit anti-RPB1 antibody (Agrisera AS11 1804); Lot 2003 (2.5 ug per IP)

Peak calling parameters

Reads were mapped using bowtie2 (v 2.3.5.1). Peaks were called using macs2 (v 2.2.7.1) with default settings and the broad option using inputs as controls.

Data quality

Peaks were identified for each replicate, with only those present in at least two replicates reported. ChIP using seed material is a highly challenging technique; most current seed studies perform ChIP for histone modifications, which are easier due to strong DNA interactions. We are the first to show Pol II ChIP-seq results in dry and briefly imbibed seeds. Limited Pol II ChIP-seq signals in dry seeds are consistent with immunolocalization data and the known compactness of chromatin in dry seeds. Pol II levels rise after 6 hours of imbibition, aligning with shown by us transcription restart. However, technical limitations result in a relatively small number of peaks and their weak enrichment. Notably, genes with Pol II binding in their gene bodies tend to overlap with genes having open promoter regions identified by ATAC-seq, suggesting the Pol II signal is transcription-related. We also confirmed Pol II binding to selected genes via ChIP-qPCR. Ultimately, these Pol II ChIP-seq results support findings from other methods.

Software

Adapter sequences were trimmed with cutadapt (v 1.18).
Reads were mapped using bowtie2 (v 2.3.5.1).
Mapped reads were processed using samtools (v 1.13), filtered with sambamba (v 0.8.0), and duplicated reads were removed with gatk (v 4.2.6.1). Normalised bigwig tracks were prepared using bedtools (v 2.30.0) and bedGraphToBigWig (v 4). Bigwig tracks normalised for the input were generated using bigwigCompare (v 3.5.1). Peaks were called using macs2 (v 2.2.7.1).
